# Synthesis of 1‑Fluoroalkyl-5-Substituted-1,2,3-Triazoles from Carbonyl-Stabilized Phosphonium Ylides

**DOI:** 10.1021/acs.joc.5c01055

**Published:** 2025-08-11

**Authors:** David Tichý, Tomáš Černý, Josef Filgas, Svatava Voltrová, Blanka Klepetářová, Petr Beier

**Affiliations:** † 89220 Institute of Organic Chemistry and Biochemistry of the Czech Academy of Sciences, Flemingovo nám. 2, Prague 160 00, Czechia; ‡ Charles University, Faculty of Science, Albertov 6, Prague 128 00, Czechia; § Institute of Organic Chemistry and Technology, University of Pardubice, Studentská 573, Pardubice 532 10, Czechia; ∥ Department of Physical Chemistry, University of Chemistry and Technology, Technická 5, Prague 166 28, Czechia

## Abstract

A mild, rapid, and regioselective cyclization of azidofluoroalkanes with carbonyl-stabilized phosphonium ylides, resulting in the formation of 1-fluoroalkyl-5-substituted-1,2,3-triazoles, is presented. The synthetic method tolerates air and water, works at room temperature in a benign solvent, and is metal-free. Both starting materials are commercially available and easily prepared. The cyclization method was expanded to 4-halogen-substituted analogues of target triazoles, which underwent either rhodium-catalyzed transannulation with benzonitrile to yield uniquely substituted *N*-fluoroalkylated 4-haloimidazoles or cross-coupling reactions to produce fully substituted *N*-fluoroalkylated triazoles.

## Graphical abstract

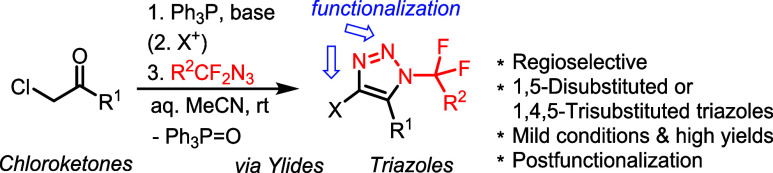

## Introduction

Since the discovery of copper­(I)-catalyzed azide–alkyne cycloaddition (CuAAC), a reaction of unprecedented robustness, reliability, and efficiency, the products 4-substituted 1,2,3-triazoles experienced a renewed interest in the scientific community.^1–3^ 1,2,3-Triazoles are utilized in drug development,^4–6^ agrochemicals,^7^ material chemistry,^8^ bioconjugation,^9,10^ and organic synthesis.^11,12^

However, 5-substituted-1,2,3-triazoles are not accessible by CuAAC. Several methods have been reported for their synthesis. Metal (Li, Mg, and Zn) acetylides react with organic azides on the terminal nitrogen, resulting in the formation of 4-metalated-1,5-disubstituted 1,2,3-triazole species. Protodemetalation or a reaction with other electrophiles yields triazoles substituted at the 1,5- or 1,4,5-positions, respectively (Scheme 1A).^13–15^ The main disadvantages of this procedure are the low functional group tolerance and the necessity to work under inert conditions. Strongly basic Me_4_NOH was also used for the synthesis of 5-substituted triazoles.^16^

**Scheme 1:**
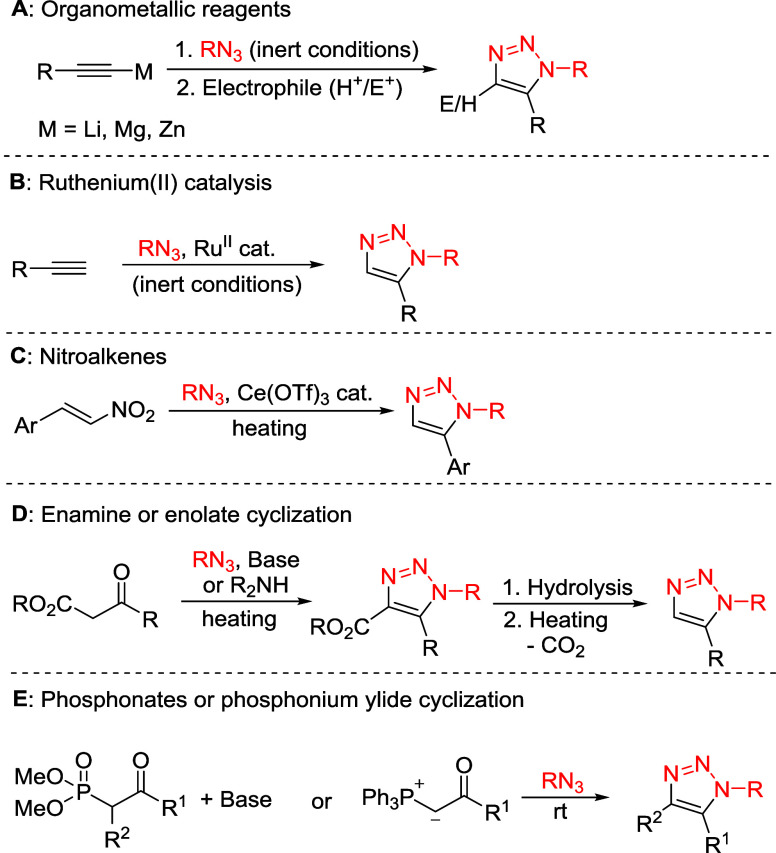
Synthetic Approaches to 1,5-Disubstituted 1,2,3-Triazoles Using Organometallic Reagents (A), Ruthenium­(II) Catalysis (B), Nitroalkenes (C), Enamines or Enolates (D), Phosphonates or Phosphonium Ylides (E)

Furthermore, 1,5-disubstituted triazoles were obtained from terminal alkynes and organic azides using Ru (II) catalysis (Scheme 1B).^17^ While this procedure is a safer alternative to the reaction with pyrophoric organometallics, the Ru catalysts are expensive and require handling under an inert atmosphere. Moreover, the products may contain residual transition metal impurities with detrimental effects on health when used in vivo as drug candidates. An analogous method to the Ru catalysis is the Ni catalysis, which is air- and water-tolerant, but also requires basic conditions and generally provides products of incomplete regiochemistry.^18^ Ce­(III)-catalyzed cycloaddition of nitroolefins and azides provided selectively 1,5-disubstituted triazoles (Scheme 1C).^19,20^

Metal-free synthetic approaches to 1,2,3-triazoles were summarized recently.^21–23^ Relying on 1,3-dipolar cycloadditions of organic azides as dipoles and enamines^24–26^ or enolates^27–29^ as dipolarophiles (often formed in situ), this wide family of reactions provides 1,4,5-trisubstituted 1,2,3-triazoles. When β-ketoesters were used as enolate or enamine precursors, the resulting triazole contained an alkoxycarbonyl group in position four, which can be hydrolyzed and decarboxylated, resulting in 5-substituted triazoles (Scheme 1D).^30^ An infrequently employed 1,3-dipolar cyclization method for the synthesis of a limited range of 1,5-disubstituted triazoles involves the use of carbonyl-stabilized phosphonium ylides^31^ or phosphonates^32,33^ (Scheme 1D). This mild cyclization approach was used in biochemistry as a ligation method for the synthesis of 1,5-disubstituted triazoles as *cis-*amide mimetics in peptides, utilizing polymer-bound phosphonium ylides.^34,35^

Fluorinated organic compounds are widely used in the development of drugs and agrochemicals, in diagnostics, and in polymer science.^36–41^ Over the past several years, we have developed organic α-fluorinated azides^42,43^ and utilized them in various transformations, ranging from CF_3_-nitrene and aziridine formation^44^ to the synthesis of *N*-fluoroalkylated heterocycles by CuAAC (triazoles)^45^ and Rh­(II)-catalyzed transannulation approaches (imidazoles, pyrroles, indoles, and azepines).^46–48^ In this work, we revisit the rarely used cycloaddition of phosphonium ylides, apply it for the regioselective synthesis of new *N*-fluoroalkylated 5-substituted 1,2,3-triazoles, and investigate their transannulation with nitriles to afford *N*-fluoroalkylated imidazoles.

## Results and Discussion

While *N*-fluoroalkylated 4-substituted 1,2,3-triazoles are easily available by CuAAC,^43,45^ the synthesis of 5-substituted analogues is not straightforward. The approach of using lithium acetylides^13^ was only partially successful in the case of azidofluoroalkanes (Scheme 2).

**Scheme 2:**
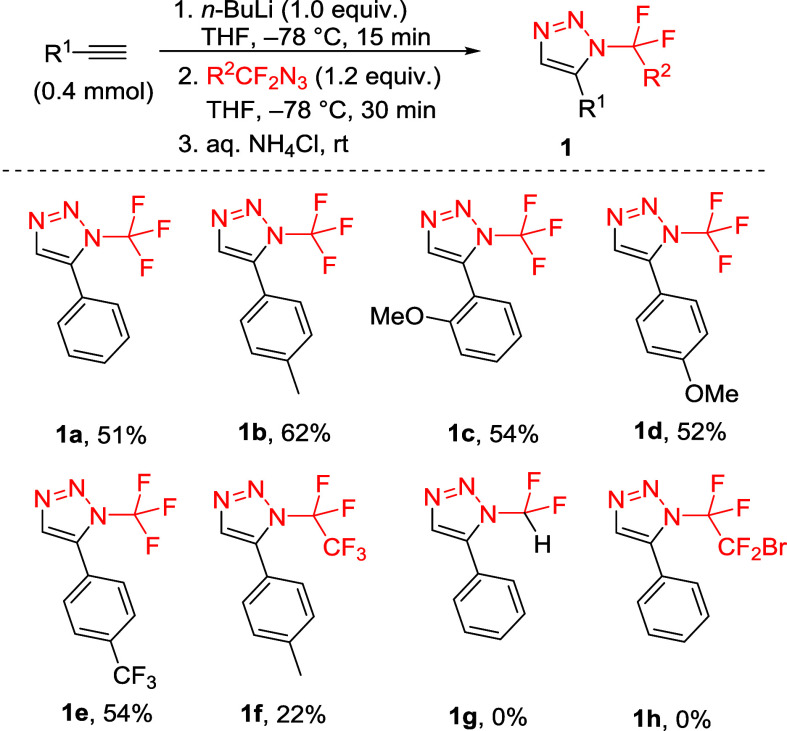
Synthesis of Triazoles 1 Using Lithium Acetylides

High product yields were obtained using CF_3_N_3_, while with CF_3_CF_2_N_3_ a lower reaction efficiency was observed. Importantly, lithium acetylides are strongly basic and nucleophilic, which precluded their use with azides bearing acidic (HCF_2_−) or electrophilic (BrCF_2_CF_2_−) moieties (Scheme 2).

The Ru­(II) catalysis approach^17^ applied to phenylacetylene and azidopentafluoroethane did not yield any triazole product (Scheme 3A). The approach outlined in Scheme 1D was applied to azidofluoroalkanes but suffered from low functional group tolerance and a multistep procedure (Scheme 3B).^30^ Furthermore, this synthetic approach could not be applied to HCF_2_N_3_ or BrCF_2_CF_2_N_3_ due to lack of reactivity or side reactions.

**Scheme 3:**
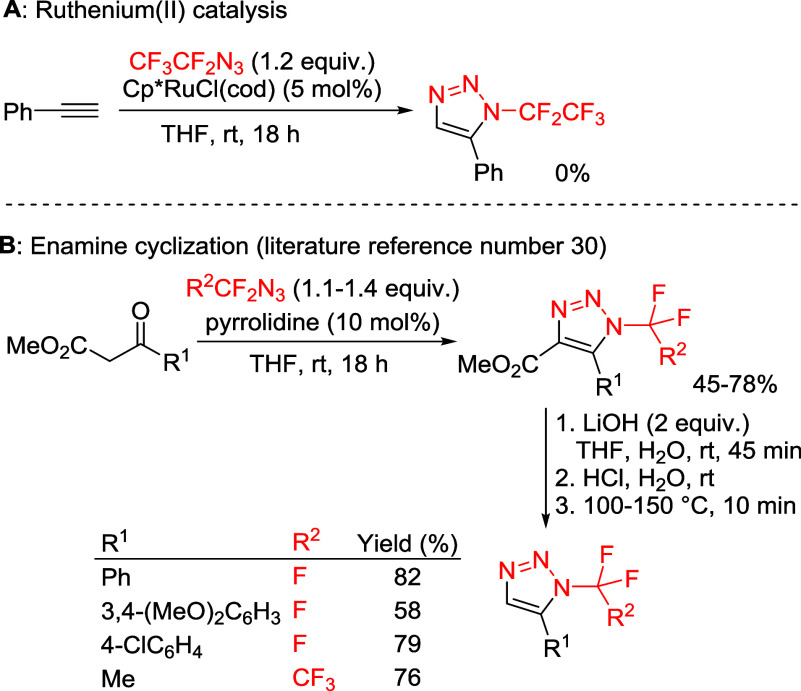
Preliminary Experiments Leading to *N*-Fluoroalkyl-5-substituted-1,2,3-triazoles

Dissatisfied with the results, we decided to investigate phosphonium ylide cyclization for the synthesis of 5-substituted triazoles. Carbonyl-stabilized phosphonium ylides are bench-stable compounds, affording 1,5-disubstituted triazoles in reactions with sulfonyl or acyl azides. The reaction was inefficient with phenyl azide.^31^ We performed the cyclization using in situ-prepared ylides **2** or halogenated ylides **3** derived from α-chloroketones (Scheme 4). The optimal solvent was found to be aqueous acetonitrile, which ensured rapid cyclization reactions (5 min). The side product, triphenylphosphine oxide, is notoriously difficult to remove by chromatography. We circumvented the problem by evaporating the reaction mixture on silica gel and washing the triazole product with diethyl ether. This way, triphenylphosphine oxide was almost completely removed. Subsequent column chromatography provided products of high purity.

**Scheme 4:**
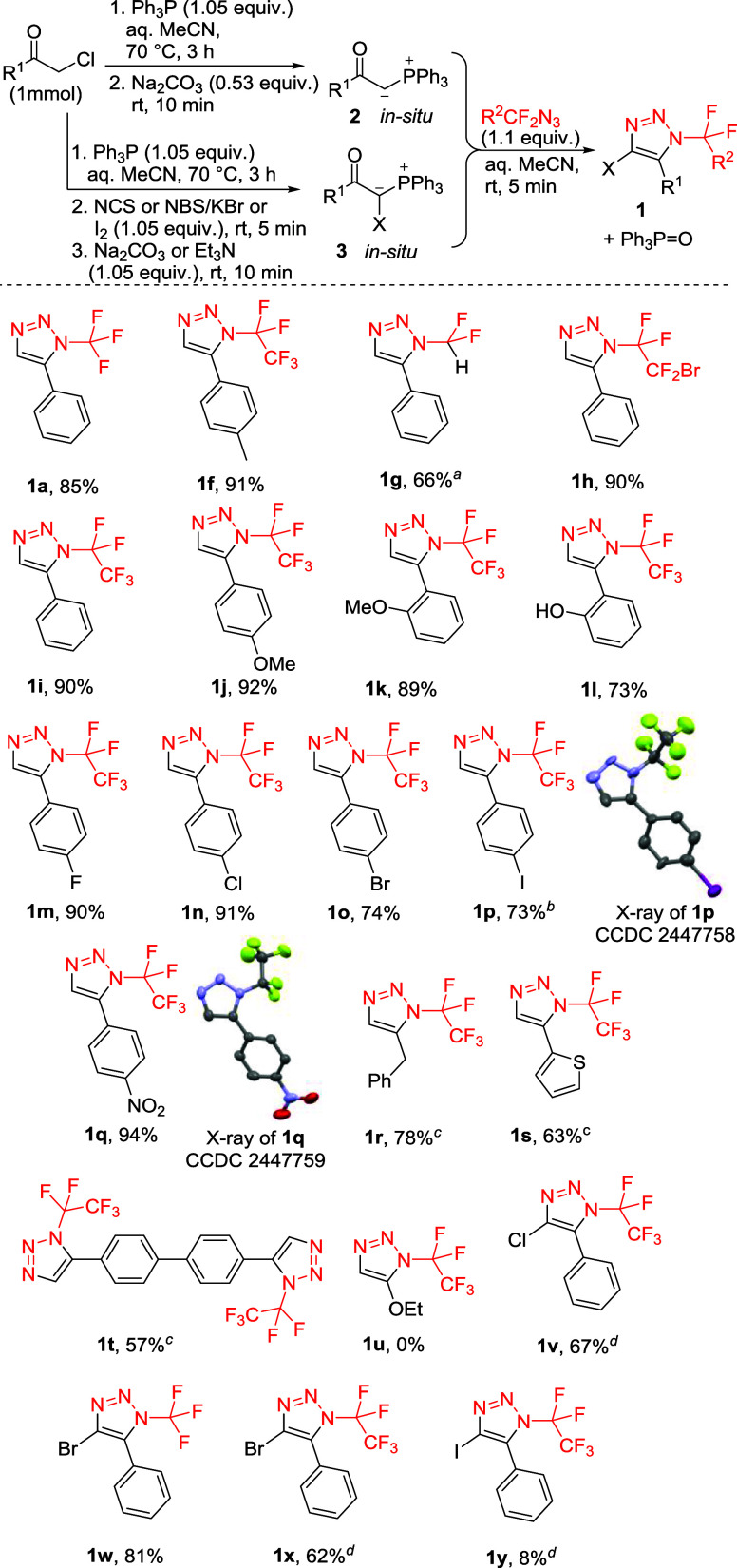
Scope of the Synthesis of *N*-(Per)­Fluoroalkyl-5-substituted 1,2,3-triazoles 1 Using Phosphonium Ylides^a^,^b^,^c^,^d^

a Reaction time 2 h for the reaction of **2** with HCF_2_N_3_.
b Required bromoketone instead of chloroketone.
c Reaction time 24 h for the reaction of chloroketone with Ph_3_P.
d Reaction time 1 h for the reaction of **3** with CF_3_CF_2_N_3_.

In comparison to the reaction with lithium acetylides, the cyclization of in situ-prepared phosphonium ylides with fluoroalkylated azides displayed a wide scope, tolerating previously incompatible azides (products **1g**, **1h**), protic, bulky, or reactive functional groups on the phenyl ring, benzyl, or heterocyclic groups in position five of the triazole ring. The structures of **1p** and **1q** were further confirmed by X-ray crystallography. The ylide prepared from ethyl chloroacetate failed to provide triazole **1u**. Halogens (Cl, Br, I) were conveniently introduced to position four of the triazole ring using halogenated phosphonium ylides in good yields, except for the 4-iodo derivative **1y**. All product yields were calculated based on the starting α-chloroketones (over 3–4 steps, all in one pot).

Interestingly, the 4-iodo derivative **1y** was accessed by the lithiation of triazole **1i** with *n*-BuLi and subsequent iodination (Scheme 5), demonstrating that 4-lithio triazoles are stable at low temperature and nucleophilic. Analogous **1z** was prepared in a similar manner. 4-Halogenated 5-substituted 1,2,3-triazoles are rare compounds, and so far, the only known synthetic method for *N*-fluoroalkyl derivatives, specifically *N*-trifluoromethyl 4-chloro-1,2,3-triazoles, was reported in 2024 by Wang. This method is based on the cyclization of *N*-trifluoromethyl imidoyl chlorides with isocyaniminotriphenylphosphorane (Ph_3_P=N–N≡C).^49^

**Scheme 5:**
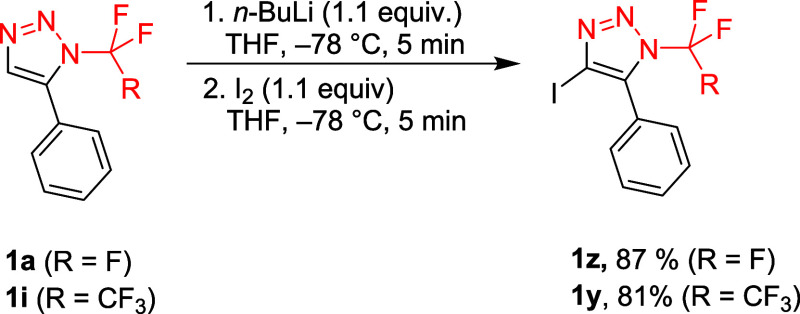
Alternative Synthesis of *N*-Pentafluoroethyl 4-iodo-5-phenyl-1,2,3-triazoles 1y and 1z

For the phosphonium ylide cycloaddition, we propose a reaction mechanism based on a nucleophilic attack of the ylidic carbon on the terminal nitrogen of the fluorinated azide (intermediate **A**), followed by cyclization to form the intermediate **B**. Further cyclization to oxaphosphetane **C** and elimination of triphenylphosphine oxide result in the formation of product **1** (Scheme 6). The cyclization of **A** to **B** must be fast to outcompete fluoride elimination to compound **D**, which was not observed by ^19^F NMR spectroscopy.

**Scheme 6:**
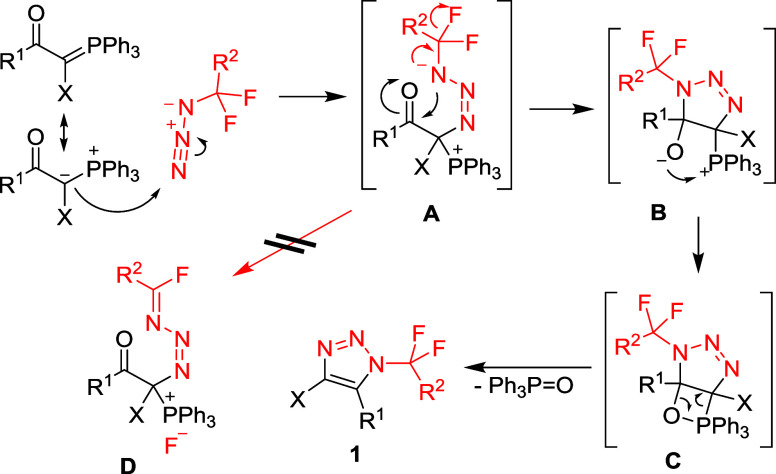
Proposed Reaction Mechanism

With a collection of triazoles **1** in hand, we proceeded to test their reactivity in rhodium­(II)-catalyzed transannulation with benzonitrile to *N*-(per)­fluoroalkylated imidazoles **4**. Triazole **1b** did not yield any expected imidazole product **4b** and decomposed slowly only upon microwave heating with benzonitrile and catalytic Rh_2_(Oct)_4_. We speculated that the presence of a suitable M+ or M– substituent at position four, to stabilize the rhodium carbenoid intermediate **E**, is necessary for the reaction to proceed. Thus, 4-halogenated triazoles **1v**, **1x,** and **1y** afforded uniquely substituted *N*-fluoroalkylated 4-halogenated imidazoles **4**. A trend was observed where the decreased electronegativity and increased polarizability of the halogen in position four of the triazole allowed the reaction to proceed under milder conditions and required lower amounts of the catalyst. Thus, this study established that Rh-carbene transannulation chemistry works not only with aryl-stabilized carbenes but also with halogen-stabilized carbenes (Scheme 7).

**Scheme 7:**
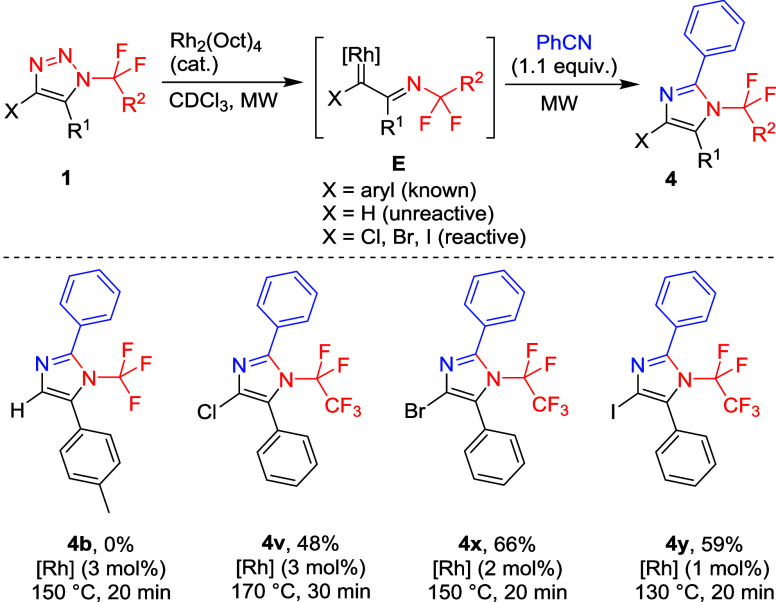
(II)-Catalyzed Transannulation of Triazoles 1

A study of further functionalization of 4-halogenated triazoles showed that the Suzuki–Miyaura reaction of 4-bromo triazoles **1w** or **1x** afforded the coupling products **5** in good yields. On the other hand, the Sonogashira reaction of 4-iodo triazole **1z** gave product **6z** in low yield (Scheme 8).

**Scheme 8:**
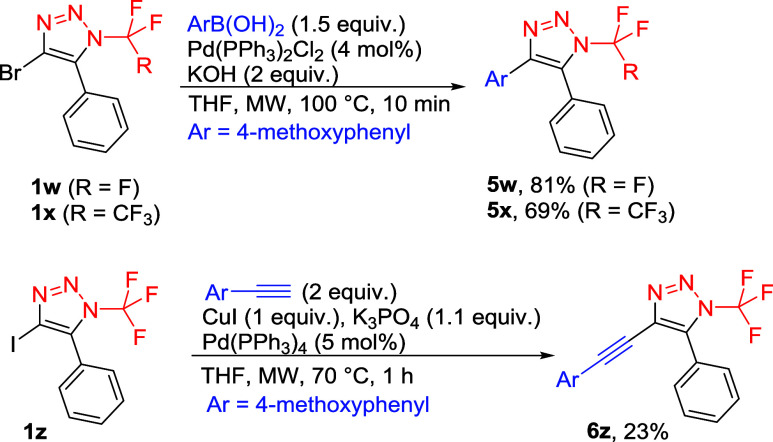
Cross-Coupling Reactions of 4-Halogenated 5-Phenyl-1,2,3-triazoles

## Conclusion

In conclusion, we present a mild and facile preparation of *N*-(per)­fluoroalkylated 5-substituted 1,2,3-triazoles based on the reaction of carbonyl-stabilized phosphonium ylides with fluorinated azides. The reaction is complementary to CuAAC, affording opposite regioisomers of the triazoles. The phosphonium ylide cyclization reaction displays a wide product scope and tolerates reactive functional groups. The employment of halogenated phosphonium ylides afforded 4-halogenated triazole derivatives. They undergo rhodium­(II)-catalyzed transannulation with benzonitrile to form new *N*-fluoroalkylated 4-halogenated imidazoles. The 4-halogenated triazoles were amenable to Pd-catalyzed cross-coupling reactions.

## Supplementary Material



## Data Availability

The data underlying this study are available in the published article and its Supporting Information.

## Notes

Experimental procedures, copies of NMR spectra, and details of X-ray analyses (PDF)
