# Supplementary material for: Synthesis of 1‑Fluoroalkyl-5-Substituted-1,2,3-Triazoles from Carbonyl-Stabilized Phosphonium Ylides
Source: J Org Chem. 2025 Aug 11;90(33):11838–43. doi: 10.1021/acs.joc.5c01055 (PMC12381930; doi:10.1021/acs.joc.5c01055)

Supplementary information for

Synthesis of 1-Fluoroalkyl-5-Substituted-1,2,3-Triazoles from Carbonyl-Stabilized Phosphonium Ylides

David Tichý,[†,a,b] Tomáš Černý,[†,a,c] Josef Filgas,[a,d] Svatava Voltrová,[a] Blanka Klepetářová,[a] and Petr Beier[*,a]

[a] Institute of Organic Chemistry and Biochemistry of the Czech Academy of Sciences, Flemingovo nám. 2, 160 00 Prague, Czechia.

[b] Charles University, Faculty of Science, Albertov 6, 128 00 Prague, Czechia.

[c] Institute of Organic Chemistry and Technology, University of Pardubice, Studentská 573, 532 10 Pardubice, Czechia.

[d] Department of Physical Chemistry, University of Chemistry and Technology, Technická 5, 166 28 Prague, Czechia.

[†] Both authors contributed equally.

[*] Corresponding author (Email: beier@uochb.cas.cz).

TABLE OF CONTENTS **page**

EXPERIMENTAL SECTION SI2
General Information SI2
General procedure A for the synthesis of triazoles 1 SI2
General procedure B for the synthesis of triazoles 1 SI2
General procedure C for the synthesis of triazoles 1 SI2
General procedure D (Suzuki coupling) for the synthesis of triazoles 5 SI2
X-ray crystallography SI17
REFERENCES SI17
COPIES OF NMR SPECTRA SI19

EXPERIMENTAL SECTION

General Information. Fluorinated azidoalkanes were synthesized according to published procedures.[1–3] Other chemicals were obtained from commercial sources and used as received. THF was freshly distilled over Na/benzophenone prior to use. ^{1}H, ^{13}C, and ^{19}F NMR spectra were measured at ambient temperature using 5 mm diameter NMR tubes. The chemical shift values (δ) are reported in ppm relative to internal Me_4Si (0 ppm for ^{1}H and ^{13}C NMR) or residual solvents and internal $CFCl_3$ (0 ppm for ^{19}F NMR). High resolution mass spectra (HRMS) were recorded on a Waters Micromass AutoSpec Ultima or Agilent 7890A GC coupled with Waters GCT Premier orthogonal acceleration time-of-flight detector using electron impact (EI) ionization, on an LTQ Orbitrap XL using electrospray ionization (ESI), and on a Bruker solariX 94 ESI/MALDI-FT-ICR using dual ESI/MALDI ionization.

General procedure A for the synthesis of triazoles 1

A 10 mL Schlenk flask was subjected to three cycles of evacuation and backfilling with nitrogen gas. Phenylacetylene (41 mg, 0.4 mmol, 1 equiv.) was added into the flask, followed by the addition of THF (2 ml, 0.2M). The reaction mixture was cooled to −78 °C, and a solution of *n*-BuLi (0.16 mL, 0.4 mmol, 2.5M in hexanes, 1 equiv.) was added dropwise. The mixture was stirred at −78 °C for 15 minutes. Subsequently, solution of azide in THF (0.48 mmol, 0.24 M, 1.2 equiv.) was added, and the reaction mixture was stirred for an additional 30 minutes. The reaction was quenched by the addition of a saturated NH_4Cl solution (15 ml). The crude product was extracted with EtOAc (30 ml) three times. The combined organic phase was dried over anhydrous sodium sulfate, filtered, and the solvent was removed under reduced pressure. The crude product was purified by flash chromatography (cyclohexane/EtOAc).

General procedure B for the synthesis of triazoles 1

Chloroketone (1 mmol, 1 equiv.) and Ph_3P (275.4 mg, 1.05 mmol, 1.05 equiv.) were suspended in a mixture of MeCN and water (1:1, 1 M) in a flask. The flask was closed with a septum and the reaction mixture was stirred in an oil bath at 70 °C for 3 h, then cooled down to room temperature and a THF solution of an appropriate azide (1.1 mmol, 0.25 M, 1.1 equiv.) was added. Sodium carbonate (55.6 mg, 0.525 mmol, 0.525 equiv.) was added, the flask was closed with a septum, and the mixture was stirred for 10 minutes at room temperature. The reaction mixture was evaporated on silica gel and the silica gel was transferred onto a frit. The crude product was washed out of the silica gel with Et_2O and solvent was removed under reduced pressure. Purification by flash chromatography (silica gel) was performed first by washing the column with EtOAc (30 ml) and then with cyclohexane (60 ml) prior to the separation (cyclohexane/EtOAc, 95:5) yielded pure triazole **1**.

General procedure C for the synthesis of triazoles 1

Chloroketone (1 mmol, 1 equiv.) and Ph_3P (275.4 mg, 1.05 mmol, 1.05 equiv.) were suspended in a mixture of MeCN and water (1:1, 1M) in a flask. The flask was closed with a septum and the reaction mixture was stirred in an oil bath at 70 °C for 24 h, then cooled down to room temperature and a THF solution of an appropriate azide (1.1 mmol, 0.25 M, 1,1 equiv.) was added. Sodium carbonate (55.6 mg, 0.525 mmol, 0.525 equiv.) was added, the flask was closed with a septum, and the mixture was stirred for 10 minutes at room temperature. The reaction mixture was evaporated on silica gel and the silica gel was transferred onto a frit. The crude product was washed out of the silica gel with Et_2O and the solvent was removed under reduced pressure. Purification by flash chromatography (silica gel) was performed first by washing the column with EtOAc (30 ml) and then with cyclohexane (60 ml) prior to the separation (cyclohexane/EtOAc, 95:5) yielded pure triazole **1**.

General procedure D (Suzuki coupling) for the synthesis of triazoles 5.

A 10 mL microwave reaction vessel was evacuated and backfilled with nitrogen three times. Subsequently triazole **1w** (58.4 mg, 0.2 mmol, 1 equiv.) or **1x** (68.4 mg, 0.2 mmol, 1 equiv.), 4-methoxyphenylboronic acid (45.6 mg, 0.3 mmol, 1.5 equiv.), $PdCl_2(PPh_3)_2$ (5.6 mg, 0.08 mmol, 4 mol%, 0.4 equiv.), and KOH (22.4 mg, 0.4 mmol, 2equiv.) were added to the flask. The reagents were dissolved in dry THF (3 ml, 0.067 M), and the reaction mixture was subjected to microwave irradiation (300 W, 10 minutes, 100 °C). The resulting mixture was filtered through celite, the filtrate was diluted with H_2O (15 ml) and extracted with EtOAc (30 ml). The organic phases were combined, dried over anhydrous Na_2SO_4 and filtered. Excess solvents were evaporated. Purification on automated column chromatography (silica gel) was performed first by washing the column with EtOAc (30 ml) and then with cyclohexane (60 ml) prior to the separation (silica gel, cyclohexane/EtOAc, 95:5) yielded pure triazole **5**.

Attempted synthesis of ***1i*** *using ruthenium catalysis:* A THF solution of pentafluoroazidoethane (1.2 mmol, 0.25 M, 1.2 equiv.) was added to the mixture of phenyl acetylene (102.1 mg, 1 mmol, 1 equiv.) and Cp*RuCl(cod) (18.9 mg, 0.05 mmol, 0.05 equiv.) at 0 °C under inert atmosphere. The reaction mixture was stirred at room temperature for 18 hours. No triazole **1i** was detected and only the starting material was present in the mixture.

5-Phenyl-1-(trifluoromethyl)-1H-1,2,3-triazole ***(1a)*** General procedures A and B were used. Purified by flash chromatography (cyclohexane/EtOAc, 95:5). Pale-yellow liquid. Yield (A) 44 mg (51 %), yield (B) 183.2 mg, 85%, ^{1}H NMR (400 MHz, $CDCl_3$) δ = 7.72 (m, 1H), 7.57–7.46 (m, 3H), 7.46–7.38 (m, 2H); ^{13}C {^{1}H} NMR (101 MHz, $CDCl_3$) δ = 138.3, 134.7 (q, $J_{C\text{-}F}$ = 1.7 Hz), 130.7, 129.2 (q, $J_{C\text{-}F}$ = 0.9 Hz), 129.0, 124.7, 119.8 (q, $J_{C\text{-}F}$ = 269.1 Hz); ^{19}F NMR (377 MHz, $CDCl_3$) δ = −55.6 (s); HRMS (ESI) *m/z* calcd for $C_9H_7N_3F_3$ $[M+H]^+$ 214.0586, found 214.0588.

5-(p-tolyl)-1-(trifluoromethyl)-1H-1,2,3-triazole ***(1b)*** General procedure A was used. Purified by flash chromatography (cyclohexane/EtOAc, 95:5). Pale yellow oil. Yield 56.6 mg, (62 %), ^{1}H NMR (400 MHz, $CDCl_3$) δ = 7.71–7.70 (m, 1H), 7.33–7.29 (m, 4H), 2.44 (s, 3H); ^{13}C {^{1}H} NMR (101 MHz, $CDCl_3$) δ = 141.0, 138.5, 134.6 (q, $J_{C\text{-}F}$ = 1.8 Hz), 129.7, 129.1, 121.7, 118.4 (q, $J_{C\text{-}F}$ = 269 Hz), 21.5;

^{19}F NMR (376 MHz, $CDCl_3$) δ = −55.7 (s); HRMS (EI) *m/z* calcd for $C_{10}H_9F_3N_3$ $[M]^+$ 227.0665, found 227.0665.

5-(2-methoxyphenyl)-1-(trifluoromethyl)-1H-1,2,3-triazole (**1c**) General procedure A was used. Purified by flash chromatography (cyclohexane/EtOAc, 95:5). Pale yellow oil. Yield 60.4 mg, (54 %), ^1H NMR (400 MHz, DMSO-d_6) δ = 8.09 (m, 1H), 7.6–7.55 (m, 1H), 7.39 (dd, *J* = 7.5 Hz, 1.8 Hz, 1H), 7.22–7.2 (m, 1H), 7.1 (td, *J* = 7.5, 1.0 Hz, 1H), 3.78 (s, 3H); ^{13}C {^1H} NMR (101 MHz, DMSO-d_6) δ = 156.9, 135.2 (q, J_{C-F} = 1.6 Hz), 134.9, 132.7, 131.1, 120.6, 117.9 (q, J_{C-F} = 268 Hz), 113.1, 111.5, 55.6; ^{19}F NMR (376 MHz, DMSO-d_6) δ = −59.4 (s); HRMS (EI) *m/z* calcd for $C_{10}H_8F_3N_3O$ $[M]^+$ 243.0614, found 243.0614.

5-(4-methoxyphenyl)-1-(trifluoromethyl)-1H-1,2,3-triazole (**1d**) General procedure A was used. Purified by flash chromatography (cyclohexane/EtOAc, 95:5). Yellow oil. Yield 50.6 mg, (52 %), ^1H NMR (400 MHz, $CDCl_3$) δ = 7.69–7.68 (m, 1H), 7.38–7.34 (m, 2H), 7.02–6.69 (m, 2H), 3.87 (s, 3H); ^{13}C {^1H} NMR (101 MHz, $CDCl_3$) δ = 161.4, 138.3, 134.5 (q, J_{C-F} = 1.7 Hz), 130.6, 118.4 (q, J_{C-F} = 269 Hz), 116.6, 114.5, 55.6; ^{19}F NMR (376 MHz, $CDCl_3$) δ = −55.7 (s); HRMS (ESI) *m/z* calcd for $C_{10}H_9F_3N_3O$ $[M + H]^+$ 244.0692, found 244.0694.

5-(4-(trifluoromethyl)phenyl)-1-(trifluoromethyl)-1H-1,2,3-triazole (**1e**) General procedure A was used. Purified by flash chromatography (cyclohexane/EtOAc, 95:5). Yellow oil. Yield 60.4 mg, (54 %), ^1H NMR (400 MHz, $CDCl_3$) δ = 7.80–7.77 (m, 3H), 7.60– 7.57 (m, 2H); ^{13}C {^1H} NMR (101 MHz, $CDCl_3$) δ = 136.8, 135.1 (q, J_{C-F} = 1.6 Hz), 132.9 (q, J_{C-F} = 33 Hz), 129.8, 128.5, 126.1 (q, J_{C-F} = 3.7 Hz), 123.6 (q, J_{C-F} = 273 Hz), 118.3 (q, J_{C-F} = 270 Hz); ^{19}F NMR (376 MHz, $CDCl_3$) δ = −55.4 (s, 3F), −63.6 (s, 3F); HRMS (EI) *m/z* calcd for $C_{10}H_5F_6N_3$ $[M]^+$ 281.0382, found 281.0376.

5-(p-tolyl)-1-(perfluoroethyl)-1H-1,2,3-triazole (**1f**) General procedures A and B were used. Purified by flash chromatography (cyclohexane/EtOAc, 95:5). White solid. **1f** should be stored in a refrigerator. Yield (A) 23.8 mg, (22 %), yield (B) 254.4 mg, 91%, ^1H NMR (400 MHz, $CDCl_3$) δ = 7.71 (s, 1H), 7.33–7.26 (m, 4H), 2.43 (s, 3H); ^{13}C {^1H} NMR (101 MHz, $CDCl_3$) δ = 140.9, 139.8 (t, J_{C-F} = 1.8 Hz), 134.8, 129.5, 129.4 (t, J_{C-F} = 1.6 Hz), 121.9, 117.2 (qt, J_{C-F} = 287.7, 39.6 Hz), 114.9 (tq, J_{C-F} = 269.8, 42.7 Hz), 21.44; ^{19}F NMR (376 MHz, $CDCl_3$) δ = −82.19 (s, 3F), −93.21 (s, 2F); HRMS (EI) *m/z* calcd for $C_{11}H_8F_5N_3$ $[M]^+$ 277.0638, found 277.0628.

1-(Difluoromethyl)-5-phenyl-1H-1,2,3-triazole (**1g**) 2-chloro-1-phenylethan-1-one (154.6 mg, 1 mmol, 1 equiv.) and Ph_3P (275.4 mg, 1.05 mmol, 1.05 equiv.) were suspended in a mixture of MeCN (0.5 ml) and water (0.5 ml) in a flask. The flask was closed with a septum and the reaction mixture was stirred in an oil bath at 70 °C for 3 h, then cooled down to room temperature and a THF solution of azidodifluoromethane (1.1 mmol, 1M, 1.1 equiv.) was added. Sodium carbonate (55.6 mg, 0.525 mmol, 0.525 equiv.) was added, the flask was closed with a septum, and the mixture was stirred for 2 h at room temperature. The reaction mixture was evaporated on silica gel and the silica gel was transferred onto a frit. The crude product was washed out of the silica gel with Et_2O and the solvent was removed under reduced pressure. Purification with flash chromatography (silica gel) was performed first by washing the column with EtOAc (30 ml) and then with cyclohexane (60 ml) prior to the separation (cyclohexane/EtOAc, 95:5) yielded the product as an oil which slowly solidified into a white low-melting solid. Yield 129.9 mg, 66%, ^1H NMR (400 MHz, $CDCl_3$) δ = 7.77 (d, J_{H-H} = 0.6 Hz, 1H), 7.58 (td, J_{H-F} = 58.2 Hz, J_{H-H} = 0.6 Hz, 1H), 7.56–7.45 (m, 5H); ^{13}C {^1H} NMR (101 MHz, $CDCl_3$) δ = 137.9, 134.5, 130.4, 129.1, 129.1 (t, *J* = 1.7 Hz, signal overlap), 125.0, 110.6 (t, J_{C-F} = 255.0 Hz); ^{19}F NMR (377 MHz, $CDCl_3$) δ = −94.5 (d, J_{F-H} = 58.3 Hz); HRMS (ESI) *m/z* calcd for $C_9H_8F_2N_3$ $[M+H]^+$ 196.0681, found 196.0682.

1-(2-Bromo-1,1,2,2-tetrafluoroethyl)-5-phenyl-1H-1,2,3-triazole (**1h**) General procedure B was used. Purified by flash chromatography (cyclohexane/EtOAc, 95:5). Oil which slowly solidified into a pale-yellow low-melting solid. Yield 291.9 mg, 90%, ^1H NMR (400 MHz, $CDCl_3$) δ = 7.70 (t, J_{H-F} = 0.7 Hz, 1H), 7.55–7.43 (m, 3H), 7.43–7.35 (m, 2H); ^{13}C {^1H} NMR (101 MHz, $CDCl_3$) δ = 139.9 (t, J_{C-F} = 1.5 Hz), 135.0, 130.4, 129.7 (t, J_{C-F} = 1.7 Hz), 128.7, 125.2, 113.9 (tt, J_{C-F} = 315.5, 43.6 Hz), 112.8 (tt, J_{C-F} = 271.8, 33.9 Hz); ^{19}F NMR (377 MHz, $CDCl_3$) δ = −65.24 (t, J_{F-F} = 4.8 Hz, 2F), −90.09 (t, J_{FF} = 4.6 Hz, 2F); HRMS (ESI) *m/z* calcd for $C_{10}H_7BrF_4N_3$ $[M+H]^+$ 323.9754, found 323.9756.

1-(Perfluoroethyl)-5-phenyl-1H-1,2,3-triazole (**1i**) General procedure B was used. Purified by flash chromatography (cyclohexane/EtOAc, 95:5). Oil which slowly solidified to a pale-yellow low-melting solid. Yield 231.7 mg, 90%, ^1H NMR (400 MHz, $CDCl_3$) δ = 7.73 (s, 1H), 7.56–7.45 (m, 3H), 7.45–7.39 (m, 2H); ^{13}C {^1H} NMR (101 MHz, $CDCl_3$) δ = 139.7 (t, J_{C-F} = 1.6 Hz), 134.9, 130.6, 129.5 (t, J_{C-F} = 1.6 Hz), 128.8, 124.9, 117.2 (qt, J_{C-F} = 287.8, 39.7 Hz), 111.4 (tq, J_{C-F} = 269.9, 42.8 Hz); ^{19}F NMR (377 MHz, $CDCl_3$) δ = −82.2 (s, 3F), −93.2 (s, 2F); HRMS (EI) *m/z* calcd for $C_{10}H_6F_5N_3$ $[M]^+$ 263.0482, found 263.0477.

5-(4-Methoxyphenyl)-1-(perfluoroethyl)-1H-1,2,3-triazole (**1j**) General procedure B was used. Purified by flash chromatography (cyclohexane/EtOAc, 95:5). Pale-yellow liquid. **1j** should be stored in a refrigerator. Yield 270.3 mg, 92%, ^1H NMR (400 MHz, $CDCl_3$) δ = 7.68 (t, J_{H-F} = 0.4 Hz, 1H), 7.39–7.29 (m, 2H), 7.03–6.94 (m, 2H), 3.85 (s, 3H); ^{13}C {^1H} NMR (101 MHz, $CDCl_3$) δ = 161.4, 139.7 (t, J_{C-F} = 1.7 Hz), 134.7, 130.9 (t, J_{C-F} = 1.7 Hz), 117.2 (qt, J_{C-F} = 287.7, 39.7 Hz), 116.7, 114.3, 111.4 (tq, J_{C-F} = 269.3, 42.7 Hz), 55.48; ^{19}F NMR (377 MHz, $CDCl_3$) δ = −82.1 (s, 3F), −93.2 (s, 2F); HRMS (ESI) *m/z* calcd for $C_{11}H_9F_5N_3O$ $[M+H]^+$ 294.0660, found 294.0662.

5-(2-Methoxyphenyl)-1-(perfluoroethyl)-1H-1,2,3-triazole (**1k**) General procedure B was used. Purified by flash chromatography (cyclohexane/EtOAc, 95:5). Pale-yellow liquid. Yield 263.7 mg, 89%, ^1H NMR (400 MHz, $CDCl_3$) δ = 7.67 (s, 1H), 7.56–7.40 (m, 1H), 7.25–7.18 (m, 1H), 7.01–6.92 (m, 2H), 3.77 (s, 3H); ^{13}C {^1H} NMR (101 MHz, $CDCl_3$) δ = 157.6, 136.1, 135.2, 132.4, 131.2, 120.5, 117.3 (qt, J_{C-F} = 288.0, 40.3 Hz), 114.1 (t, J_{C-F} = 1.3 Hz), 111.3 (tq, J_{C-F} = 270.6, 42.7 Hz), 110.9, 55.5; ^{19}F NMR (377 MHz, $CDCl_3$) δ = −82.7 (s, 3F), −97.3 (s, 2F); HRMS (ESI) *m/z* calcd for $C_{11}H_9F_5N_3O$ $[M]^+$ 294.0660, found 294.0661.

2-(1-(Perfluoroethyl)-1H-1,2,3-triazol-5-yl)phenol (**1l**) General procedure B was used. Purified by flash chromatography (cyclohexane/EtOAc, 95:5). White solid. Yield 205.1 mg, 73%, ^1H NMR (400 MHz, $CDCl_3$) δ = 7.77 (s, 1H), 7.46–7.34 (m, 1H), 7.28–7.16 (m, 1H, solvent signal overlap), 7.10–6.99 (m, 1H), 6.99–6.92 (m, 1H), 5.76 (brs, 1H); ^{13}C {^1H} NMR (101 MHz, $CDCl_3$) δ = 154.2, 135.6, 135.5 (signal overlap), 132.4, 131.5, 120.8, 117.3 (qt, J_{C-F} = 287.9, 40.0 Hz, signal overlap), 116.1, 112.0, 111.3 (tq, J_{C-F} = 271.1, 42.8 Hz); ^{19}F NMR (377 MHz, $CDCl_3$) δ = −82.7 (s, 3F), −96.4 (s, 2F);

HRMS (ESI) *m/z* calcd for $C_{10}H_5F_5N_3O$ [M–H]$^-$ 278.0358, found 278.0356.

5-(4-Fluorophenyl)-1-(perfluoroethyl)-1H-1,2,3-triazole (**1m**) General procedure B was used. Purified by flash chromatography (cyclohexane/EtOAc, 95:5). Oil which slowly solidified into a pale-yellow solid. Yield 254.2 mg, 90%, ^{1}H NMR (400 MHz, $CDCl_3$) δ = 7.72 (s, 1H), 7.46–7.37 (m, 2H), 7.22–7.14 (m, 2H); ^{13}C {^{1}H} NMR (101 MHz, $CDCl_3$) δ = 164.1 (d, J_{C-F} = 251.9 Hz), 138.7 (t, J_{C-F} = 1.6 Hz), 135.1, 131.6 (dt, J_{C-F} = 8.7, 1.7 Hz), 120.9 (dt, J_{C-F} = 3.6, 1.1 Hz), 117.2 (qt, J_{C-F} = 287.8, 39.5 Hz), 116.2 (d, J_{C-F} = 22.1 Hz), 111.4 (tq, J_{C-F} = 270.1, 42.8 Hz); ^{19}F NMR (377 MHz, $CDCl_3$) δ = –82.3 (s, 3F), –93.2 (s, 2F), –109.73 (m, 1F); HRMS (EI) *m/z* calcd for $C_{10}H_5F_6N_3$ [M]$^+$ 281.0388, found 281.0385.

5-(4-Chlorophenyl)-1-(perfluoroethyl)-1H-1,2,3-triazole (**1n**) General procedure B was used. Purified by flash chromatography (cyclohexane/EtOAc, 95:5). White solid. Yield 271.7 mg, 91%, ^{1}H NMR (400 MHz, $CDCl_3$) δ = 7.73 (s, 1H), 7.50–7.44 (m, 2H), 7.41–7.33 (m, 2H); ^{13}C {^{1}H} NMR (101 MHz, $CDCl_3$) δ = 138.5 (t, J_{C-F} = 1.6 Hz), 137.1, 135.1, 130.8 (t, J_{C-F} = 1.7 Hz), 129.2, 123.3, 117.1 (qt, J_{C-F} = 287.7, 39.5 Hz), 111.4 (tq, J_{C-F} = 269.8, 43.0 Hz); ^{19}F NMR (377 MHz, $CDCl_3$) δ = –82.3 (s, 3F), –93.2 (s, 2F); HRMS (EI) *m/z* calcd for $C_{10}H_5ClF_5N_3$ [M]$^+$ 297.0092, found 297.0095.

5-(4-Bromophenyl)-1-(perfluoroethyl)-1H-1,2,3-triazole (**1o**) General procedure B was used. Purified by flash chromatography (cyclohexane/EtOAc, 95:5). White solid. Yield 255.9 mg, 74%, ^{1}H NMR (400 MHz, $CDCl_3$) δ = 7.74 (s, 1H), 7.69–7.55 (m, 2H), 7.39–7.26 (m, 2H, solvent signal overlap); ^{13}C {^{1}H} NMR (101 MHz, $CDCl_3$) δ = 138.5 (t, J_{C-F} = 1.7 Hz), 135.0, 132.2, 131.0 (t, J_{C-F} = 1.7 Hz), 125.4, 123.8, 117.1 (qt, J_{C-F} = 287.6, 39.5 Hz), 111.4 (tq, J_{C-F} = 270.2, 42.9 Hz); ^{19}F NMR (377 MHz, $CDCl_3$) δ = –82.3 (s, 3F), –93.2 (s, 2F); HRMS (ESI) *m/z* calcd for $C_{10}H_6BrF_5N_3$ [M+H]$^+$ 341.9660, found 341.9660.

5-(4-Iodophenyl)-1-(perfluoroethyl)-1H-1,2,3-triazole (**1p**) 2-bromo-1-(4-iodophenyl)ethan-1-one (324.9 mg, 1 mmol, 1 equiv.) and Ph_3P (275.4 mg, 1.05 mmol) were suspended in a mixture of MeCN (0.5 ml) and water (0.5 ml) in a flask. The flask was closed with a septum and the reaction mixture was stirred in an oil bath at 70 °C for 2 h. The reaction mixture was allowed to cool down to room temperature and a THF solution of azidopentafluoroethane (1.1 mmol, 0.25 M, 1.1 equiv.) was added. Sodium carbonate (55.6 mg, 0.525 mmol, 0.525 equiv.) was added, the flask was closed with a septum, and the mixture was stirred for 10 minutes at room temperature. The reaction mixture was evaporated on silica gel and the silica gel was transferred onto a frit. The crude product was washed out of the silica gel with Et_2O and the solvent was removed under reduced pressure. Purification with flash chromatography (silica gel) was performed first by washing the column with EtOAc (30 ml) and then with cyclohexane (60 ml) prior to the separation (cyclohexane/EtOAc, 95:5) yielded the product as a white solid. Yield 287.7 mg, 73%, ^{1}H NMR (400 MHz, $CDCl_3$) δ = 7.95–7.78 (m, 2H), 7.73 (s, 1H), 7.25–7.05 (m, 2H); ^{13}C {^{1}H} NMR (101 MHz, $CDCl_3$) δ = 138.6 (t, J_{C-F} = 1.5 Hz), 138.1, 135.0, 131.0 (t, J_{C-F} = 1.7 Hz), 124.4, 117.1 (qt, J_{C-F} = 287.8, 39.5 Hz), 111.4 (tq, J_{C-F} = 270.0, 42.9 Hz), 97.2; ^{19}F NMR (377 MHz, $CDCl_3$) δ = –82.3 (s, 3F), –93.2 (s, 2F); HRMS (ESI) *m/z* calcd for $C_{10}H_5F_5IN_3$ [M]$^+$ 389.9521, found 389.9519.

5-(4-Nitrophenyl)-1-(perfluoroethyl)-1H-1,2,3-triazole (**1q**) General procedure B was used. Purified by flash chromatography (cyclohexane/EtOAc, 95:5). Pale-yellow solid. Yield 290.0 mg, 94%, ^{1}H NMR (400 MHz, $CDCl_3$) δ = 8.40–8.29 (m, 2H), 7.84 (s, 1H), 7.71–7.60 (m, 2H); ^{13}C {^{1}H} NMR (101 MHz, $CDCl_3$) δ = 149.2, 137.4 (t, J_{C-F} = 1.7 Hz), 135.4, 131.3, 130.8 (t, J_{C-F} = Hz), 124.0, 117.0 (qt, J_{C-F} = 287.6, 39.4 Hz), 111.4 (tq, J_{C-F} = 270.7, 43.1 Hz); ^{19}F NMR (377 MHz, $CDCl_3$) δ = –82.3 (s, 3F), –93.0 (s, 2F); HRMS (ESI) *m/z* calcd for $C_{10}H_4F_5N_4O_2$ [M–H]$^-$ 307.0260, found 307.0259.

5-Benzyl-1-(perfluoroethyl)-1H-1,2,3-triazole (**1r**) General procedure C was used. Purified by flash chromatography (cyclohexane/EtOAc, 95:5). Yellow liquid. Yield 216.8 mg, 78%, ^{1}H NMR (400 MHz, $CDCl_3$) δ = 7.41–7.30 (m, 3H), 7.28 (tq, J_{C-F} = 0.9, 0.4 Hz, 1H), 7.25–7.15 (m, 2H), 4.14 (s, 2H); ^{13}C {^{1}H} NMR (101 MHz, $CDCl_3$) δ = 139.4 (t, J_{C-F} = 2.3 Hz), 135.0, 134.9, 129.2, 128.8, 127.8, 117.3 (qt, J_{C-F} = 287.6, 40.3 Hz), 111.5 (tq, J_{C-F} =268.9, 42.9 Hz), 29.9 (t, J_{C-F} = 3.8 Hz); ^{19}F NMR (377 MHz, $CDCl_3$) δ = –82.8 (s, 3F), –96.5 (s, 2F); HRMS (ESI) *m/z* calcd for $C_{11}H_9F_5N_3$ [M+H]$^+$ 278.0711, found 278.0713.

1-(Perfluoroethyl)-5-(thiophen-2-yl)-1H-1,2,3-triazole (**1s**) General procedure C was used. Purified by flash chromatography (cyclohexane/EtOAc, 95:5). Oil which slowly solidified into a pale-yellow solid. **1s** should be stored in a refrigerator. Yield 171.1 mg, 63%, ^{1}H NMR (400 MHz, $CDCl_3$) δ = 7.80 (s, 1H), 7.62–7.51 (m, 1H), 7.39–7.30 (m, 1H), 7.21–7.12 (m, 1H); ^{13}C {^{1}H} NMR (101 MHz, $CDCl_3$) δ = 135.4, 133.1 (t, J_{C-F} = 1.7 Hz), 130.8 (t, J_{C-F} = 2.9 Hz), 129.5, 128.2, 123.6, 117.2 (qt, J_{C-F} = 287.7, 39.0 Hz), 111.4 (tq, J_{C-F} = 269.7, 42.6 Hz); ^{19}F NMR (377 MHz, $CDCl_3$) δ = –81.8 (s, 3F), –93.3 (s, 2F); HRMS (ESI) *m/z* calcd for $C_8H_5F_5N_3S$ [M+H]$^+$ 270.0120, found 270.0120.

4,4'-Bis(1-(perfluoroethyl)-1H-1,2,3-triazol-5-yl)-1,1'-biphenyl (**1t**)
4,4'-Bis-chloracetyl-biphenyl (307.2 mg, 1 mmol, 1 equiv.) and Ph_3P (550.8 mg, 2.1 mmol, 2.1 equiv.) were suspended in a mixture of MeCN (1 ml) and water (1 ml) in a flask. The flask was closed with a septum and the reaction mixture was stirred in an oil bath at 70 °C for 24 h, then cooled down to room temperature and a THF solution of azidopentafluoroethane (1.1 mmol, 0.25 M, 1.1 equiv.) was added. Sodium carbonate (111.3 mg, 1.05 mmol, 1.05 equiv.) was added, the flask was closed with a septum, and the mixture was stirred for 10 minutes at room temperature. The reaction mixture was evaporated on silica gel and the silica gel was transferred onto a frit. The crude product was washed out of the silica gel with Et_2O and the solvent was removed under reduced pressure. Purification with flash chromatography (silica gel) was performed first by washing the column with EtOAc (30 ml) and then with cyclohexane (60 ml) prior to the separation (cyclohexane/EtOAc, 95:5) yielded the product as a pale-yellow solid. Yield 158.0 mg, 57%, ^{1}H NMR (400 MHz, $CDCl_3$) δ = 7.85–7.70 (m, 6H), 7.65–7.51 (m, 4H); ^{13}C {^{1}H} NMR (101 MHz, $CDCl_3$) δ = 142.0, 139.2 (t, J_{C-F} = 1.6 Hz), 135.0, 130.2 (t, J_{C-F} = 1.7 Hz), 127.6, 124.6, 117.2 (qt, J_{C-F} = 287.7, 39.5 Hz), 111.5 (tq, J_{C-F} = 269.9, 42.7 Hz); ^{19}F NMR (377 MHz, $CDCl_3$) δ = –82.1 (s, 3F), –93.0 (s, 2F); HRMS (ESI) *m/z* calcd for $C_{20}H_{11}F_{10}N_6$ [M+H]$^+$ 525.0880, found 525.0879.

4-Chloro-1-(perfluoroethyl)-5-phenyl-1H-1,2,3-triazole (**1v**)
2-Chloro-1-phenylethan-1-one (154.6 mg, 1 mmol, 1 equiv.) and Ph_3P (275.4 mg, 1.05 mmol, 1.05 equiv.) were suspended in a mixture of MeCN (2 ml) and water (0.04 ml) in a flask. The flask was closed with a septum and the reaction mixture was stirred in an oil

bath at 70 °C for 3 h, then cooled down to room temperature, NCS (140.2 mg, 1.05 mmol, 1.05 equiv.) was added, and the mixture was stirred at room temperature for 5 minutes. Triethylamine (0.146 ml, 1.05 mmol, 1.05equiv.) was added, turning the mixture yellow. THF solution of azidopentafluoroethane (1.1 mmol, 0.25 M, 1.1 equiv.) was added, the flask was closed with a septum, and the mixture was stirred for 1 hour. The reaction mixture was evaporated on silica gel and the silica gel was transferred onto a frit. The crude product was washed out of the silica gel with Et_2O and the solvent was removed under reduced pressure. Purification with flash chromatography (silica gel) was performed first by washing the column with EtOAc (30 ml) and then with cyclohexane (60 ml) prior to the separation (cyclohexane) yielded the product as a pale-yellow liquid. Yield 199.8 mg, 67%, ^{1}H NMR (400 MHz, $CDCl_3$) δ = 7.64–7.48 (m, 3H), 7.48–7.35 (m, 2H); ^{13}C {^{1}H} NMR (101 MHz, $CDCl_3$) δ = 136.8, 134.9, 131.1, 129.9 (t, J_{C-F} = 1.5 Hz), 129.0, 123.1, 117.0 (qt, J_{C-F} = 288.0, 39.3 Hz), 111.1 (tq, J_{C-F} = 271.9, 42.9 Hz); ^{19}F NMR (377 MHz, $CDCl_3$) δ = –82.3 (s, 3F), –94.2 (s, 2F); HRMS (EI) *m/z* calcd for $C_{10}H_5ClF_5N_3$ $[M]^+$ 297.0092, found 297.0086.

4-Bromo-5-phenyl-1-(trifluoromethyl)-1H-1,2,3-triazole ***(1w)*** To a 25 ml flask triphenylphosphine-(brombenzoyl-methylen) **3** (689 mg, 1.5 mmol, 1equiv.) was added and dissolved in DCM (3 ml). The reaction solution was placed in an ice bath and cooled to 0 °C. Then the solution of azidotrifluoromethane in THF (7.5 ml, 0.24M, 1.8 mmol, 1.2 equiv.) was added and reaction mixture was stirred overnight. Afterwards the reaction was quenched with water (15 ml) and crude product was extracted with DCM (30 ml). The organic phases were combined, dried over anhydrous sodium sulfate and filtered. Excess solvents were evaporated under reduced pressure. Purification with flash chromatography (silica gel) was performed first by washing the column with EtOAc (30 ml) and then with cyclohexane (60 ml) prior to the separation (silica gel, cyclohexane/EtOAc, 95:5) yielded pure triazole as transparent oil which slowly solidified into white solid compound. Yield 354 mg, 81 %, ^{1}H NMR (400 MHz, $CDCl_3$) δ = 7.61–7.52 (m, 3H), 7.43–7.41 (m, 2H); ^{13}C {^{1}H} NMR (101 MHz, CDCl3) δ = 136.0, 134.6, 131.2, 129.7, 129.1, 123.4, 117.7 (q, J_{C-F} = 270.8 Hz); ^{19}F NMR (376 MHz, $CDCl_3$) δ = –56.5 (s); HRMS (EI) *m/z* calcd for $C_9H_5BrF_3N_3$ $[M]^+$ 290.9613, found 290.9606.

4-Bromo-1-(perfluoroethyl)-5-phenyl-1H-1,2,3-triazole ***(1x)***
2-Chloro-1-phenylethan-1-one (154.6 mg, 1 mmol, 1 equiv.) and Ph_3P (275.4 mg, 1.05 mmol, 1.05 equiv.) were suspended in a mixture of MeCN (0.5 ml) and water (0.5 ml) in a flask. The flask was closed with a septum and the reaction mixture was stirred in an oil bath at 70 °C for 3 h, then cooled down to room temperature. In another flask, NBS (186.9 mg, 1.05 mmol, 1.05 equiv.) and KBr (130.9 mg, 1.1 mmol, 1.1 equiv.) were dissolved in a mixture of MeCN (1 ml) and water (1 ml). This solution was added dropwise into the reaction mixture. After 5 minutes of stirring a white precipitate appeared. The solvents were evaporated under reduced pressure and the residuum was suspended in DCM (1 ml). Methanol (0.2 ml) and saturated sodium carbonate solution (0.5 ml) were added. THF solution of azidopentafluoroethane (1.1 mmol, 0.25 M) was added, the flask was closed with a septum, and the mixture was stirred for 1 hour. The reaction mixture was evaporated on silica gel and the silica gel was transferred onto a frit. The crude product was washed out of the silica gel with Et_2O and the solvent was removed under reduced pressure. Purification with flash chromatography (silica gel) was performed first by washing the column with EtOAc (30 ml) and then with cyclohexane (60 ml) prior to the separation (cyclohexane) yielded the product as an oil which slowly solidified into a white solid. Yield 214.5 mg, 62%, ^{1}H NMR (400 MHz, $CDCl_3$) δ = 7.62–7.47 (m, 3H), 7.47–7.31 (m, 2H); ^{13}C {^{1}H} NMR (101 MHz, $CDCl_3$) δ = 137.4 (t, J_{C-F} = 1.6 Hz), 131.1, 130.0 (t, J_{C-F} = 1.4 Hz), 129.0, 123.9, 123.6 (t, J_{C-F} = 1.3 Hz), 117.0 (qt, J = 288.0, 39.4 Hz), 111.0 (tq, J_{C-F} = 271.8, 43.0 Hz); ^{19}F NMR (377 MHz, $CDCl_3$) δ = –82.3 (s, 3F), –94.2 (s, 2F); HRMS (EI) *m/z* calcd for $C_{10}H_5BrF_5N_3$ $[M]^+$ 340.9587, found 340.9585.

4-Iodo-1-(perfluoroethyl)-5-phenyl-1H-1,2,3-triazole ***(1y)***
From iodinated ylide ***3****:* 2-Chloro-1-phenylethan-1-one (154.6 mg, 1 mmol, 1 equiv.) and Ph_3P (275.4 mg, 1.05 mmol, 1.05 equiv.) were suspended in a mixture of MeCN (0.5 ml) and water (0.5 ml) in a flask. The flask was closed with a septum and the reaction mixture was stirred in an oil bath at 70 °C for 3 h, then cooled down to room temperature. In another flask, succinimide (104 mg, 1.05 mmol, 1.05 equiv.) and NaOH (42 mg, 1.05 mmol. 1.05 equiv.) were dissolved in a mixture of MeCN (1 ml) and water (1 ml), followed up by iodine (266.5 mg, 1.05 mmol, 1.05 equiv.). The iodinating reagent solution was added dropwise into the reaction mixture. After 5 minutes of stirring, a yellow precipitate appeared. The solvents were evaporated on a rotavap and the residuum was suspended in DCM (1 ml). Methanol (0.2 ml) and saturated sodium carbonate solution (0.5 ml) were added. THF solution of azidopentafluoroethane (1.1 mmol, 0.25 M, 1.1 equiv.) was added and the reaction mixture slowly turned black brown. The flask was closed with a septum, and the mixture was stirred for 1 h. The reaction mixture was evaporated on silica gel and the silica gel was transferred onto a frit. The crude product was washed out of the silica gel with Et_2O and the solvent was removed under reduced pressure. Purification with flash chromatography (silica gel) was performed first by washing the column with EtOAc (30 ml) and then with cyclohexane (60 ml) prior to the separation (cyclohexane) yielded the product as an oil which slowly solidified into a yellow solid. Yield 33.7 mg, 8%.

From lithiated triazole ***1i****:* An oven-dried flask with a stirring bar was charged with triazole **1i** (263 mg, 1 mmol, 1 equiv.). The flask was closed with a septum, evacuated and back-filled with nitrogen three times. Dry THF (2 ml) was added and the solution was cooled down to –78 °C. A solution of *n*-BuLi (1.1 mmol, 0.42 ml, 2.5 M in hexanes, 1.1 equiv.) was carefully added dropwise over 1 minute, turning the reaction mixture dark red. The reaction mixture was stirred at –78 °C for 5 minutes. A solution of iodine (279.2 mg, 1.1 mmol, 1.1 equiv.) in dry THF (2 ml) was added dropwise over 3 minutes and the reaction mixture was stirred at –78 °C for 5 minutes. The flask was removed from the cooling bath and the reaction mixture was immediately diluted with pentane (20 ml). The mixture was allowed to warm up to room temperature and silica gel was added. The solvents were evaporated under reduced pressure. Purification on automated column chromatography (silica gel) was performed first by washing the column with EtOAc (30 ml) and then with cyclohexane (60 ml) prior to the separation (cyclohexane) yielded the product as an oil which slowly solidifies into a yellow solid. Yield 311.2 mg, 81%, ^{1}H NMR (400 MHz, $CDCl_3$) δ = 7.64–7.45 (m, 3H), 7.45–7.29 (m, 2H); ^{13}C {^{1}H} NMR (101 MHz, $CDCl_3$) δ = 141.4 (t, J_{C-F} = 1.7 Hz), 131.0, 130.1 (t, J_{C-F} = 1.4 Hz), 129.0, 124.5 (t, J_{C-F} = 1.3 Hz), 117.0 (qt, J_{C-F} = 287.9, 39.4 Hz), 110.8 (tq, J_{C-F} = 272.1, 43.0 Hz), 94.0; ^{19}F

NMR (377 MHz, $CDCl_3$) δ = –82.4 (s, 3F), –94.1 (s, 2F); HRMS (ESI) *m/z* calcd for $C_{10}H_6F_5IN_3$ $[M+H]^+$ 389.9521, found 389.9517.

*4-Iodo-5-phenyl-1-(trifluoromethyl)-1H-1,2,3-triazole (**1z**)*

*From lithiated triazole **1a**:* A 50 mL Schlenk flask was evacuated and backfilled with nitrogen three times. Subsequently triazole **1a** (264 mg, 1.24 mmol, 1.24 equiv.) and dry THF (3 ml) were added and the reaction mixture was cooled to –78 °C while stirring. A solution of *n*-BuLi (1.362 mmol, 0.545 ml, 2.5 M in hexane, 1.1 equiv.) was then added dropwise, resulting in a visible color change from colorless to yellow, and finally to dark red. After the addition was complete, the reaction was stirred for an additional 5 minutes at –78 °C. In a separate flask, iodine (330 mg, 1.3 mmol, 1.05 equiv.) was dissolved in THF (3 ml) under N_2 atmosphere. The iodine solution was carefully added to the reaction mixture over a period of 3 minutes. The reaction was stirred for further 5 minutes at –78 °C. The reaction flask was removed from the cooling bath and the mixture was diluted with pentane (30 ml). Silica gel was added to the mixture, and the solvent was evaporated. Purification on automated column chromatography (silica gel) was performed first by washing the column with EtOAc (30 ml) and then with cyclohexane (60 ml) prior to the separation (silica gel, cyclohexane/EtOAc, 95:5) yielded pure triazole as white solid compound. Yield 366 mg, 87%, ^{1}H NMR (400 MHz, $CDCl_3$) δ = 7.61–7.52 (m, 3H), 7.4–7.37 (m, 2H); ^{13}C {^{1}H} NMR (101 MHz, $CDCl_3$) δ = 140.1, 131.1, 129.9, 129.1, 124.3, 117.5 (q, $J_{C\text{-}F}$ = 270.2 Hz), 93.4; ^{19}F NMR (376 MHz, $CDCl_3$) δ = –56.4 (s); HRMS (ESI) *m/z* calcd for $C_9H_6F_3IN_3$ $[M+H]^+$ 339.9553, found 339.9555.

*4-Chloro-1-(perfluoroethyl)-2,5-diphenyl-1H-imidazole (**4v**)*

A microwave reactor tube was charged with a stirring bar, triazole **1v** (74.4 mg, 0.25 mmol, 1 equiv.), benzonitrile (28.4 mg, 0.275 mmol, 1.1 equiv.), $Rh_2(Oct)_4$ (5.8 mg, 7.5 µmol, 3 mol%, 0.03 equiv.), and chloroform (0.2 ml). The tube was closed with a cap and inserted into the microwave reactor. The reaction mixture was heated for 30 minutes at 170 °C. After the reaction mixture cooled down to room temperature, the content of the tube was transferred into a flask. Silica gel was added and the solvents were removed under reduced pressure. Purification on automated column chromatography (silica gel) was performed first by washing the column with EtOAc (30 ml) and then with cyclohexane (60 ml) prior to the separation (cyclohexane/EtOAc, 9:1) afforded orange residuum which was carefully triturated with cold pentane to yield the product as a pale-yellow solid. Yield 45.5 mg, 48%, ^{1}H NMR (400 MHz, $CDCl_3$) δ = 8.27–6.89 (m, 10H, solvent signal overlap); ^{13}C {^{1}H} NMR (101 MHz, $CDCl_3$) δ = 148.4, 132.1, 131.4, 130.3 (signal overlap), 130.3 (t, $J_{C\text{-}F}$ = 2.1 Hz, signal overlap), 130.2, 129.7, 128.3, 128.1, 127.3, 117.3 (qt, $J_{C\text{-}F}$ = 289.4, 45.0 Hz), 110.8 (tq, $J_{C\text{-}F}$ = 272.3, 43.1 Hz); ^{19}F NMR (377 MHz, $CDCl_3$) δ = –84.3 (s, 3F), –91.1 (s, 2F); HRMS (ESI) *m/z* calcd for $C_{17}H_{11}ClF_5N_2$ $[M+H]^+$ 373.0525, found 373.0527.

*4-Bromo-1-(perfluoroethyl)-2,5-diphenyl-1H-imidazole (**4x**)*

A microwave reactor tube was charged with a stirring bar, triazole **1x** (85.5 mg, 0.25 mmol, 1 equiv.), benzonitrile (28.4 mg, 0.275 mmol, 1.1 equiv.), $Rh_2(Oct)_4$ (3.9 mg, 5 µmol, 2 mol%, 0.02 equiv.), and chloroform (0.2 ml). The tube was closed with a cap and inserted into the microwave reactor. The reaction mixture was heated for 20 minutes at 150 °C. After the reaction mixture cooled down to room temperature, the content of the tube was transferred into a flask. Silica gel was added and the solvents were removed under reduced pressure. Purification on automated column chromatography (silica gel) was performed first by washing the column with EtOAc (30 ml) and then with cyclohexane (60 ml) prior to the separation (cyclohexane/EtOAc, 9:1) afforded orange residuum which was carefully triturated with cold pentane to yield the product as a pale-yellow solid. Yield 69.3 mg, 66%, ^{1}H NMR (400 MHz, $CDCl_3$) δ = 7.83–7.20 (m, 10H, solvent signal overlap); ^{13}C {^{1}H} NMR (101 MHz, $CDCl_3$) δ = 149.4, 131.5 (br s), 130.7 (br s), 130.3, 130.2, 129.8, 128.2, 128.0, 127.8, 120.1, 117.2 (qt, $J_{C\text{-}F}$ = 289.3, 45.0 Hz), 110.7 (tq, $J_{C\text{-}F}$ = 272.2, 43.2 Hz); ^{19}F NMR (377 MHz, $CDCl_3$) δ = –84.3 (s, 3F), –91.0 (s, 2F); HRMS (ESI) *m/z* calcd for $C_{17}H_{11}BrF_5N_2$ $[M+H]^+$ 417.0020, found 417.0017.

*4-Iodo-1-(perfluoroethyl)-2,5-diphenyl-1H-imidazole (**4y**)*

A microwave reactor tube was charged with a stirring bar, triazole **1y** (97.3 mg, 0.25 mmol, 1 equiv.), benzonitrile (28.4 mg, 0.275 mmol, 1.1 equiv.), $Rh_2(Oct)_4$ (1.9 mg, 2.5 µmol, 1 mol%, 0.01 equiv.), and chloroform (0.2 ml). The tube was closed with a cap and inserted into the microwave reactor. The reaction mixture was heated for 20 minutes at 130 °C. After the reaction mixture cooled down to room temperature, the content of the tube was transferred into a flask. Silica gel was added and the solvents were removed under reduced pressure. Purification on automated column chromatography (silica gel) was performed first by washing the column with EtOAc (30 ml) and then with cyclohexane (60 ml) prior to the separation (cyclohexane/EtOAc, 9:1) afforded orange residuum which was carefully triturated with cold pentane to yield the product as a pale-yellow solid. Yield 68.8 mg, 59%, ^{1}H NMR (400 MHz, $CDCl_3$) δ = 7.84–7.17 (m, 10H, solvent signal overlap); ^{13}C {^{1}H} NMR (101 MHz, $CDCl_3$) δ = 151.1, 135.5, 131.7, 130.3 (t, $J_{C\text{-}F}$ = 2.1 Hz), 130.3, 130.2, 130.2, 129.8, 129.0 (t, $J_{C\text{-}F}$ = 1.8 Hz), 128.3, 128.0, 117.3 (qt, $J_{C\text{-}F}$ = 289.3, 44.9 Hz), 110.5 (tq, $J_{C\text{-}F}$ = 272.8, 43.2 Hz), 91.2; ^{19}F NMR (377 MHz, $CDCl_3$) δ = –84.2 (s, 3F), –90.9 (s, 2F); HRMS (ESI) *m/z* calcd for $C_{17}H_{11}F_5IN_2$ $[M+H]^+$ 464.9882, found 464.9879.

*4-(4-methoxyphenyl)-5-phenyl-1-(trifluoromethyl)-1H-1,2,3-triazole (**5w**)* General procedure **D** was used. Purified by flash chromatography (cyclohexane/EtOAc, 95:5). Pale white solid. Yield (51.5 mg, 81%), ^{1}H NMR (400 MHz, $CDCl_3$) δ = 7.58–7.49 (m, 3H), 7.47– 7.43 (m, 2H), 7.39–7.37 (m, 2H), 6.84–6.8 (m, 2H), 3.78 (s, 3H); ^{13}C {^{1}H} NMR (101 MHz, $CDCl_3$) δ = 160.0, 146.0, 132.0, 130.6, 130.2, 129.3, 128.6, 125.7, 121.7, 118.1 (q, $J_{C\text{-}F}$ = 269.2 Hz), 114.2, 55.4; ^{19}F NMR (376 MHz, $CDCl_3$): δ = –56.0 (s). HRMS (ESI) *m/z* calcd for $C_{16}H_{13}F_3N_3O$ $[M+H]^+$ 320.1005, found 320.1004.

*4-(4-methoxyphenyl)-1-(perfluoroethyl)-5-phenyl-1H-1,2,3-triazole (**5x**)* General procedure **D** was used. Purified by flash chromatography (cyclohexane/EtOAc, 95:5). White solid. Yield (51.2 mg, 69%), ^{1}H NMR (400 MHz, $CDCl_3$) δ = 7.57–7.48 (m, 3H), 7.45–7.41 (m, 2H), 7.39–7.37 (m, 2H), 6.83–6.79 (m, 2H), 3.78 (s, 3H); ^{13}C {^{1}H} NMR (126 MHz, $CDCl_3$) δ = 160.0, 146.2, 133.2, 130.5, 130.4, 129.2, 128.7, 126.0, 121.6, 117.2 (qt, $J_{C\text{-}F}$ = 287.9, 39.6 Hz), 114.2, 111.3 (tq, $J_{C\text{-}F}$ = 269.9, 42.7 Hz), 55.4; ^{19}F NMR (376 MHz, $CDCl_3$) δ = –82.7 (s, 3F), –93.47 (s, 2F); HRMS (ESI) *m/z* calcd for $C_{17}H_{13}F_5N_3O$ $[M+H]^+$ 370.0971, found 370.0972.

*4-((4-methoxyphenyl)ethynyl)-5-phenyl-1-(trifluoromethyl)-1H-1,2,3-triazole (**6z**)* A 10 ml microwave vessel, pre-dried in an oven, was loaded with triazole **1z** (68 mg, 0.2 mmol, 1 equiv.), CuI (38.1 mg, 0.2 mmol, 1 equiv.), K_3PO_4 (46.7 mg, 0.22 mmol, 1.1 equiv.), flask was evacuated and then backfilled with nitrogen three times and subsequently charged with $Pd(PPh_3)_4$ (11.6 mg, 0.01 mmol, 5

mol%, 0.05 equiv.). Afterwards a solution of 4-methoxyphenylacetylene (53 mg, 0.4 mmol, 2 equiv.) in dry THF (2.7 ml) was added and the reaction mixture was irradiated in microwave (300 W, 1 h, 70 °C). The reaction was quenched with H_2O (5 ml) and the crude product was extracted with EtOAc (30 ml). The organic phase was dried over anhydrous sodium sulfate and filtered. Excess solvents were evaporated and the crude product was purified with column chromatography (silica gel, DCM/toluene, 1:25) yielded 16 mg (23%) of the desired triazole **6z** as pale-yellow solid. ^{1}H NMR (400 MHz, $CDCl_3$) δ = 7.59–7.52 (m, 5H), 7.40–7.36 (m, 2H), 6.86–6.83 (m, 2H), 3.81 (s, 3H); ^{13}C {^{1}H} NMR (126 MHz, $CDCl_3$) δ = 160.5, 138.6, 133.5, 132.3, 130.9, 129.5, 129.0, 124.2, 118.0 (q, $J_{C\text{-}F}$ = 269.9 Hz), 114.2, 113.8, 94.7, 76.1, 55.5 ppm; ^{19}F NMR (376 MHz, $CDCl_3$) δ = –55.7 (s); HRMS (ESI) *m/z* calcd for $C_{18}H_{13}F_3N_3O$ $[M+H]^+$ 344.1005, found 344.1006.

X-ray crystallography. The single-crystal diffraction data of **1p** and **1q** were collected using Bruker D8 VENTURE system equipped with a Photon 100 CMOS detector, a multilayer monochromator, and a CuK*α* Incoatec microfocus sealed tube (λ = 1.54178 Å) at 180 K. The frames were integrated with the with Bruker SAINT[4] software package. The structures were solved by charge-flipping methods using Superflip[5] (**1p**) or by direct methods with SIR92[6] (**1q**) or and refined by full-matrix least-squares on F^2 with CRYSTALS.[7] The positional and anisotropic thermal parameters of all non-hydrogen atoms were refined. All hydrogen atoms were located in a difference Fourier map and then they were repositioned geometrically. They were initially refined with soft restraints on the bond lengths and angles to regularize their geometry, then their positions were refined with riding constraints.

Preparation of the crystal samples: Compound **1p** or **1q** (2 mg) were suspended in cyclohexane (1 ml) in a glass vial. Diethyl ether was then slowly added dropwise until complete dissolution of the solid was achieved. The vial was partially sealed with a plastic cap, and the resulting saturated solution was left to evaporate slowly at room temperature over several days. The vial was inspected daily until crystals of suitable size formed.

Crystal data for ***1p*** *(colorless, 0.063 x 0.085 x 0.121 mm):* $C_{10}H_5F_5IN_3$, monoclinic, space group $P2_1/c$, a = 6.2041(6) Å, b = 23.670(2) Å, c = 8.5735(8) Å, β = 104.009(4)°, V = 1221.6(2) Å^3, Z = 4, M = 389.06, 26784 reflections measured, 2326 independent reflections. Final R = 0.1137, wR = 0.3084, GoF = 0.9846 for 1643 reflections with I > 2σ(I) and 172 parameters. CCDC 2447758.

Crystal data for ***1q*** *(colorless, 0.093 x 0.105 x 0.334 mm):* $C_{10}H_5F_5N_4O_2$, monoclinic, space group $P2_1/c$, a = 11.9441(7) Å, b = 7.9074(5) Å, c = 12.8559(8) Å, β = 94.852(3)°, V = 1209.85(13) Å^3, Z = 4, M = 308.17, 19383 reflections measured, 2300 independent reflections. Final R = 0.0366, wR = 0.1031, GoF = 0.9735 for 2036 reflections with I > 2σ(I) and 191 parameters. CCDC 2447759.

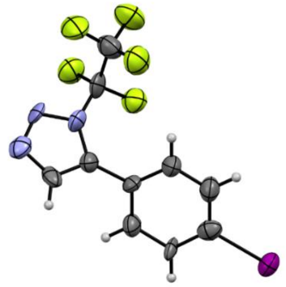

Figure SI1: Crystal structure of **1p**. Ellipsoids are at 50% probability level.

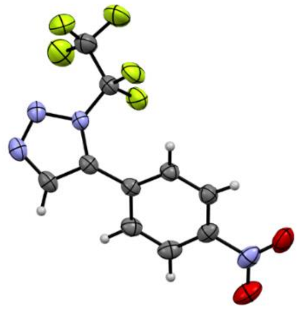

Figure SI2: Crystal structure of **1q**. Ellipsoids are at 50% probability level.

REFERENCES

(1) Blastik, Z. E.; Voltrová, S.; Matoušek, V.; Jurásek, B.; Manley, D. W.; Klepetářová, B.; Beier, P. Azidoperfluoroalkanes: Synthesis and Application in Copper(I)-Catalyzed Azide-Alkyne Cycloaddition. *Angew. Chem., Int. Ed.* **2017**, *56*, 346–349.

(2) Tichý, D.; Košťál, V.; Motornov, V.; Klimánková, I.; Beier, P. Preparation of 1-Azido-2-Bromo-1,1,2,2-Tetrafluoroethane and Its Use in the Synthesis of N-Fluoroalkylated Nitrogen Heterocycles. *J. Org. Chem.* **2020**, *85*, 11482–11489.

(3) Shaitanova, O.; Matoušek, V.; Herentin, T.; Adamec, M.; Matyáš, R.; Klepetářová, B.; Beier, P. Synthesis and Cycloaddition Reactions of 1-Azido-1,1,2,2-tetrafluoroethane. *J. Org. Chem.* **2023**, *88*, 14969–14977.

(4) SAINT. Bruker AXS Inc., Madison, Wisconsin, USA, 2015.

(5) Palatinus, L.; Chapuis, G. SUPERFLIP - a computer program for the solution of crystal structures by charge flipping in arbitrary dimensions. *J. Appl. Cryst.* **2007**, *40*, 786–790.

(6) Altomare, A.; Cascarano, G.; Giacovazzo G.; Guagliardi A.; Burla M. C.; Polidori, G.; Camalli, M. SIR92 – a program for automatic solution of crystal structures by direct methods. *J. Appl. Cryst.* **1994**, *27*, 435.

(7) Betteridge, P. W.; Carruthers, J. R.; Cooper, R. I.; Prout, K., Watkin, D. J. CRYSTALS version 12: software for guided crystal structure analysis. *J. Appl. Cryst.* **2003**, *36*, 1487.

COPIES OF NMR SPECTRA

^{1}H NMR (400 MHz, $CDCl_3$) of 1a

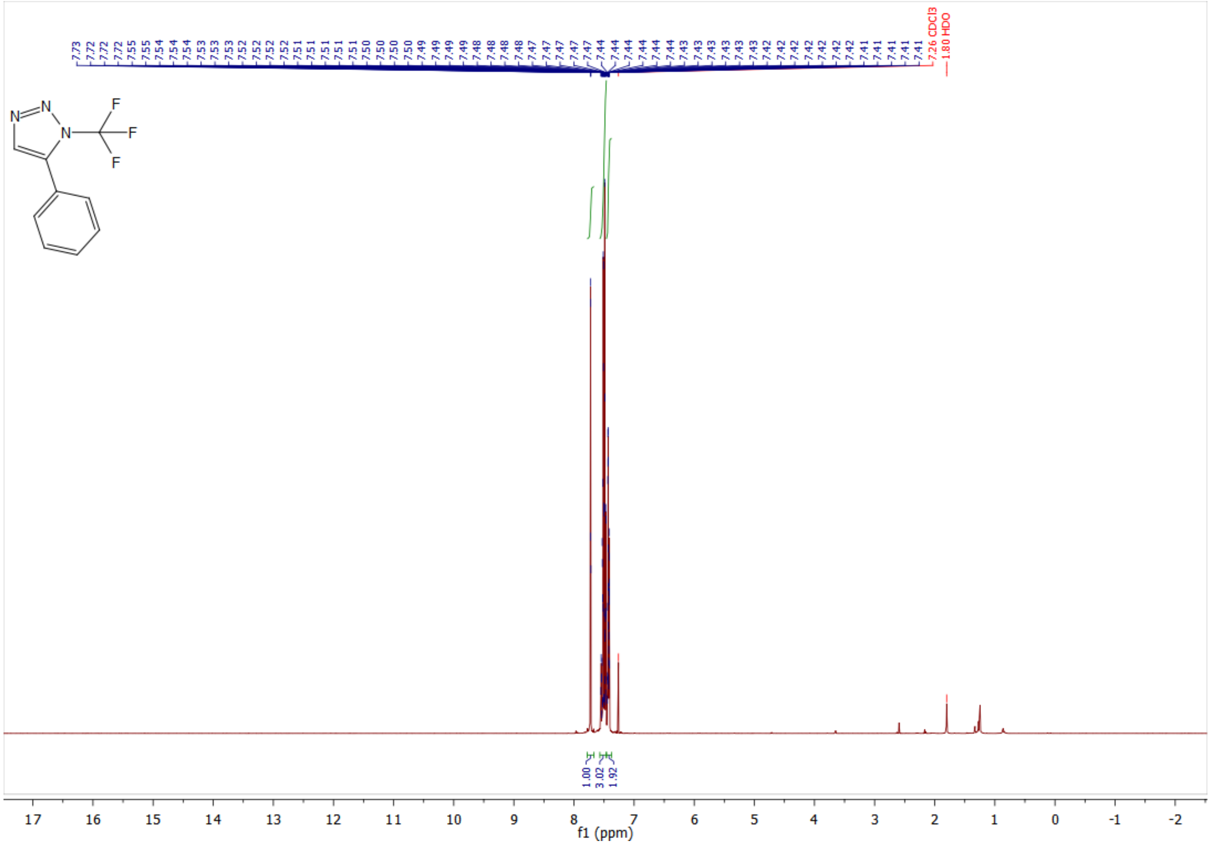

^{13}C {^{1}H} NMR (101 MHz, $CDCl_3$) of 1a

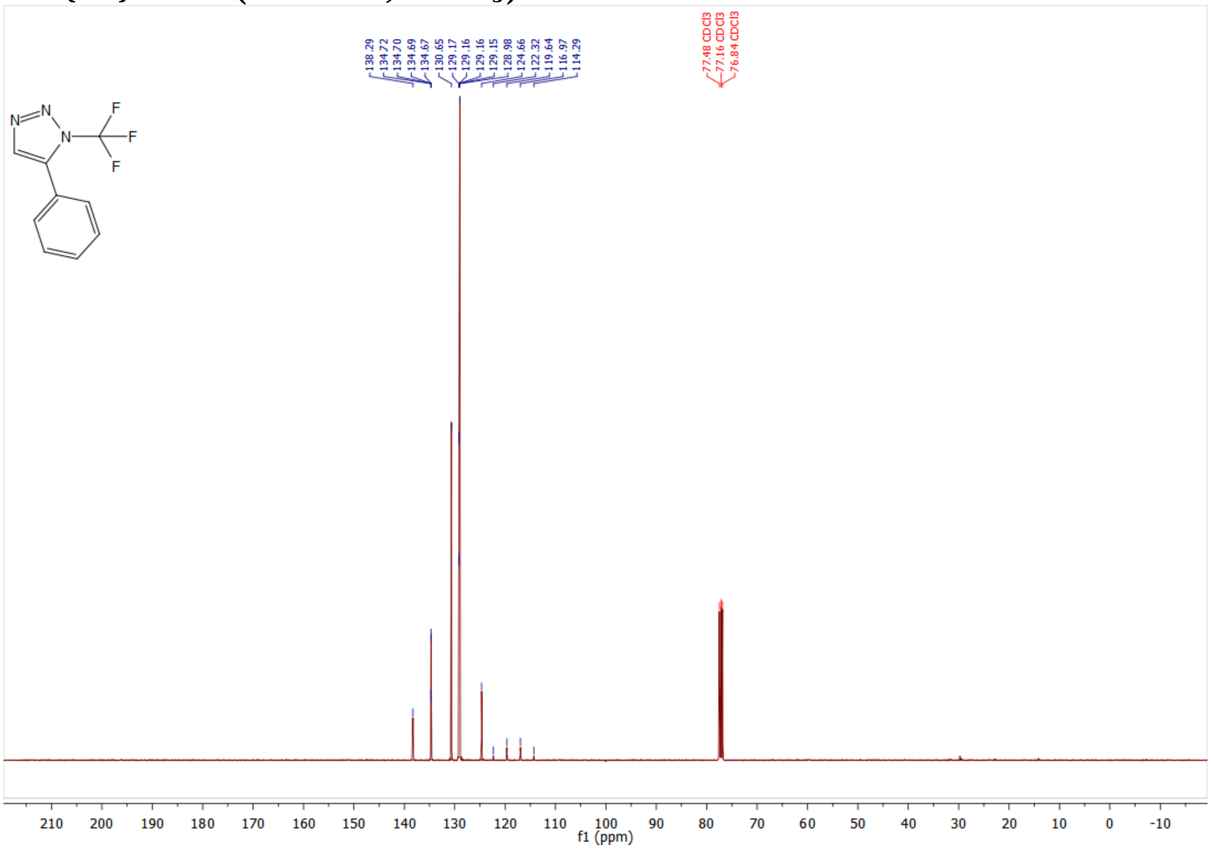

^{19}F NMR (377 MHz, $CDCl_3$) of 1a

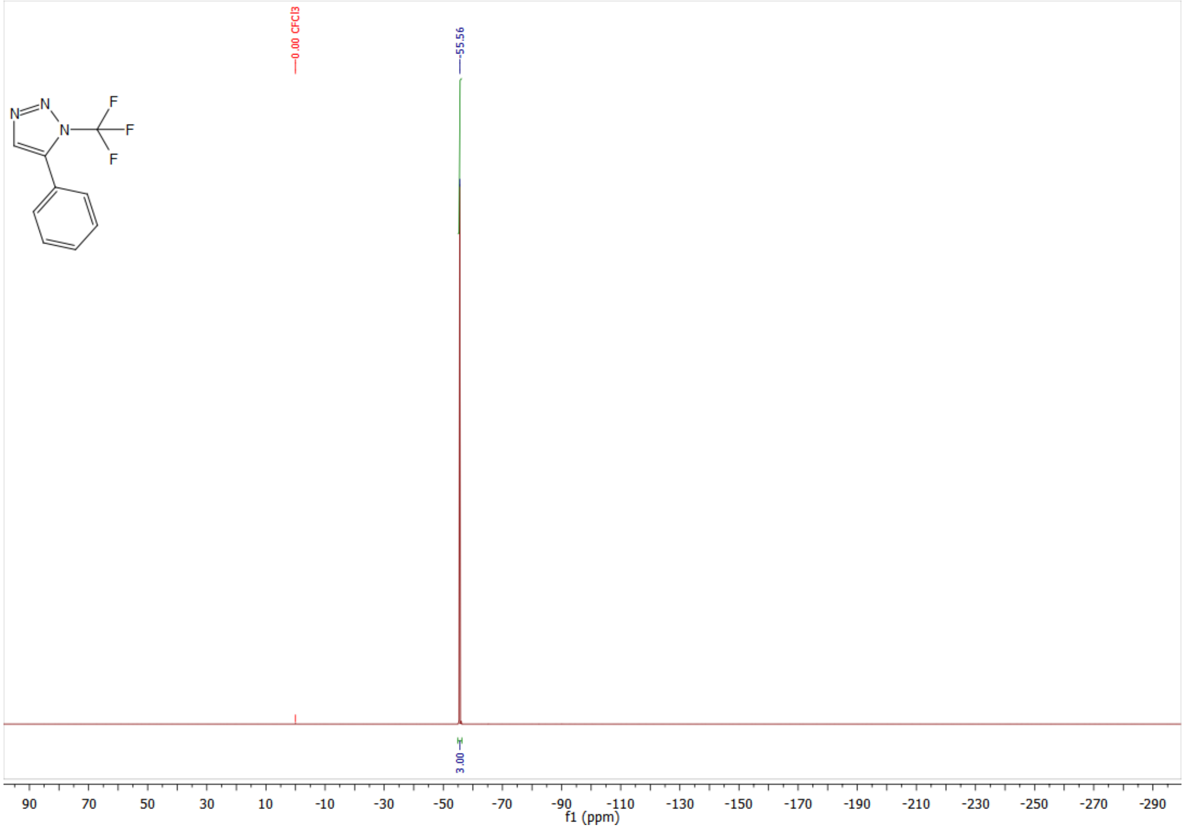

^{1}H NMR (400 MHz, $CDCl_3$) of 1b

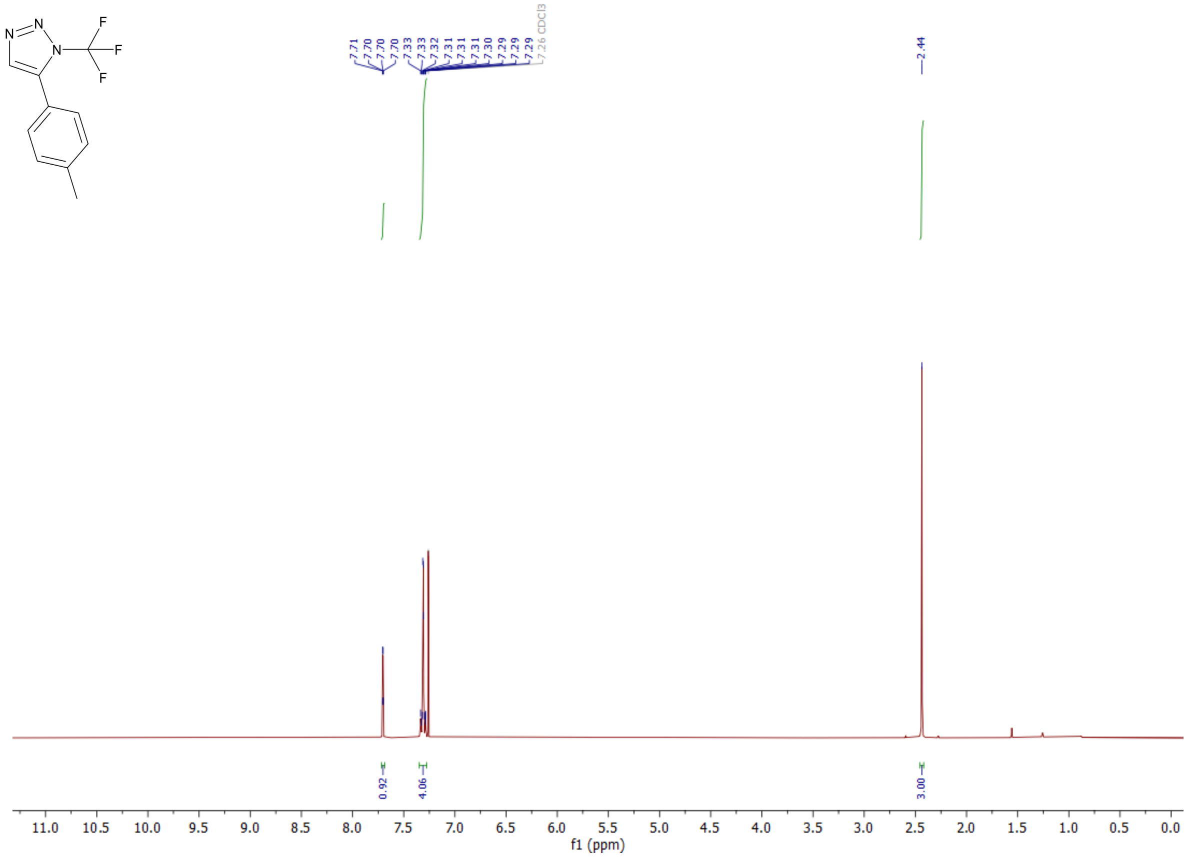

$^{13}C\{^1H\}$ NMR (101 MHz, $CDCl_3$) of 1b

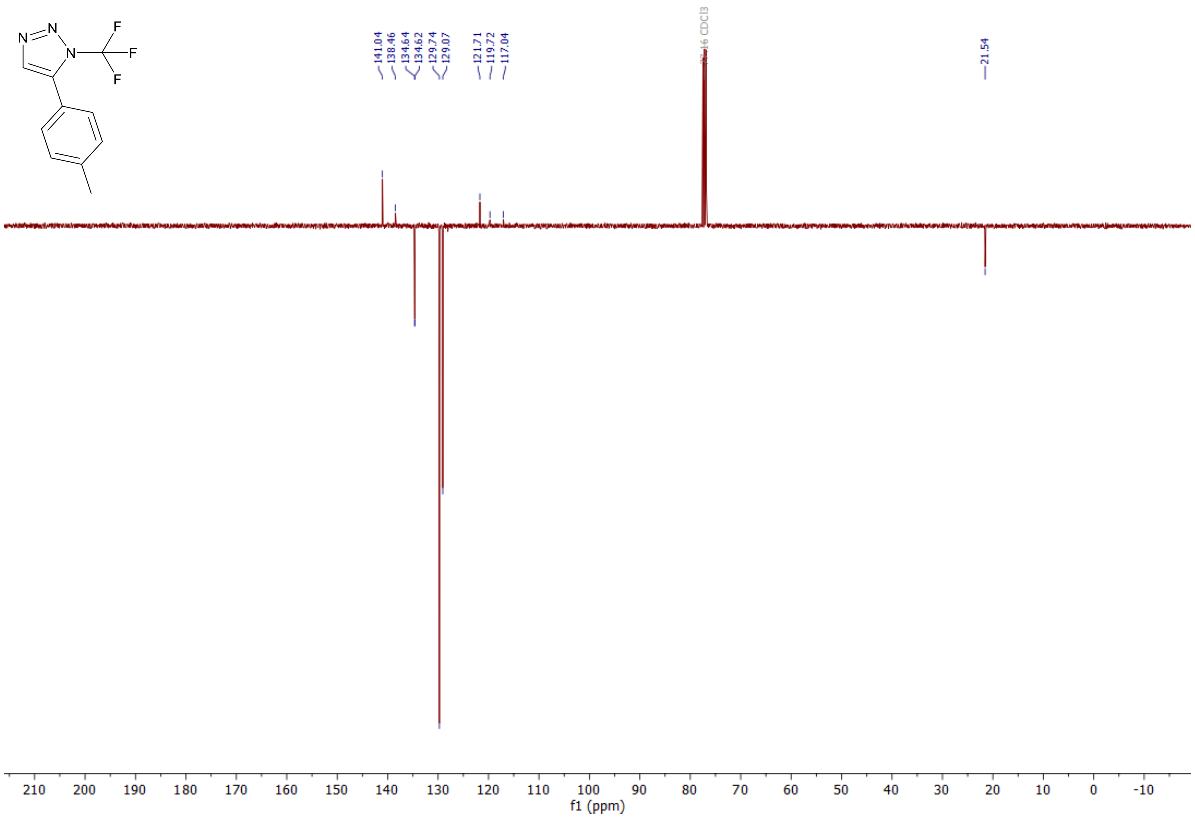

^{19}F NMR (377 MHz, $CDCl_3$) of 1b

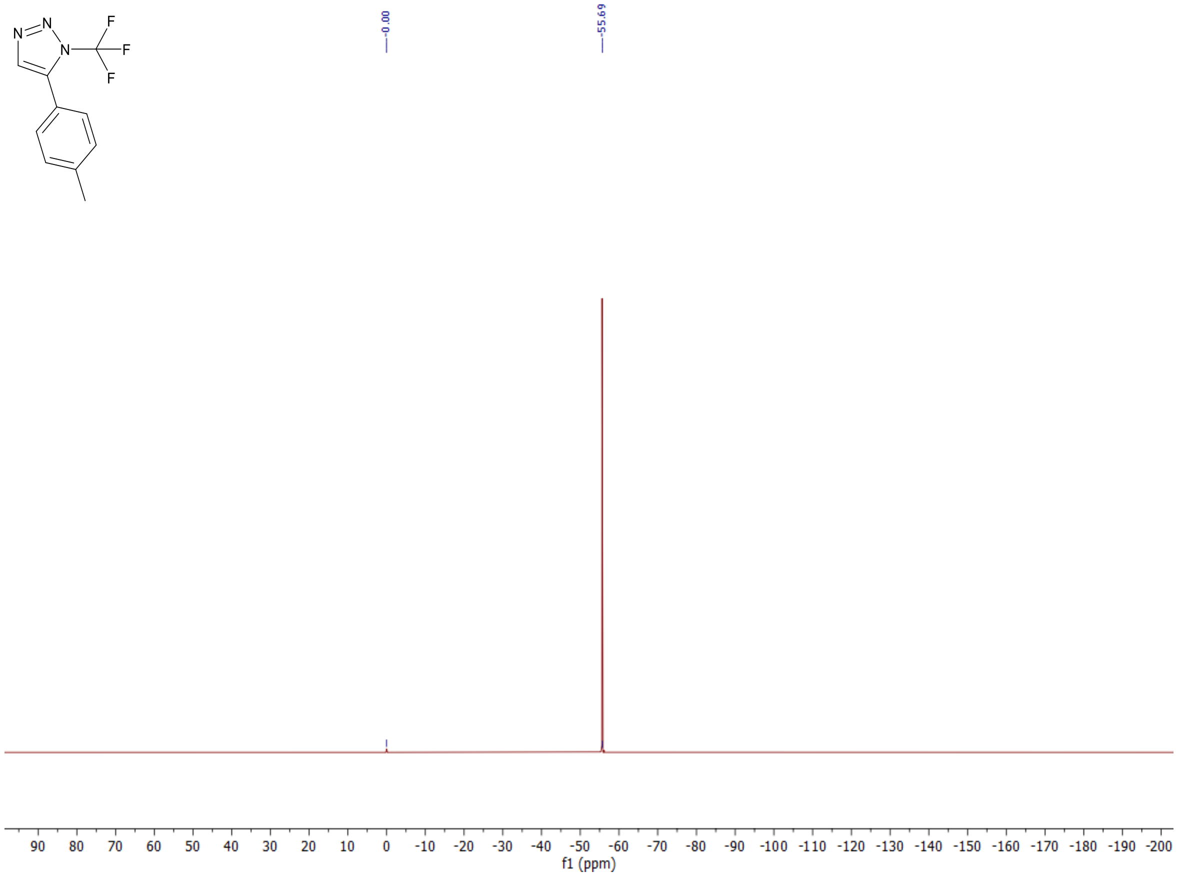

^{1}H NMR (400 MHz, d6-DMSO) of 1c

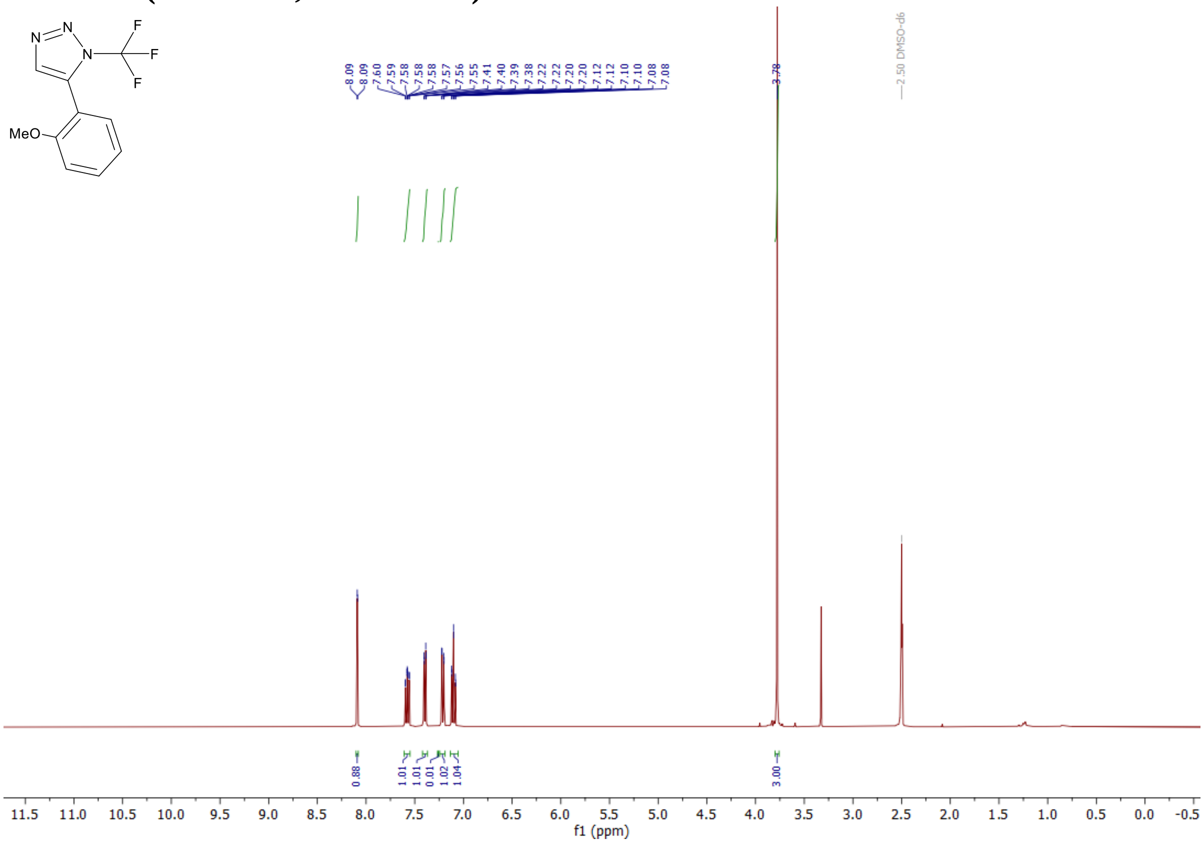

^{13}C {^{1}H} NMR (101 MHz, d6-DMSO) of 1c

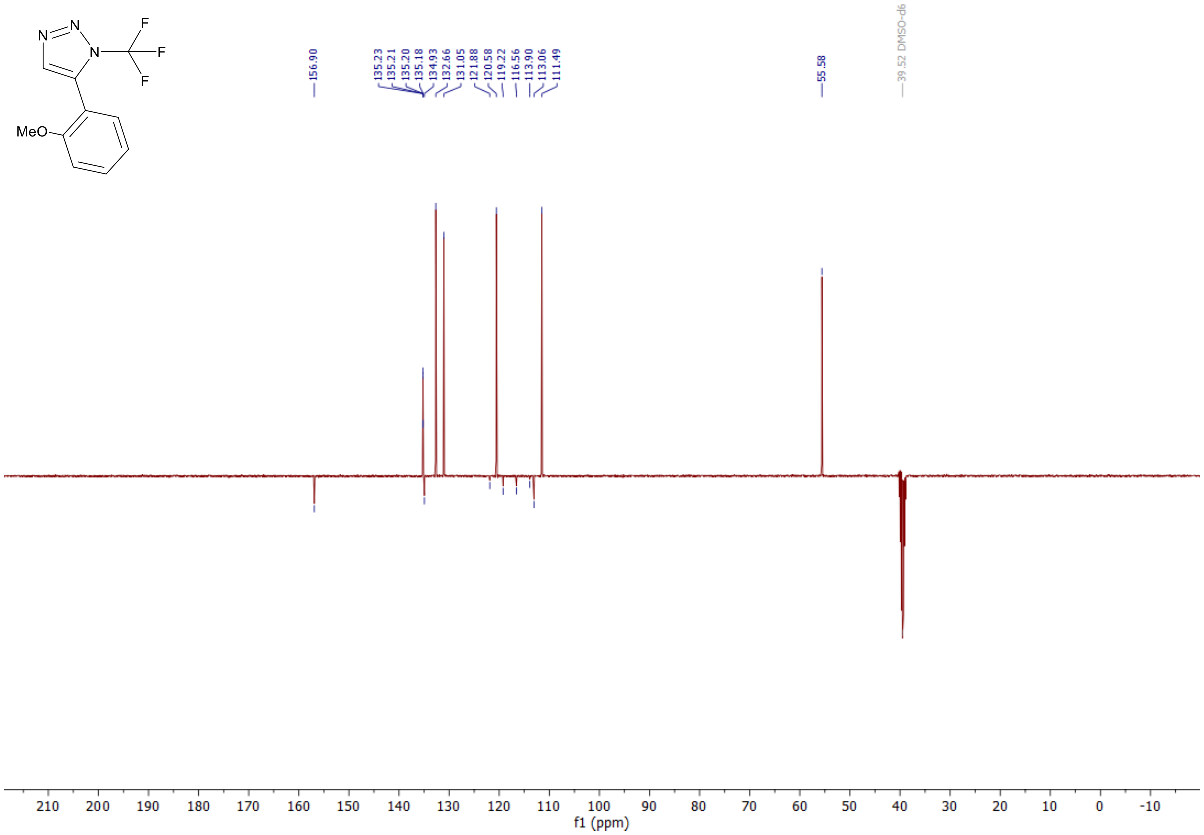

^{19}F NMR (377 MHz, d6-DMSO) of 1c

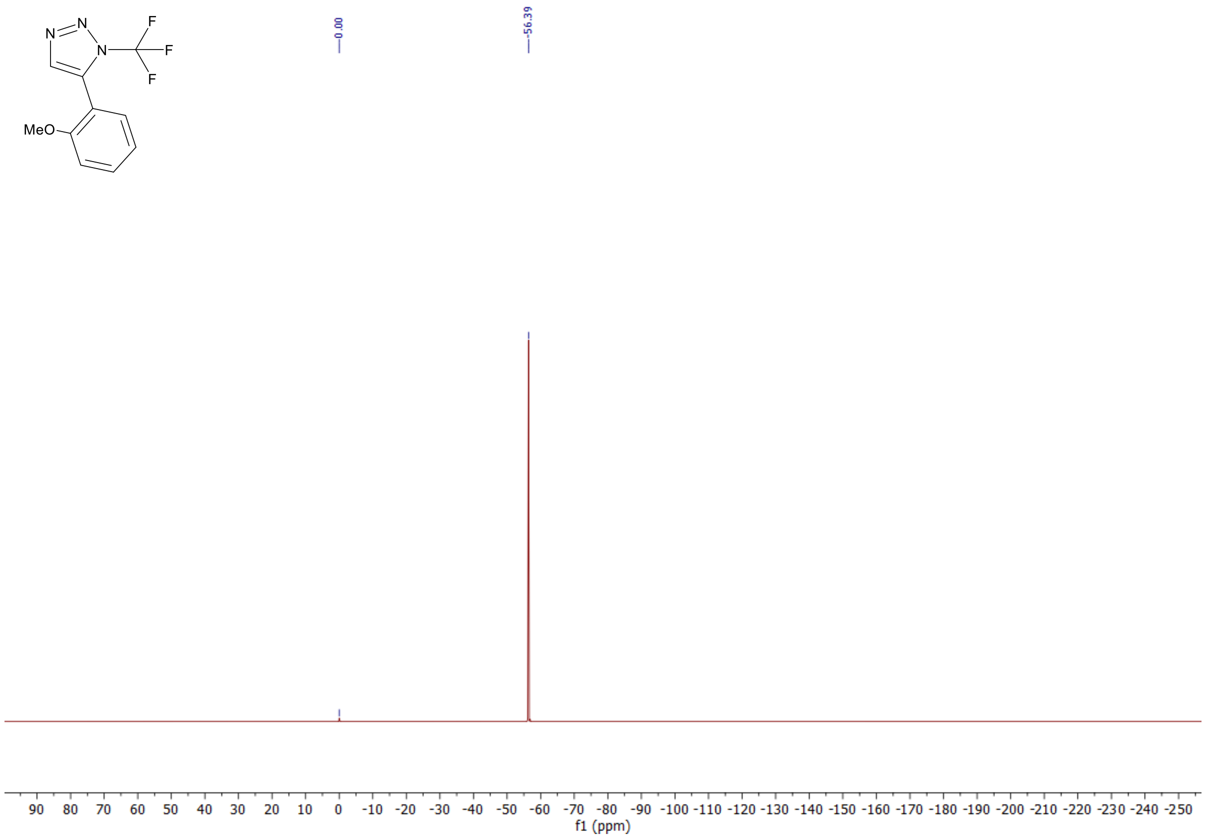

^{1}H NMR (400 MHz, $CDCl_3$) of 1d

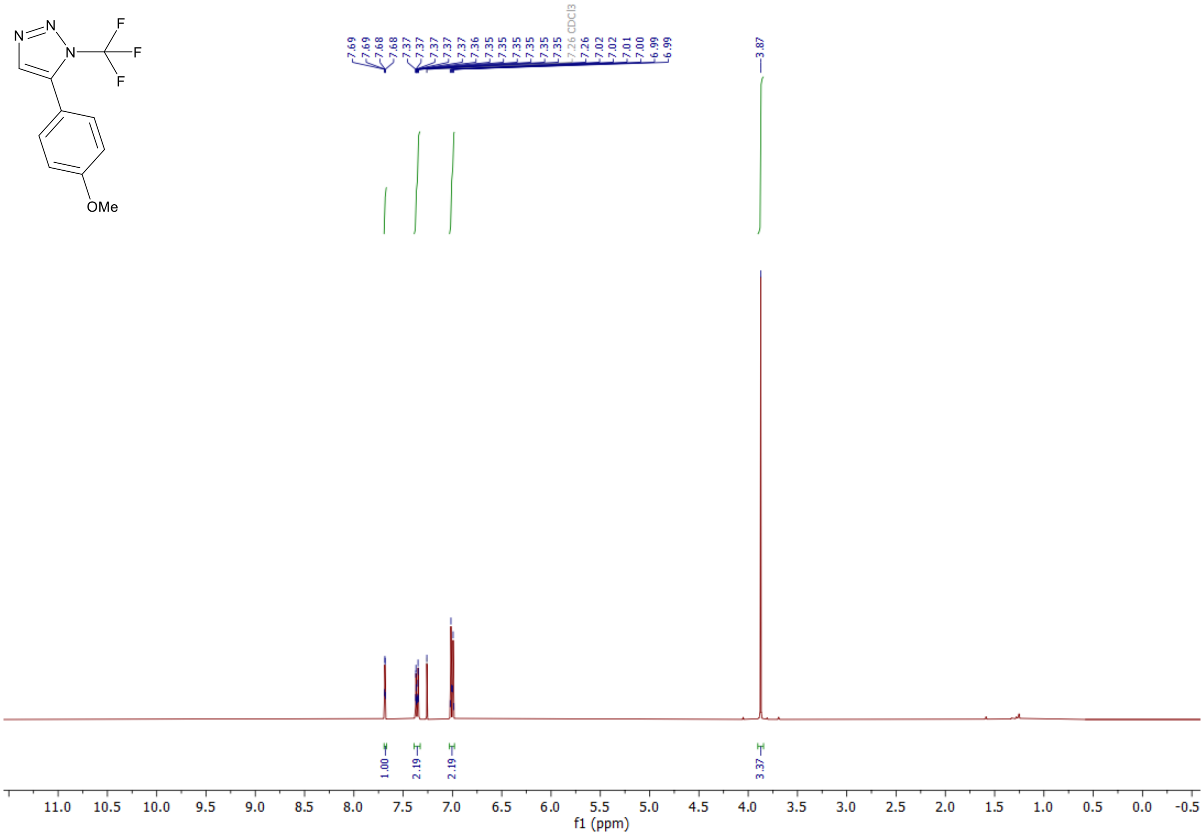

$^{13}C\ \{^{1}H\}$ NMR (101 MHz, $CDCl_3$) of 1d

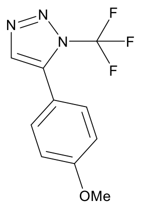

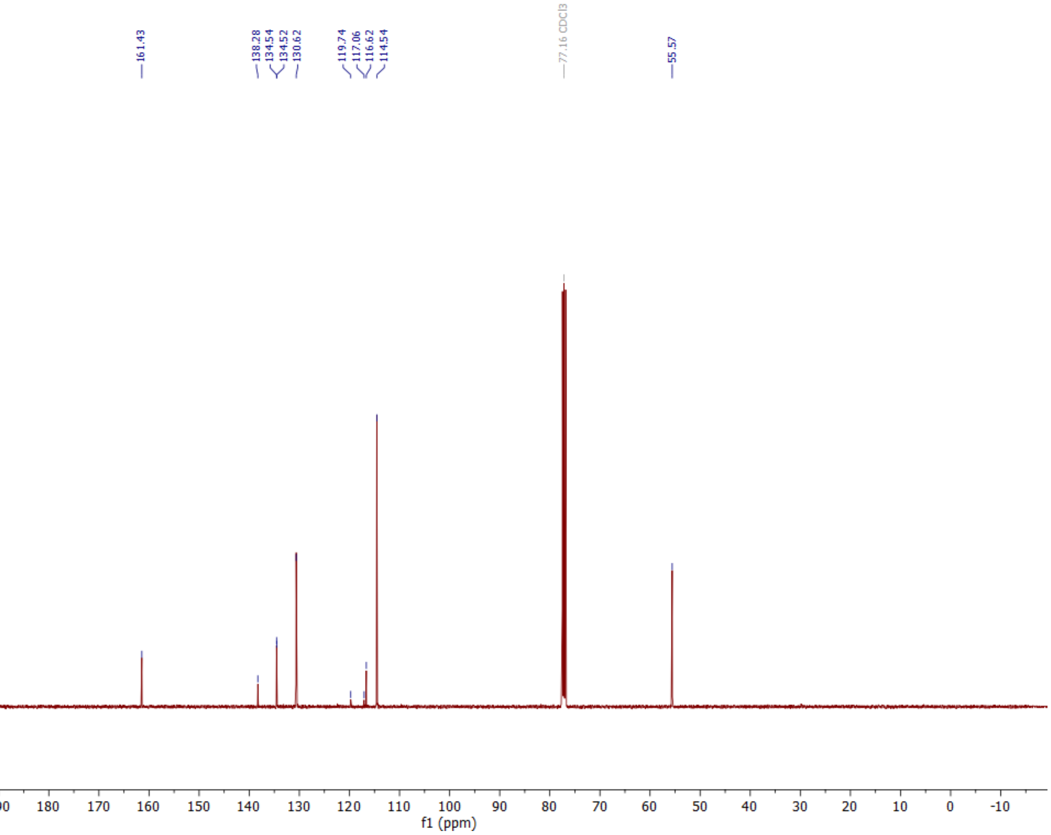

^{19}F NMR (377 MHz, $CDCl_3$) of 1d

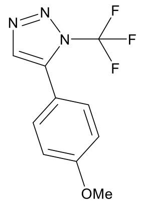

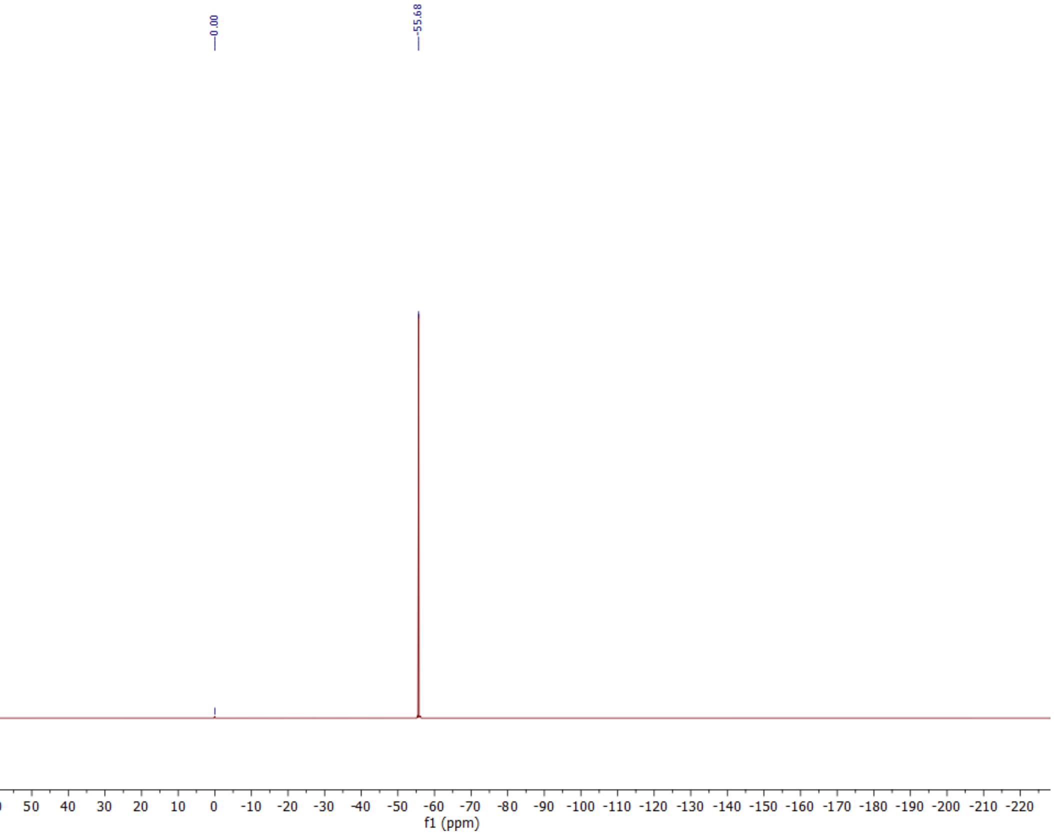

^{1}H NMR (400 MHz, $CDCl_3$) of 1e

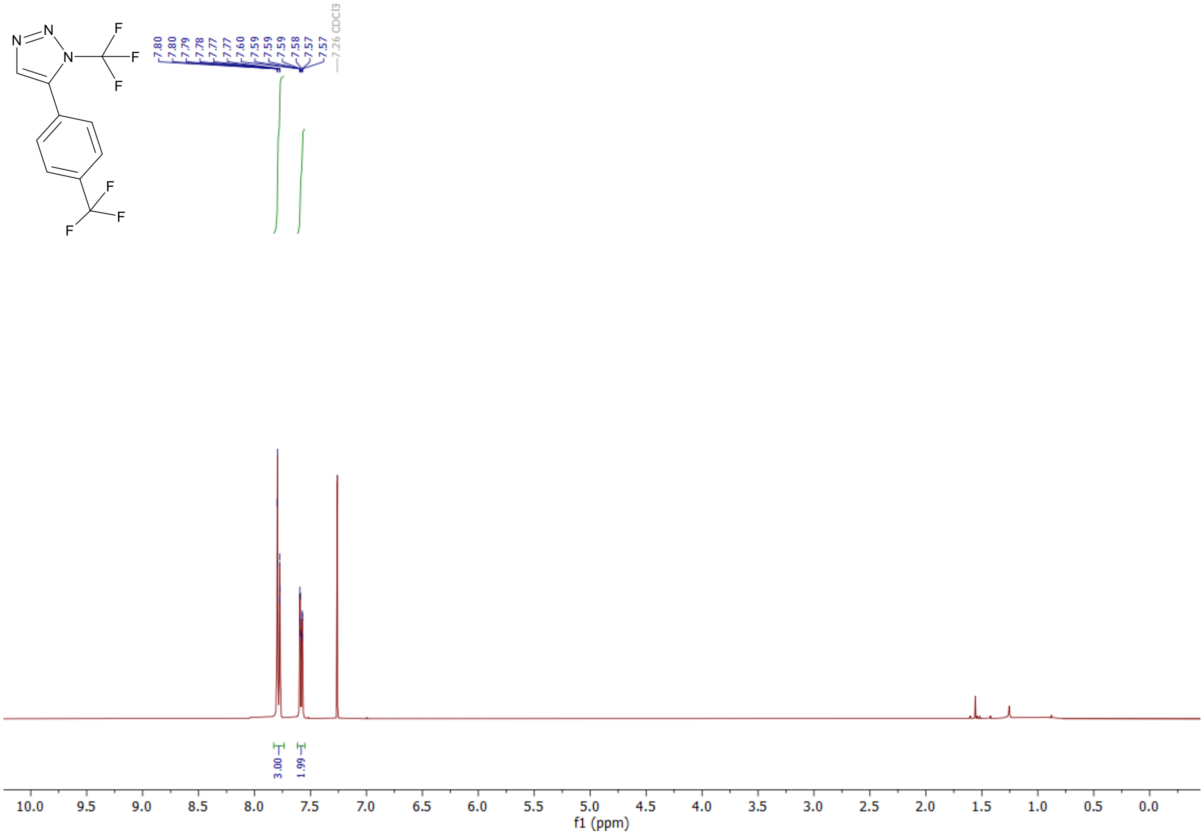

^{13}C {^{1}H} NMR (101 MHz, $CDCl_3$) of 1e

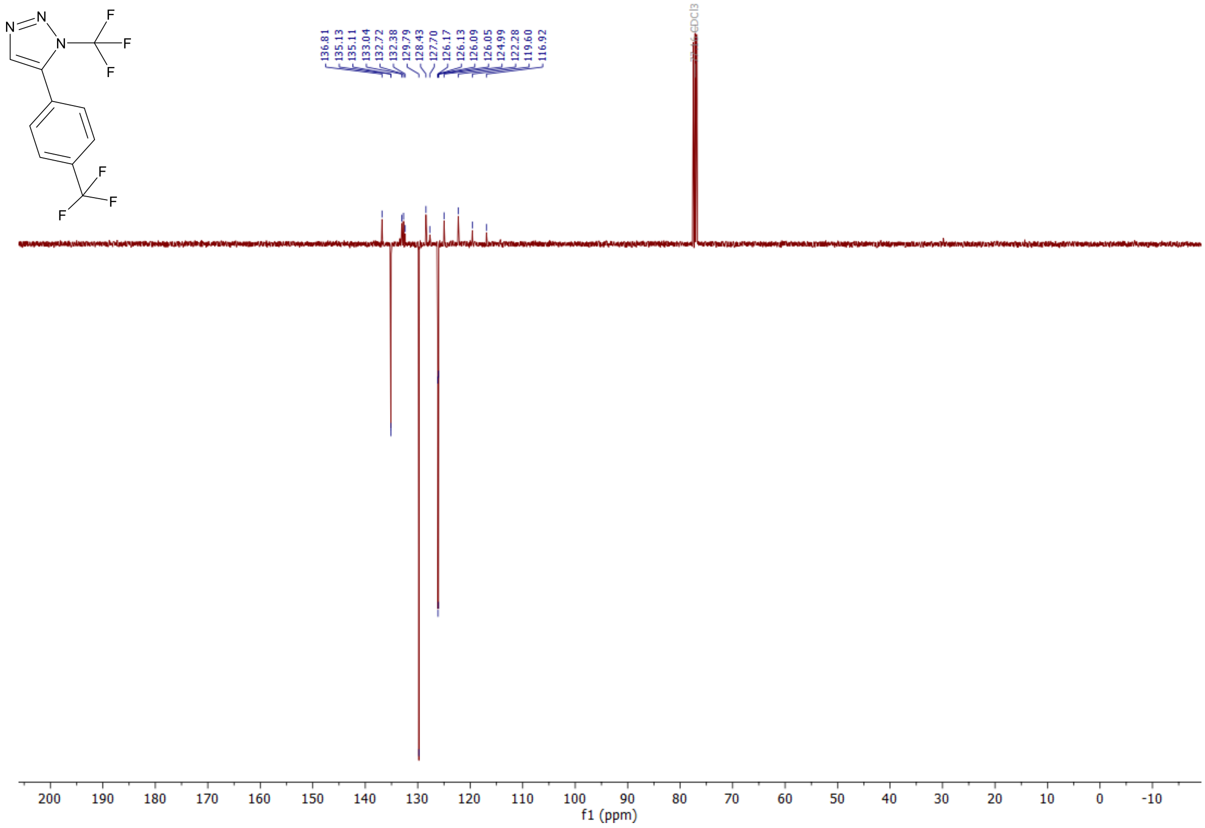

^{19}F NMR (377 MHz, $CDCl_3$) of 1e

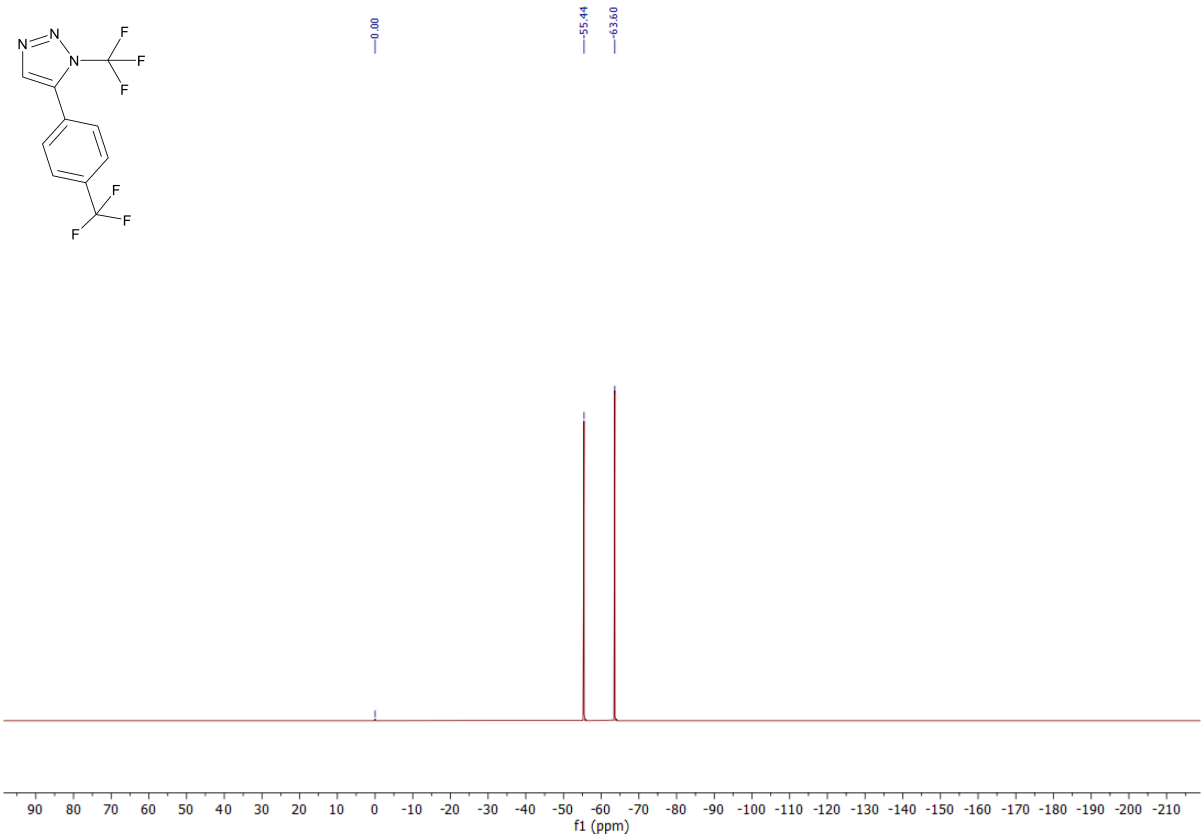

^{1}H NMR (400 MHz, $CDCl_3$) of 1f

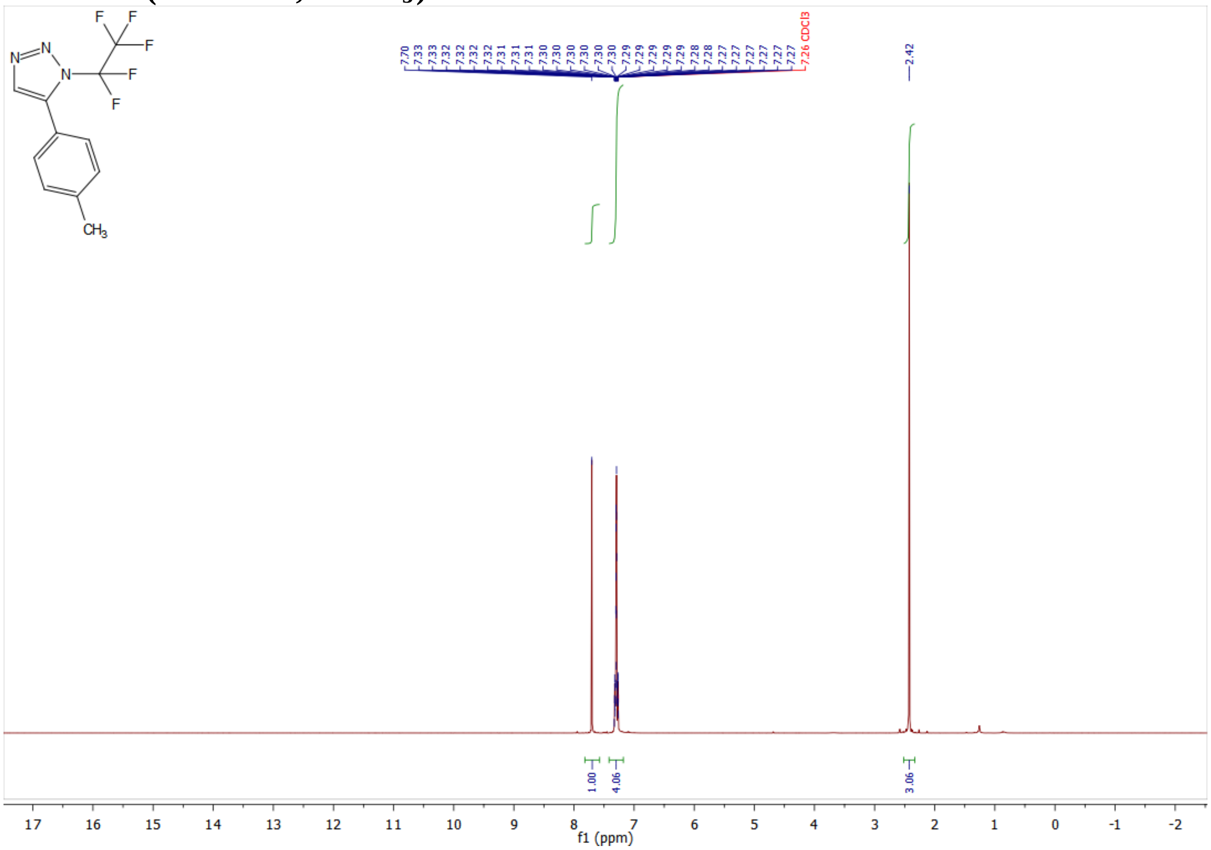

^{13}C {^{1}H} NMR (101 MHz, $CDCl_3$) of 1f

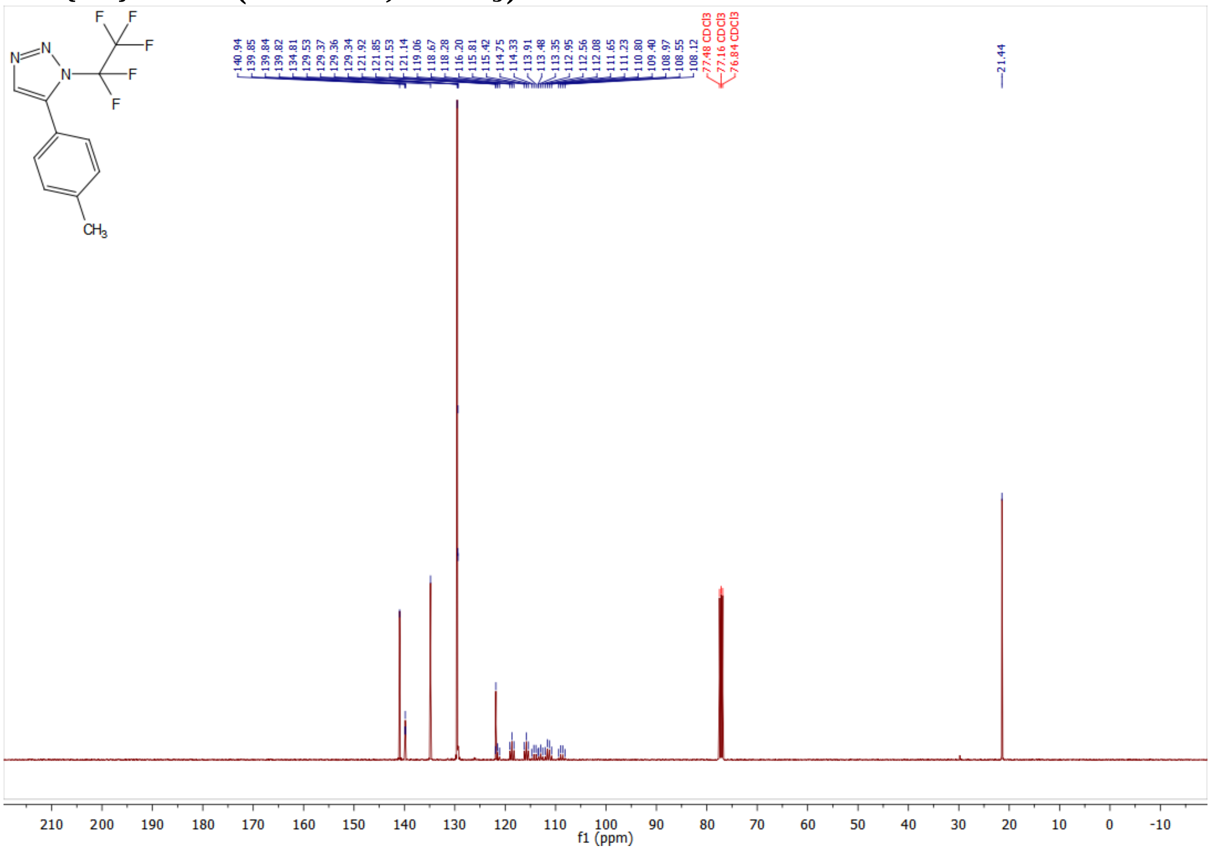

^{19}F NMR (377 MHz, $CDCl_3$) of 1f

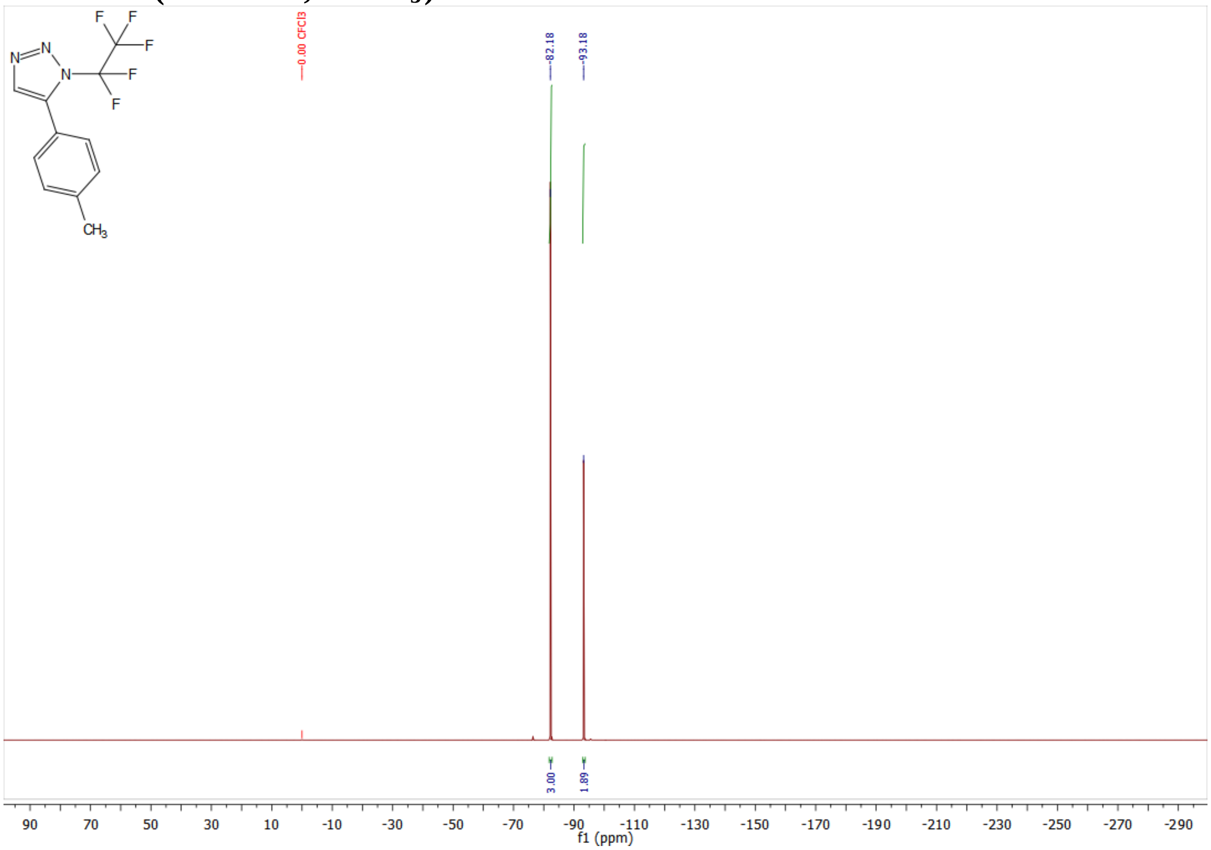

^{1}H NMR (400 MHz, $CDCl_3$) of 1g

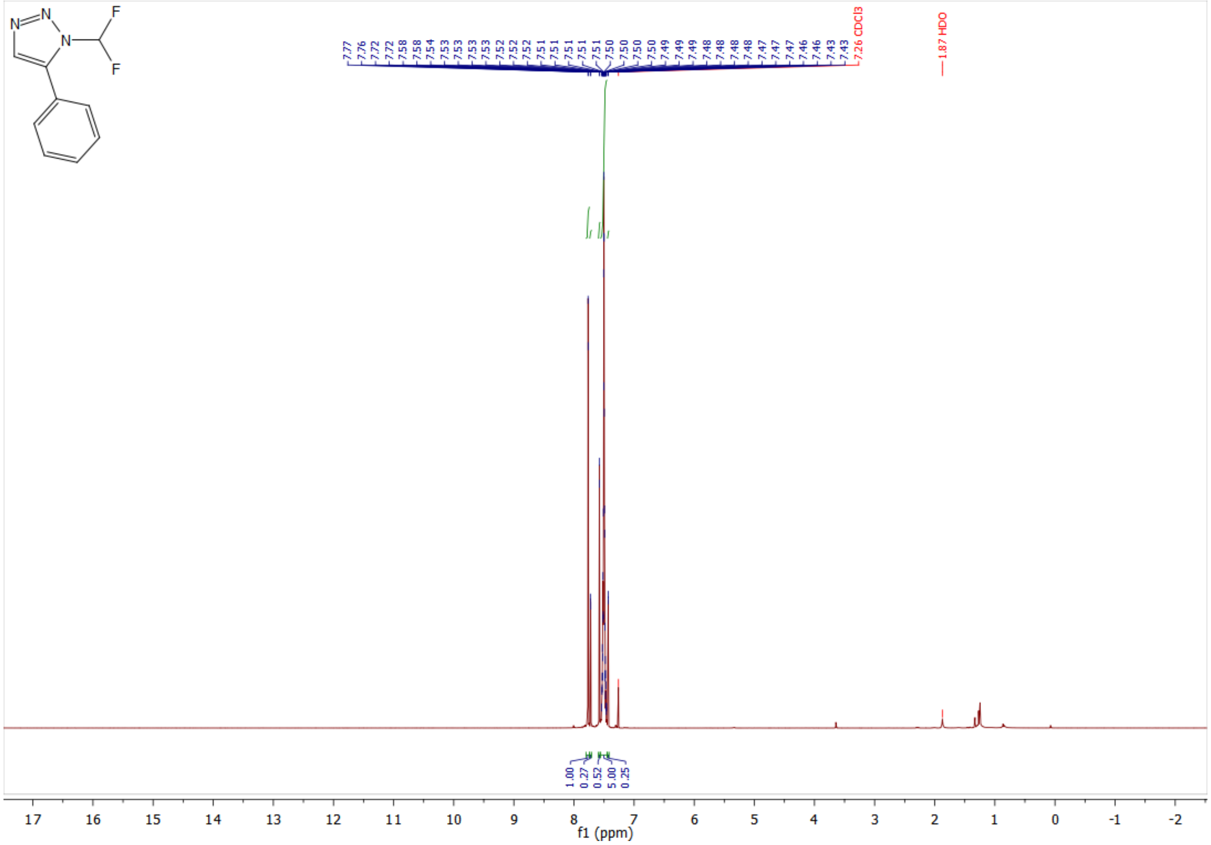

$^{13}C\{^{1}H\}$ NMR (101 MHz, $CDCl_3$) of 1g

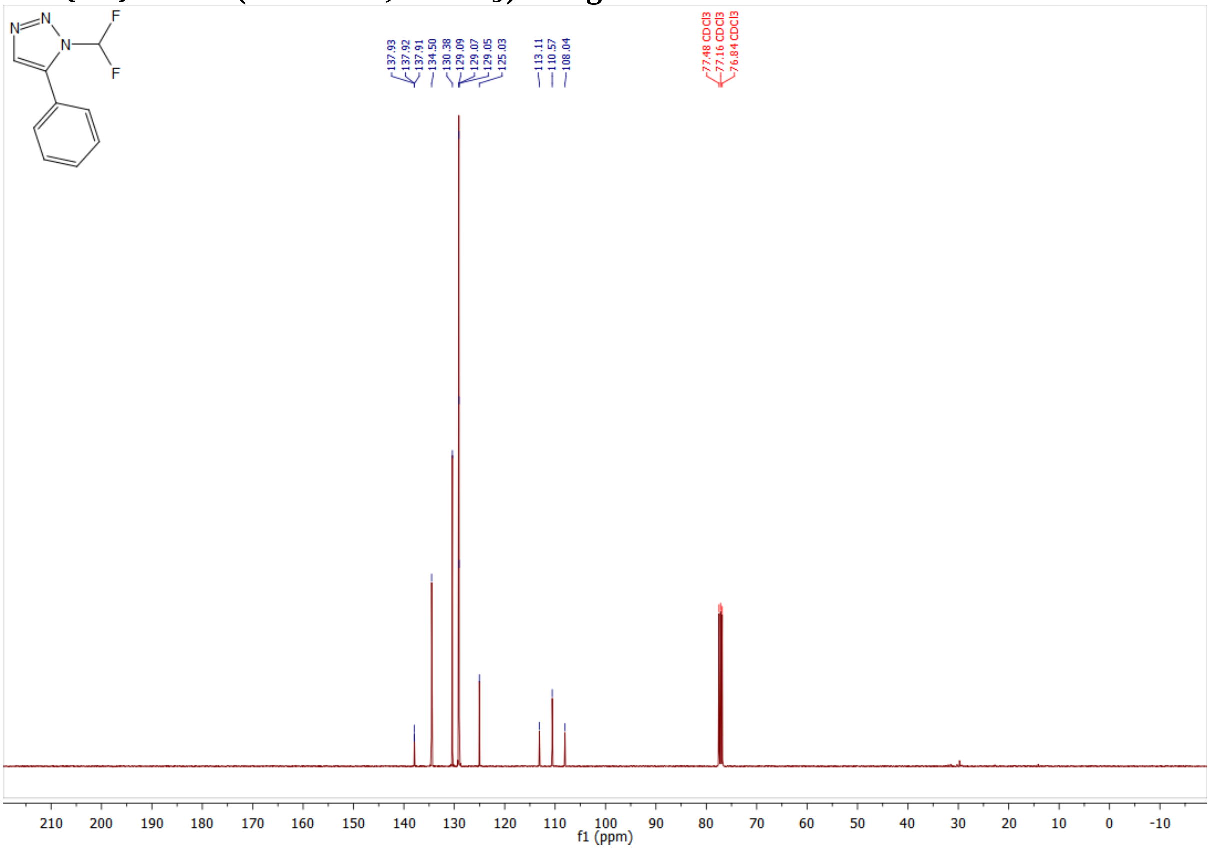

^{19}F NMR (377 MHz, $CDCl_3$) of 1g

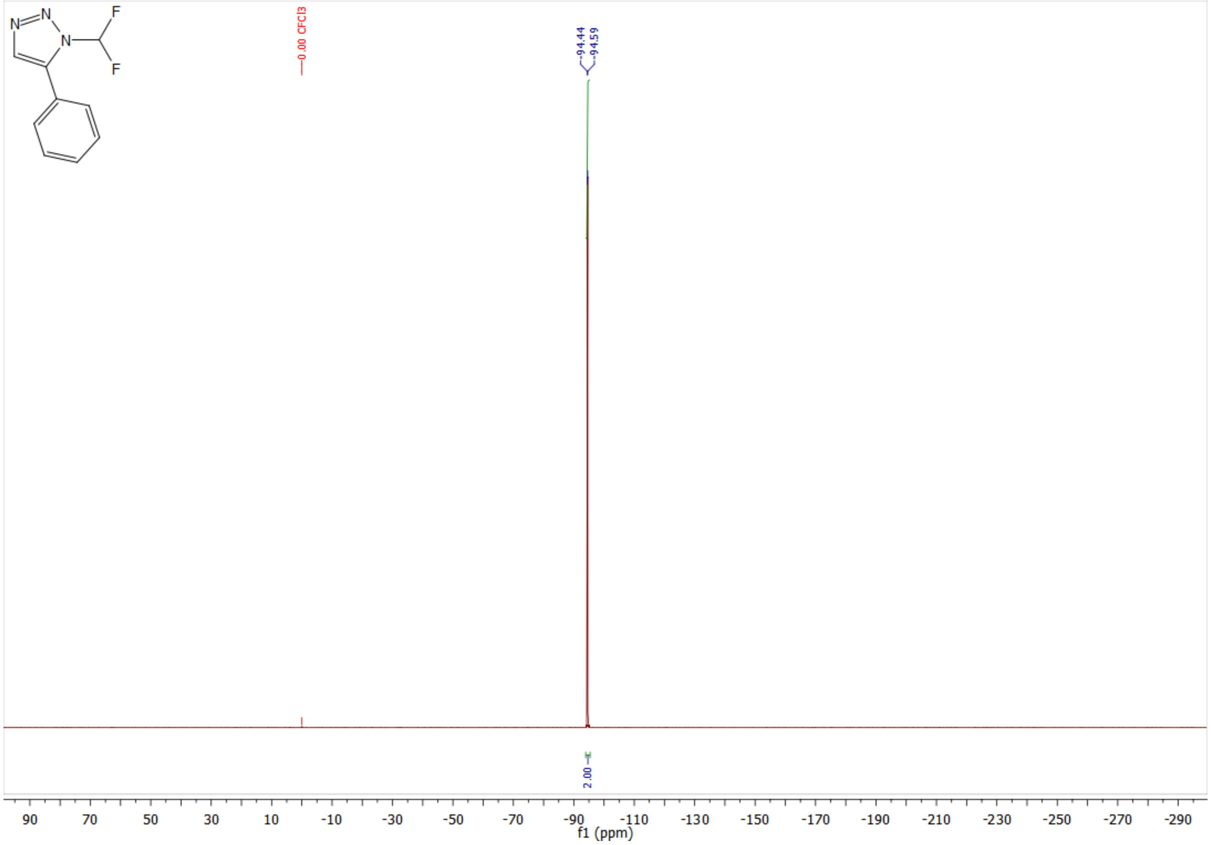

^{1}H NMR (400 MHz, $CDCl_3$) of 1h

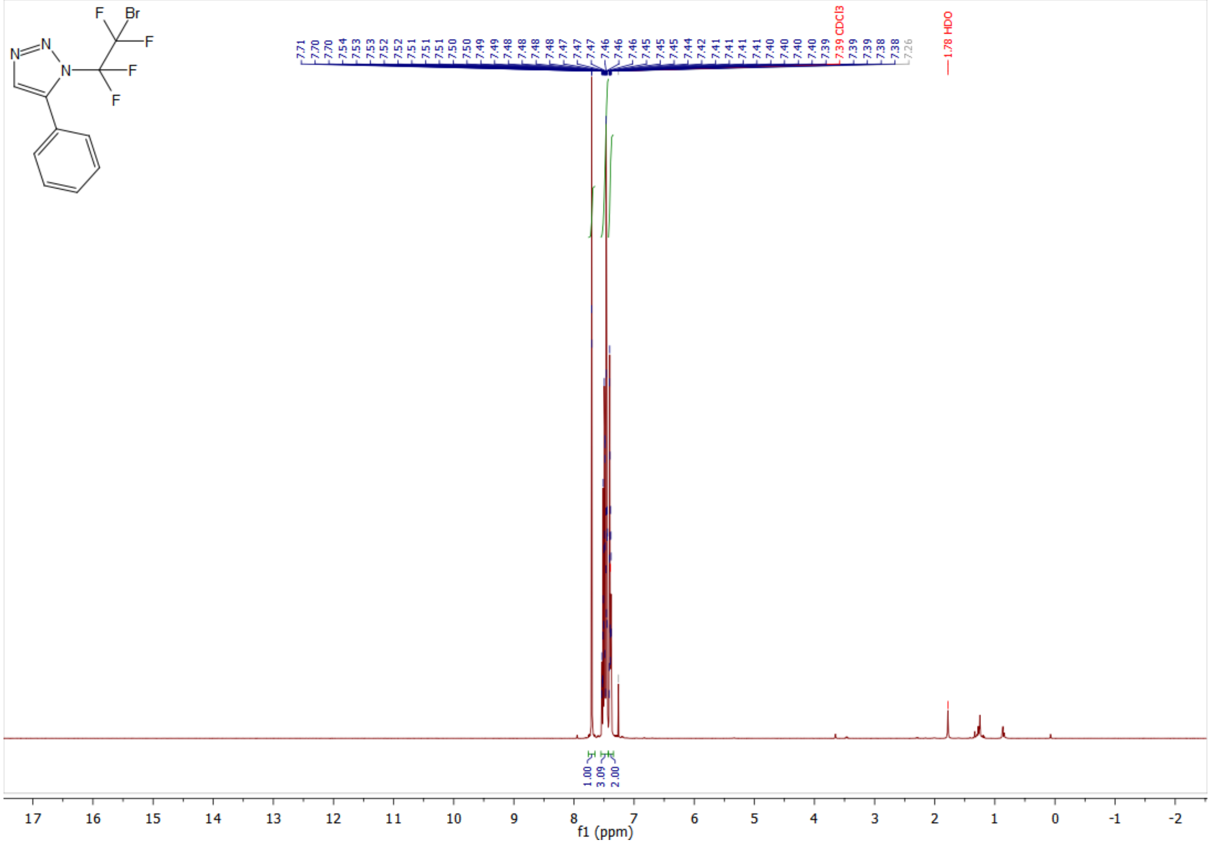

$^{13}C\ \{^{1}H\}$ NMR (101 MHz, $CDCl_3$) of 1h

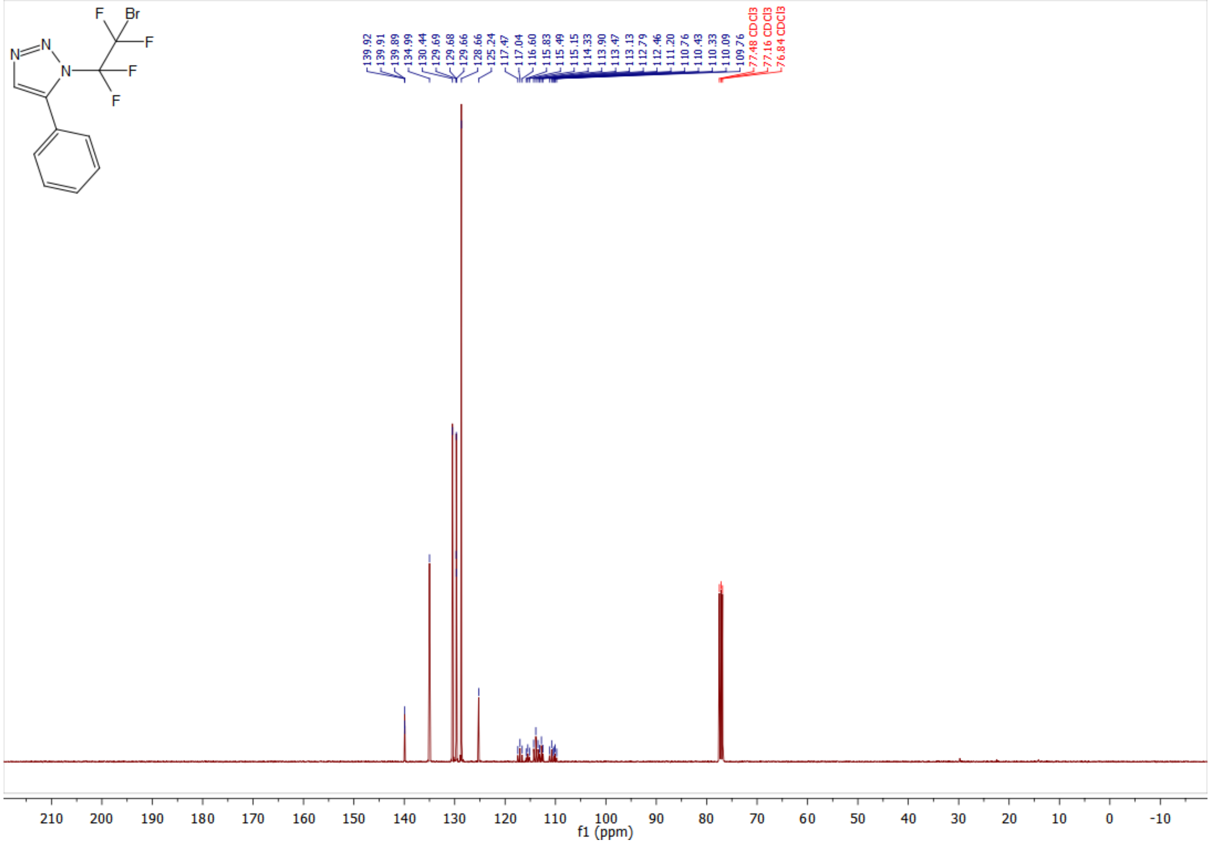

^{19}F NMR (377 MHz, $CDCl_3$) of 1h

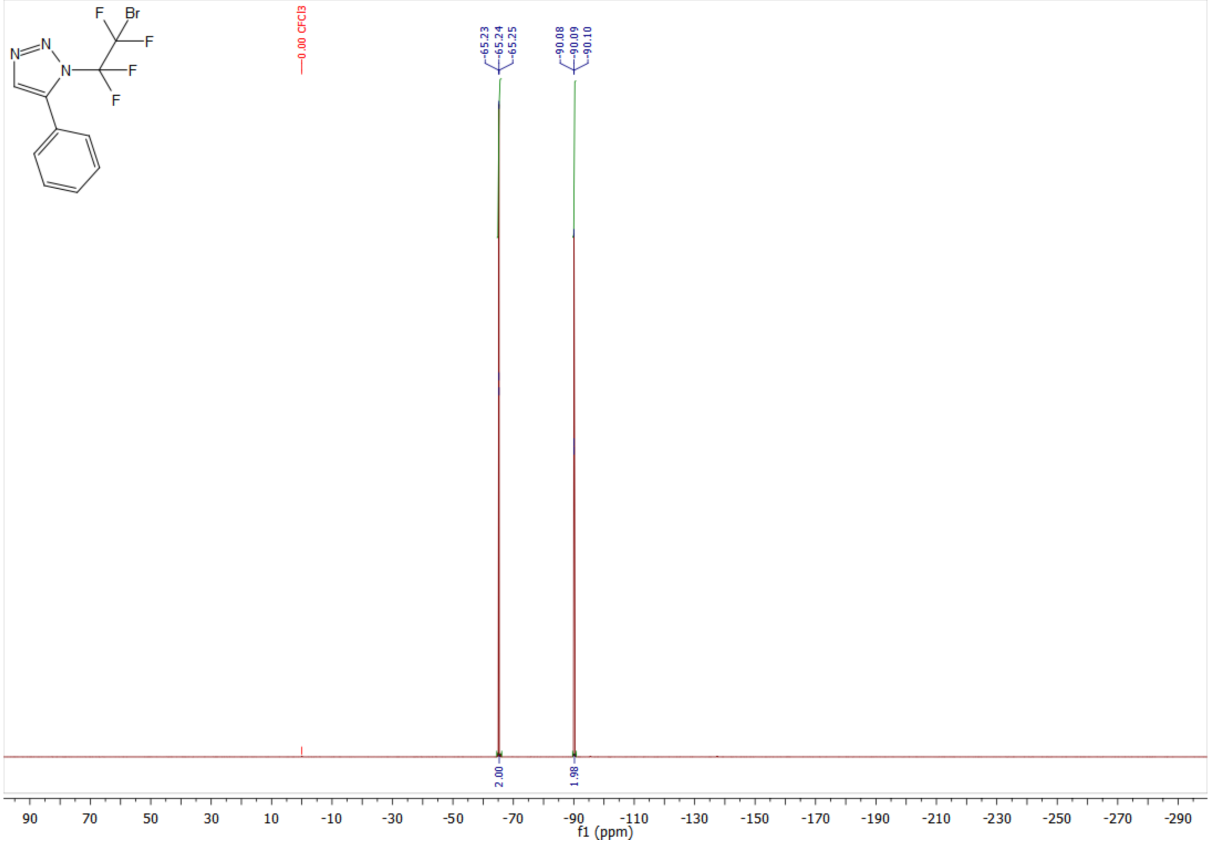

^{1}H NMR (400 MHz, $CDCl_3$) of 1i

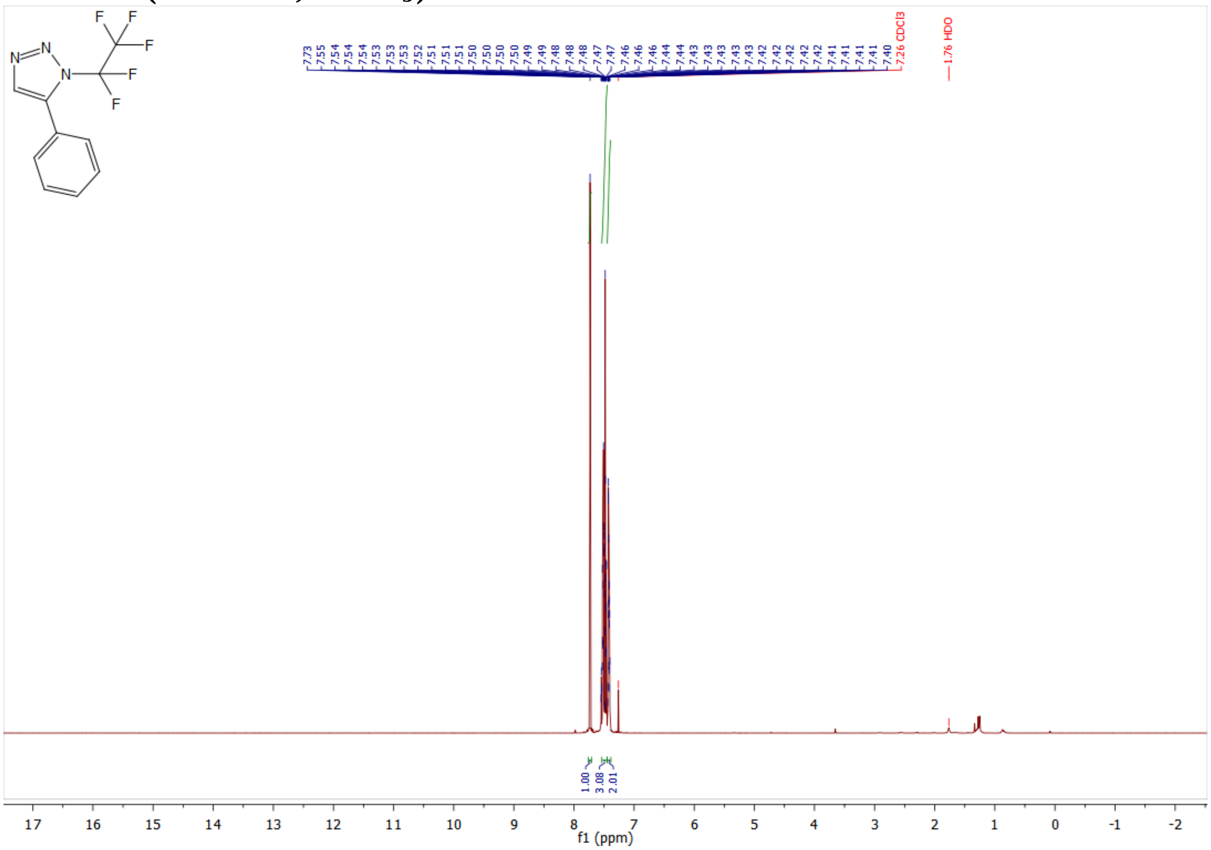

^{13}C {^{1}H} NMR (101 MHz, $CDCl_3$) of 1i

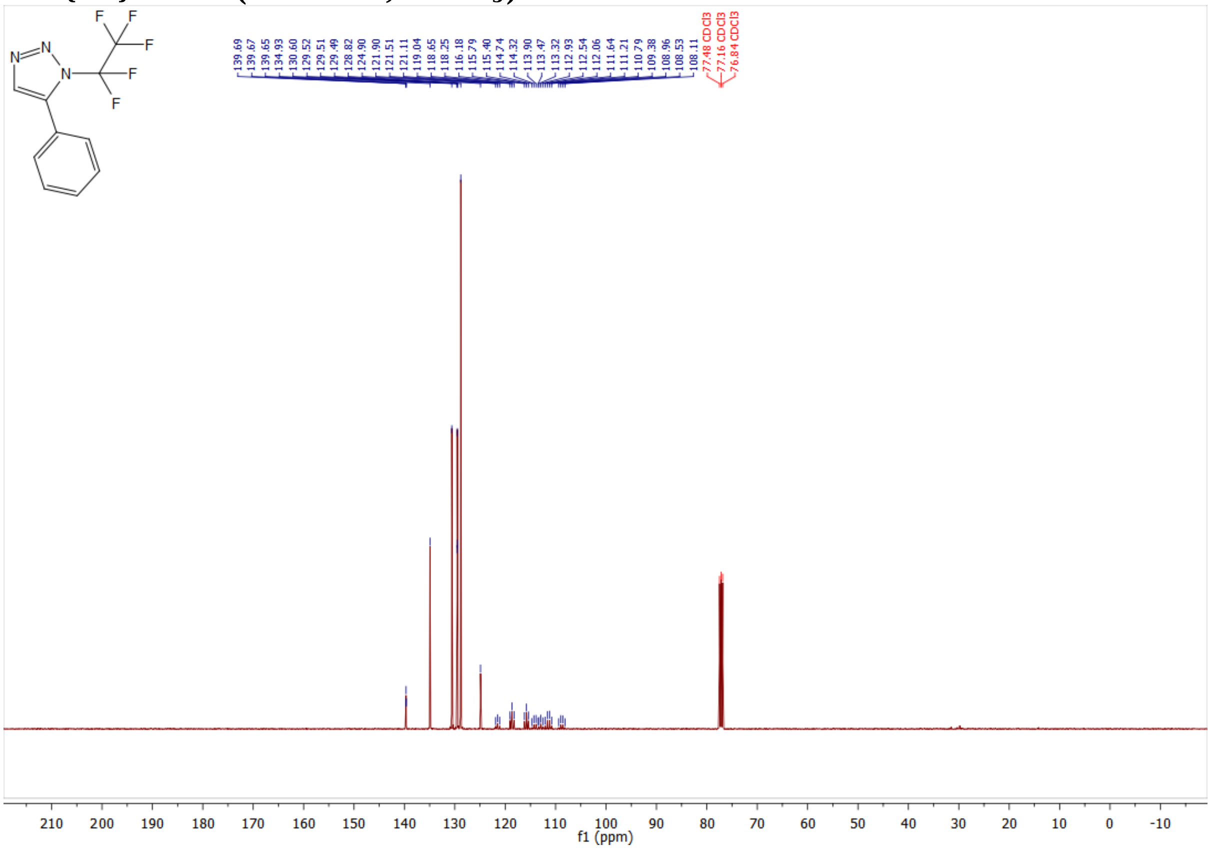

^{19}F NMR (377 MHz, $CDCl_3$) of 1i

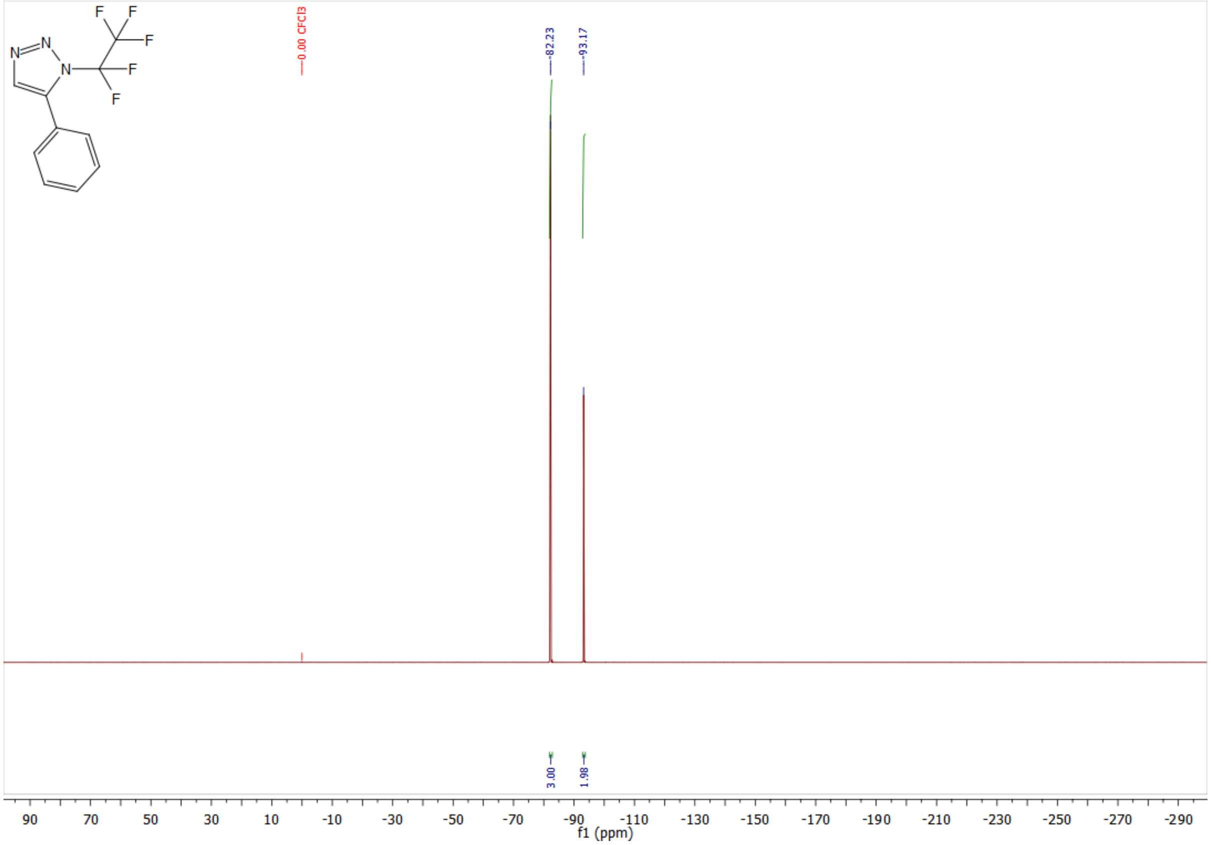

^{1}H NMR (400 MHz, $CDCl_3$) of 1j

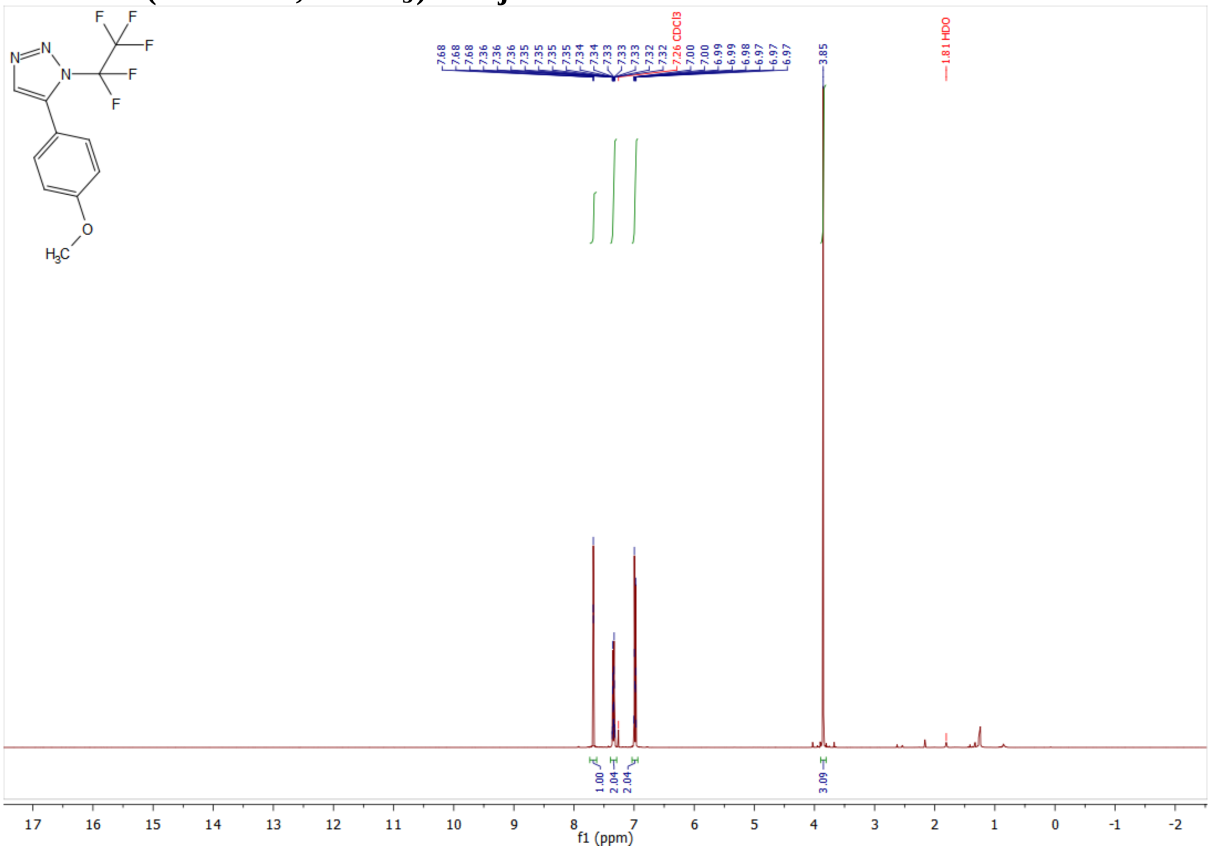

^{13}C {^{1}H} NMR (101 MHz, $CDCl_3$) of 1j

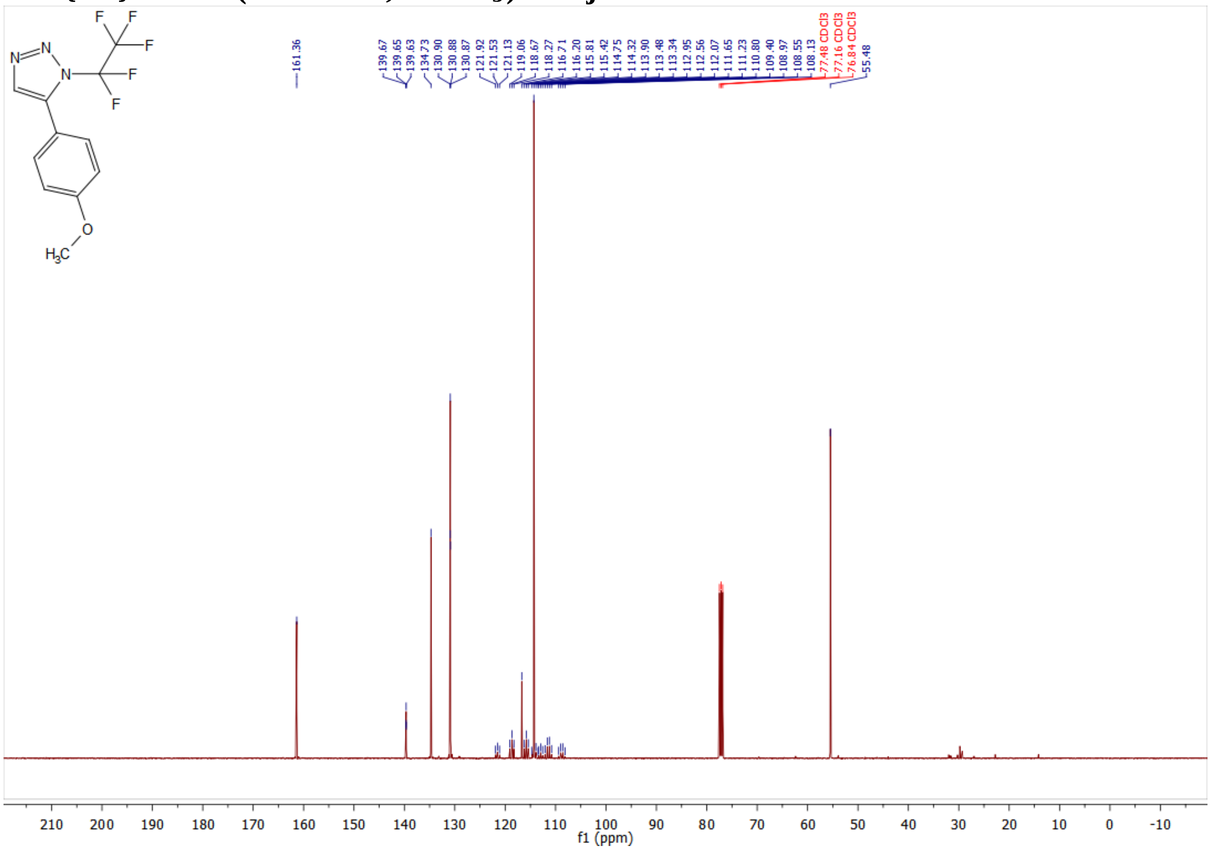

^{19}F NMR (377 MHz, $CDCl_3$) of 1j

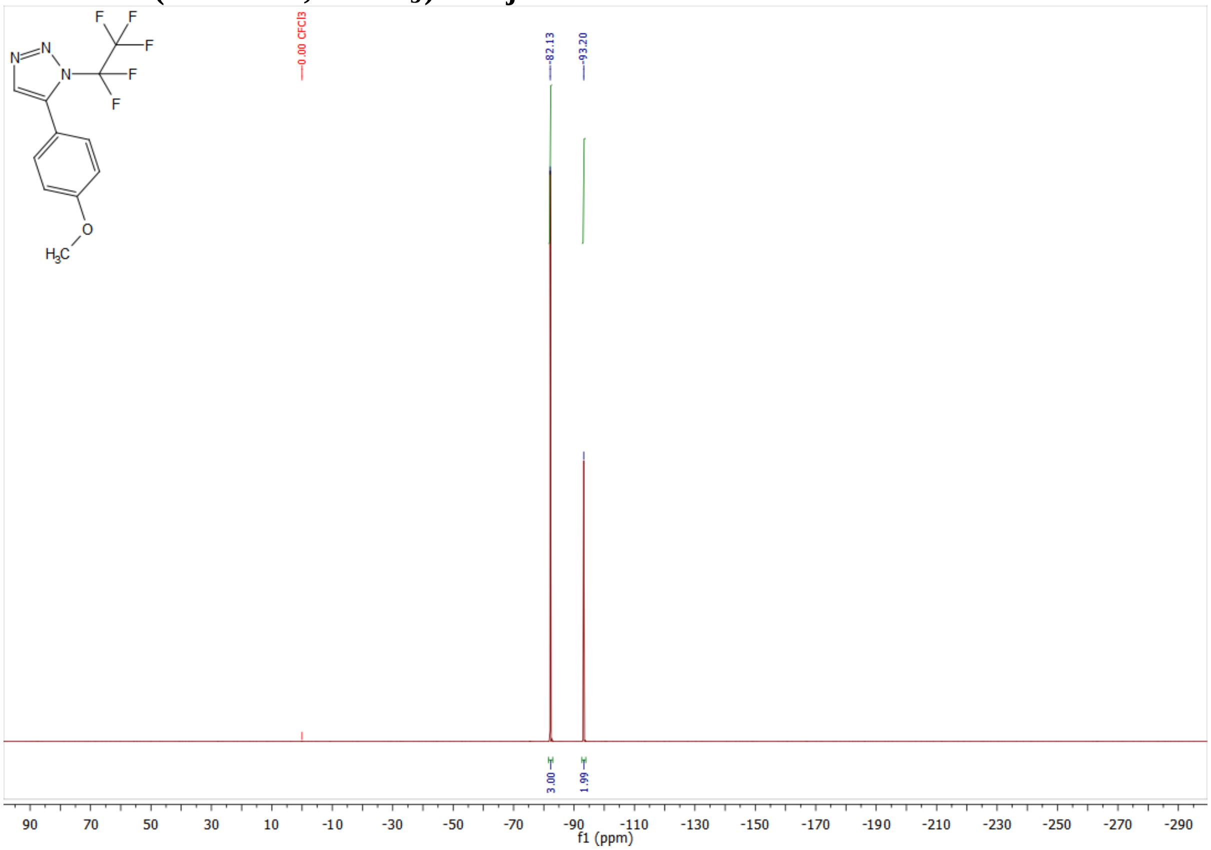

^{1}H NMR (400 MHz, $CDCl_3$) of 1k

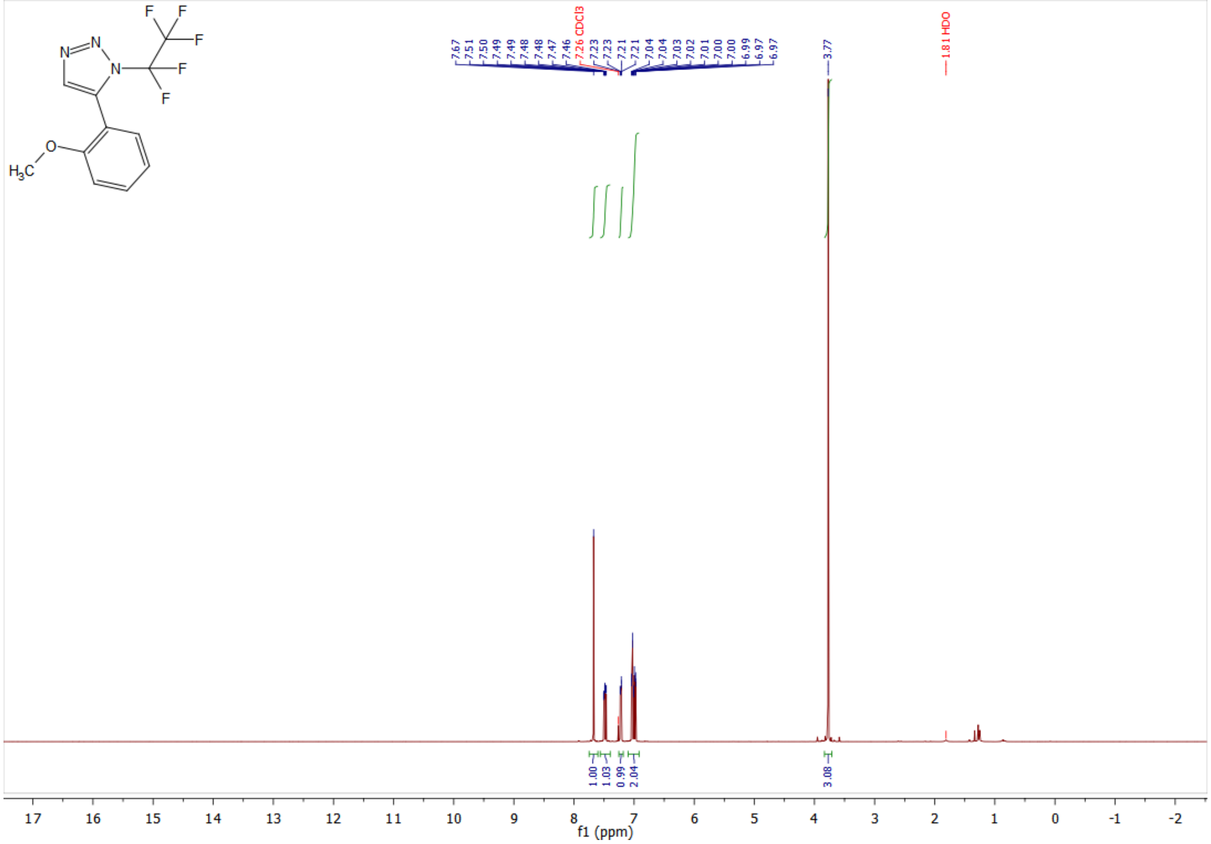

^{13}C {^{1}H} NMR (101 MHz, $CDCl_3$) of 1k

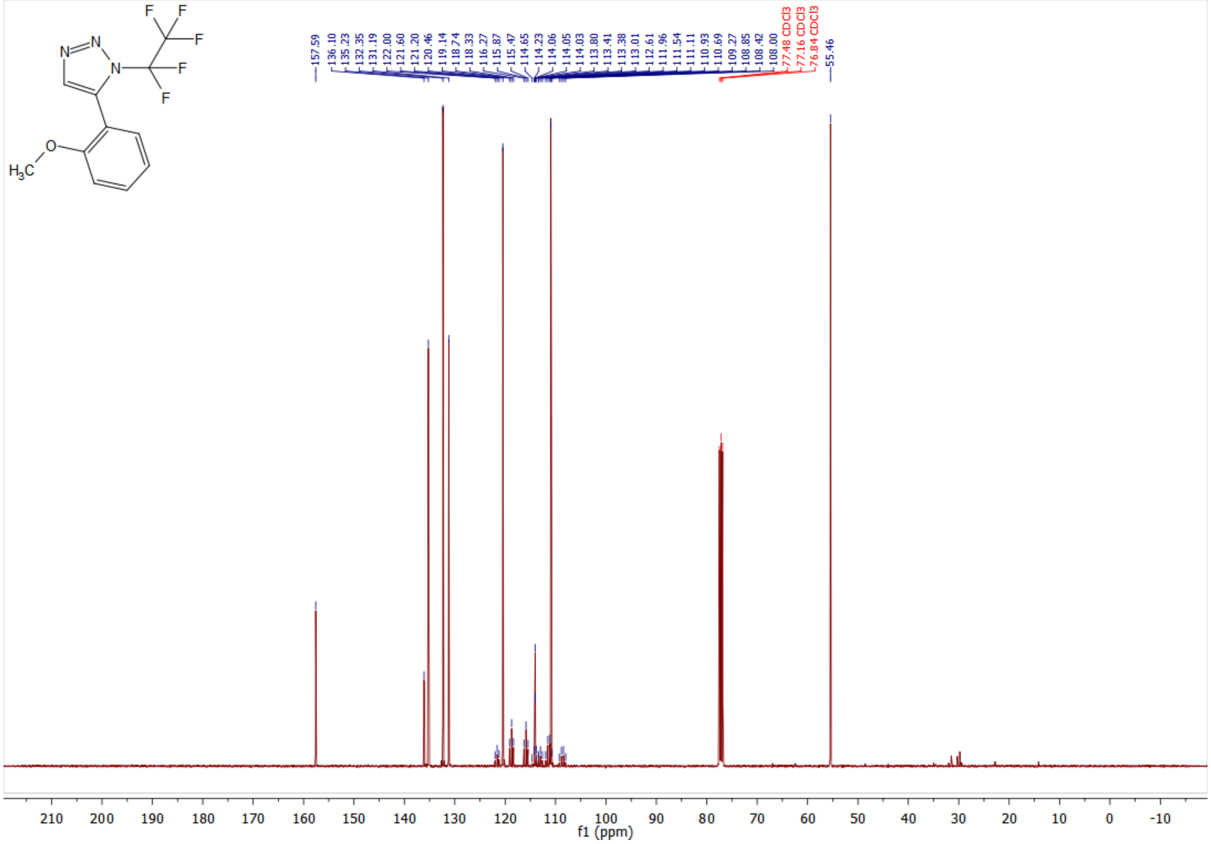

^{19}F NMR (377 MHz, $CDCl_3$) of 1k

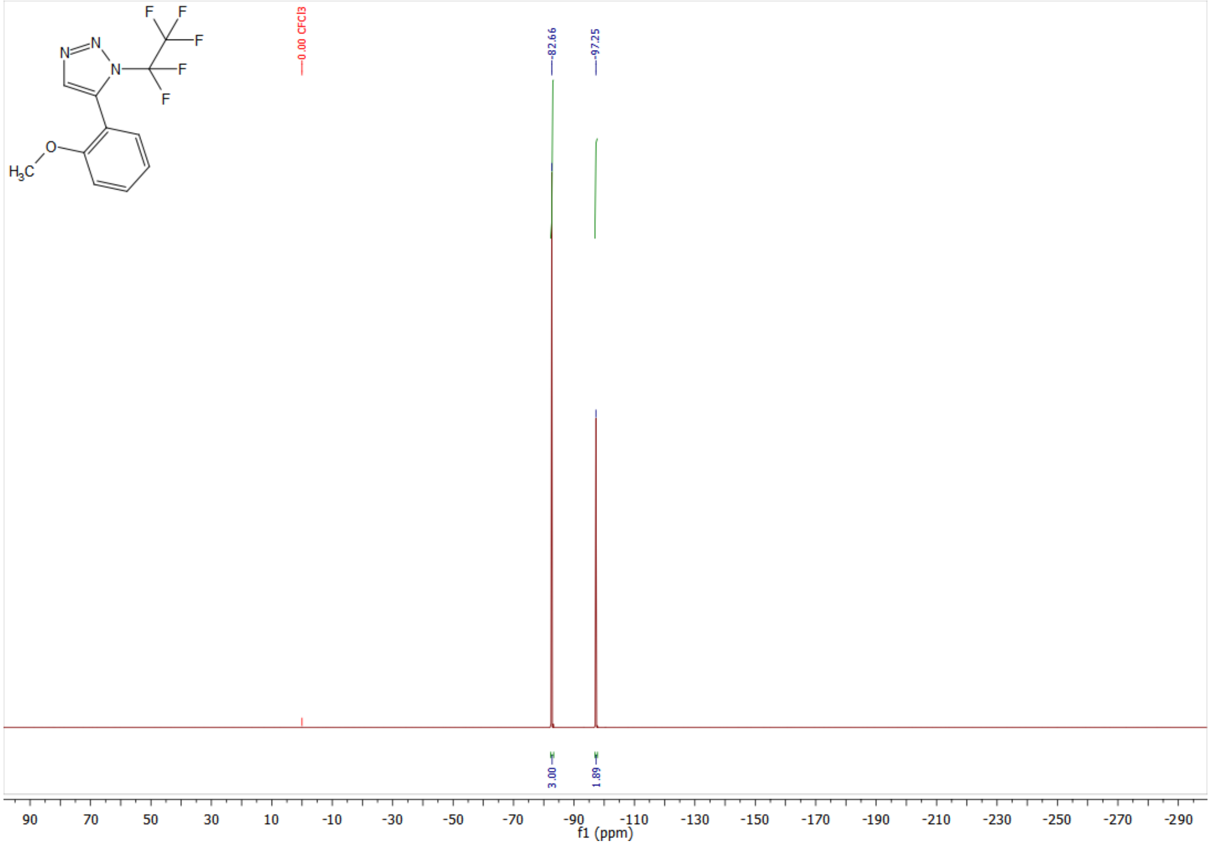

^{1}H NMR (400 MHz, $CDCl_3$) of 1l

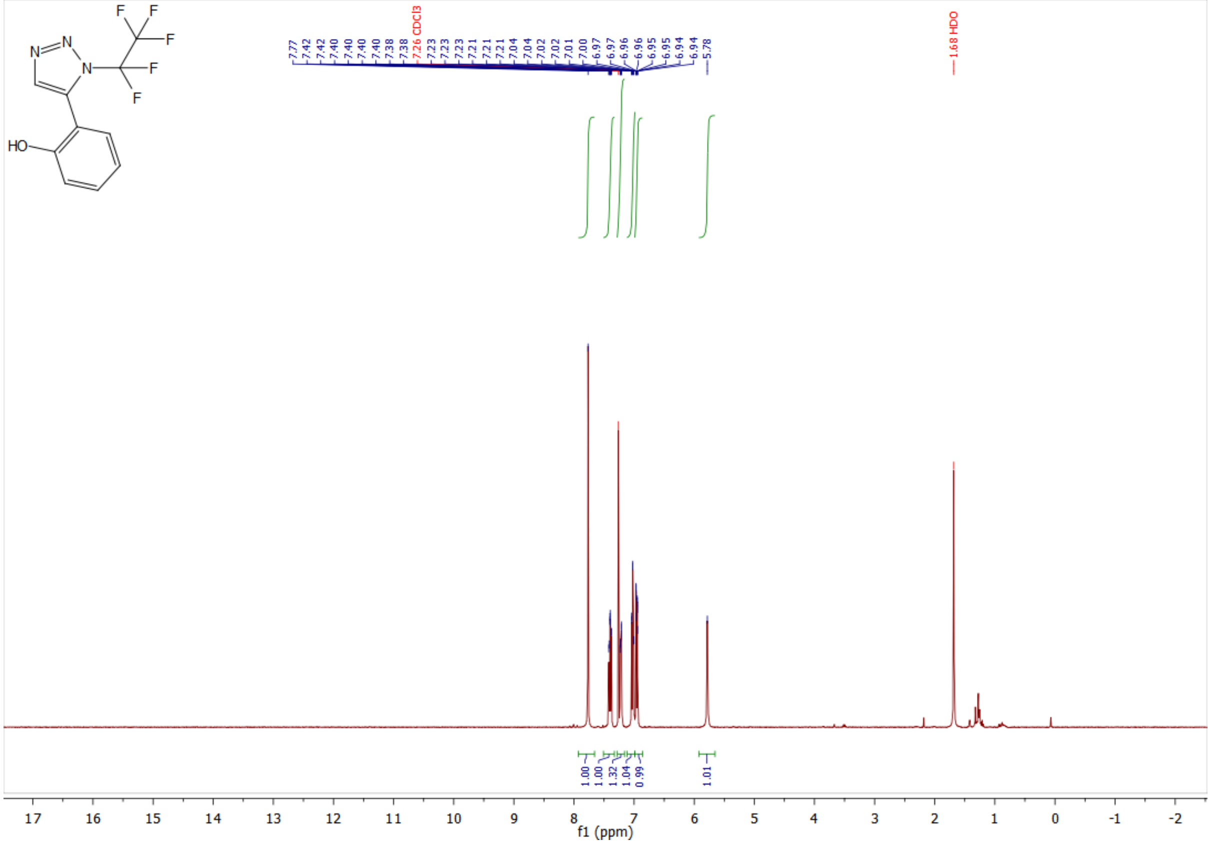

$^{13}C\{^{1}H\}$ NMR (101 MHz, $CDCl_3$) of 1l

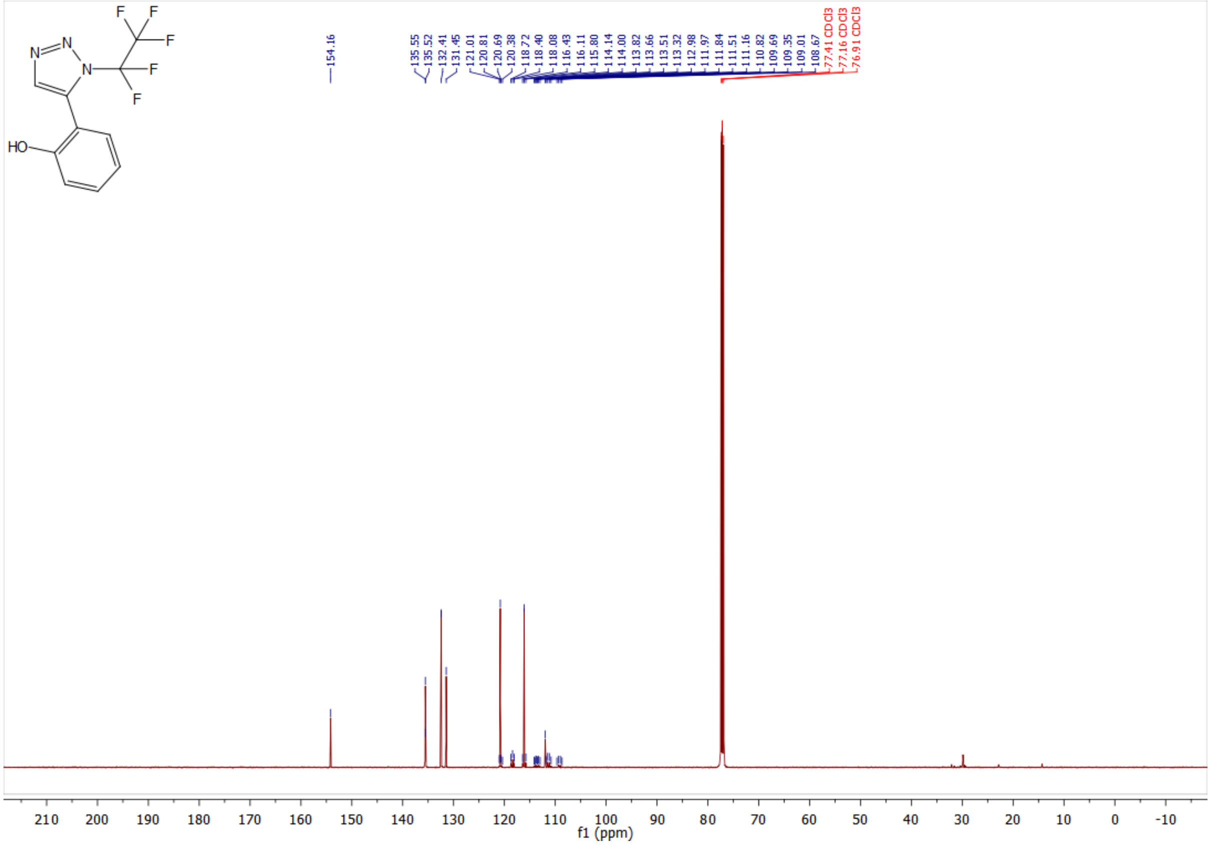

^{19}F NMR (377 MHz, $CDCl_3$) of 1l

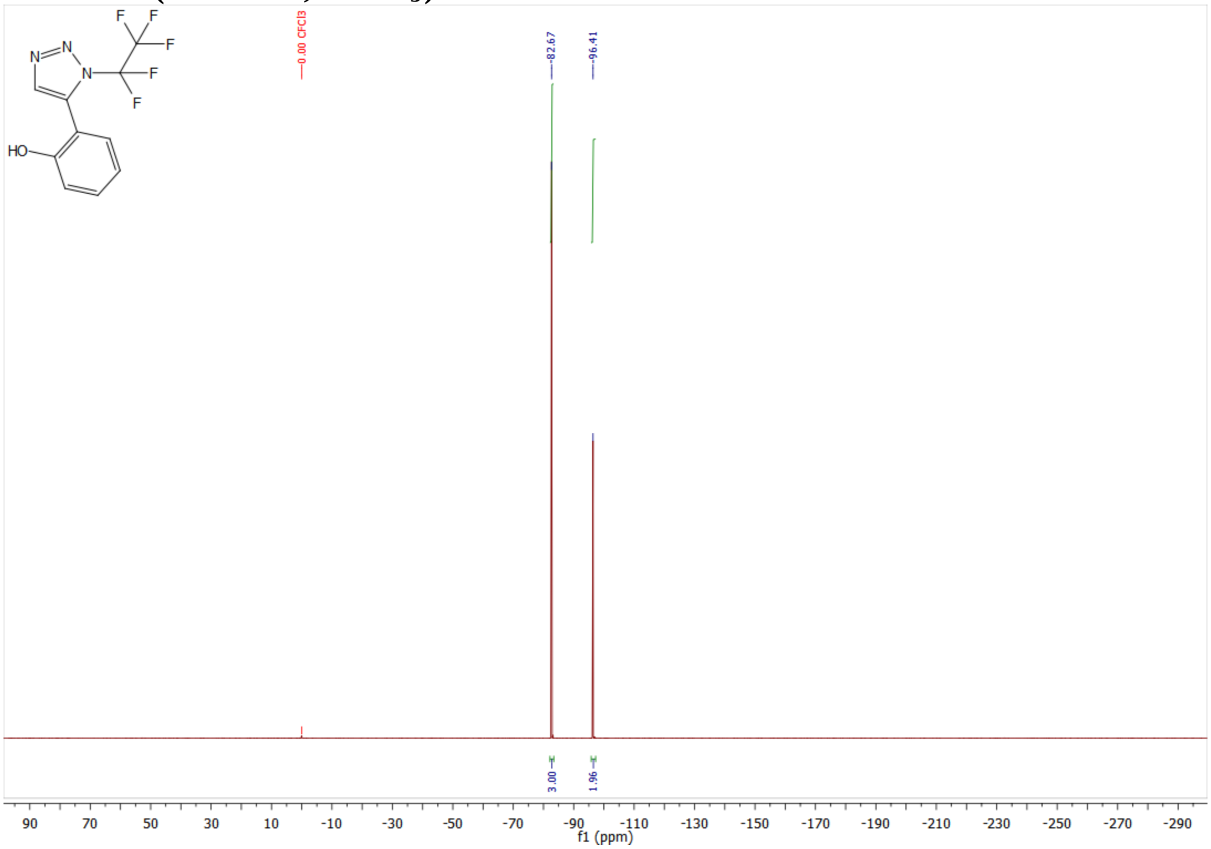

^{1}H NMR (400 MHz, $CDCl_3$) of 1m

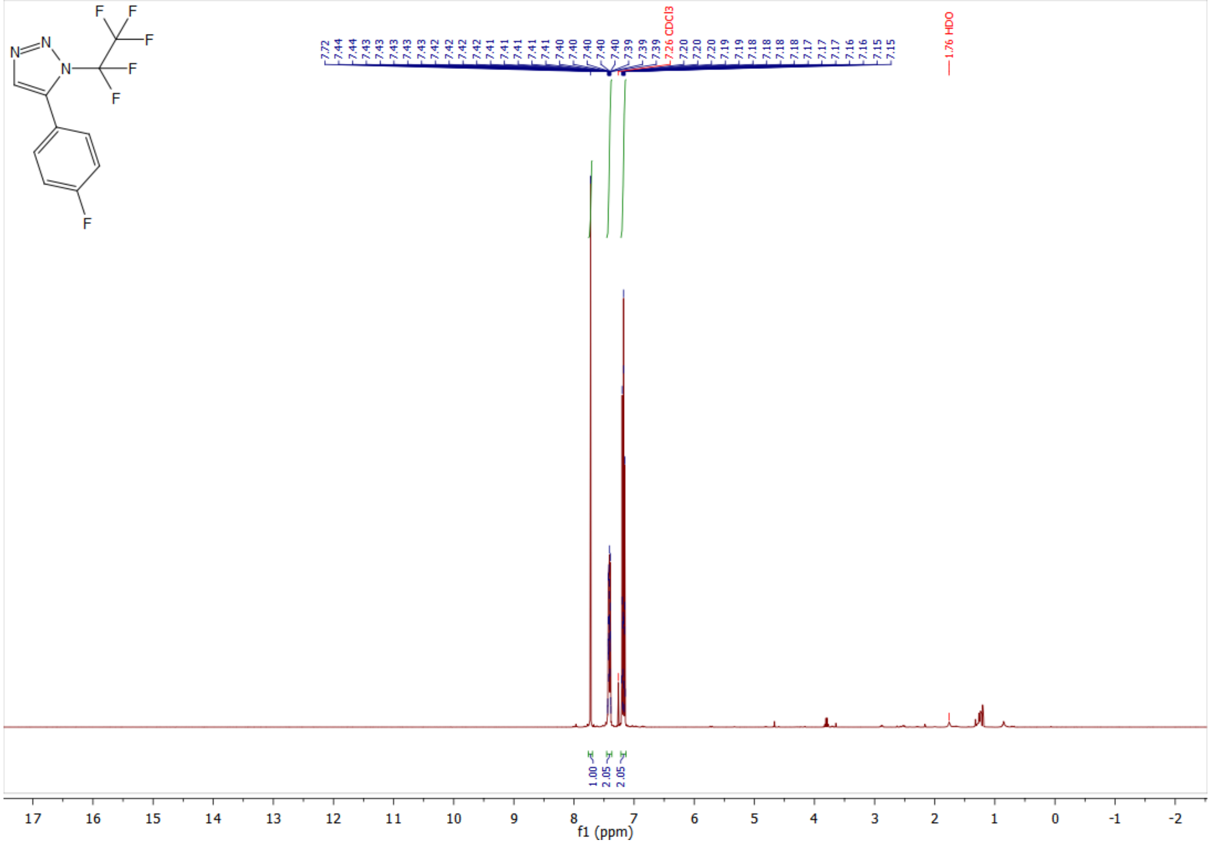

^{13}C {^{1}H} NMR (101 MHz, $CDCl_3$) of 1m

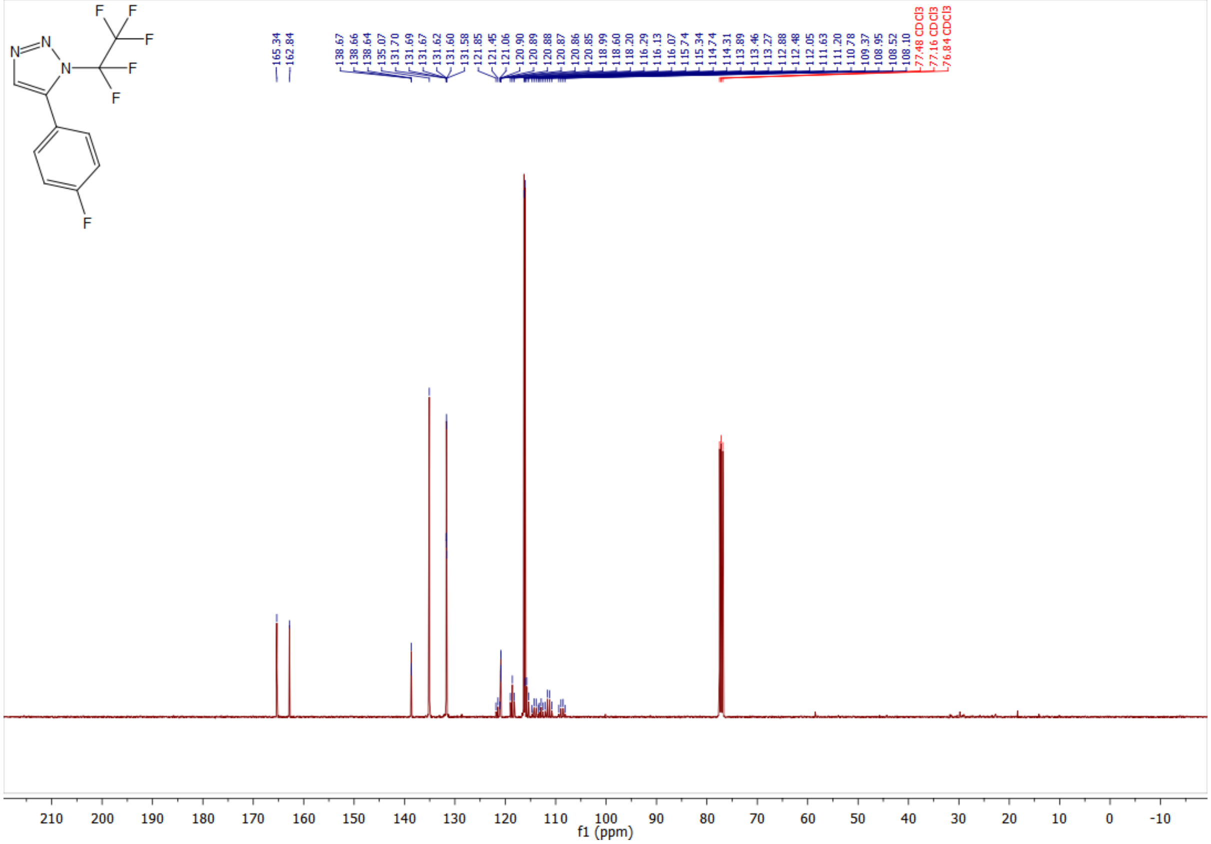

^{19}F NMR (377 MHz, $CDCl_3$) of 1m

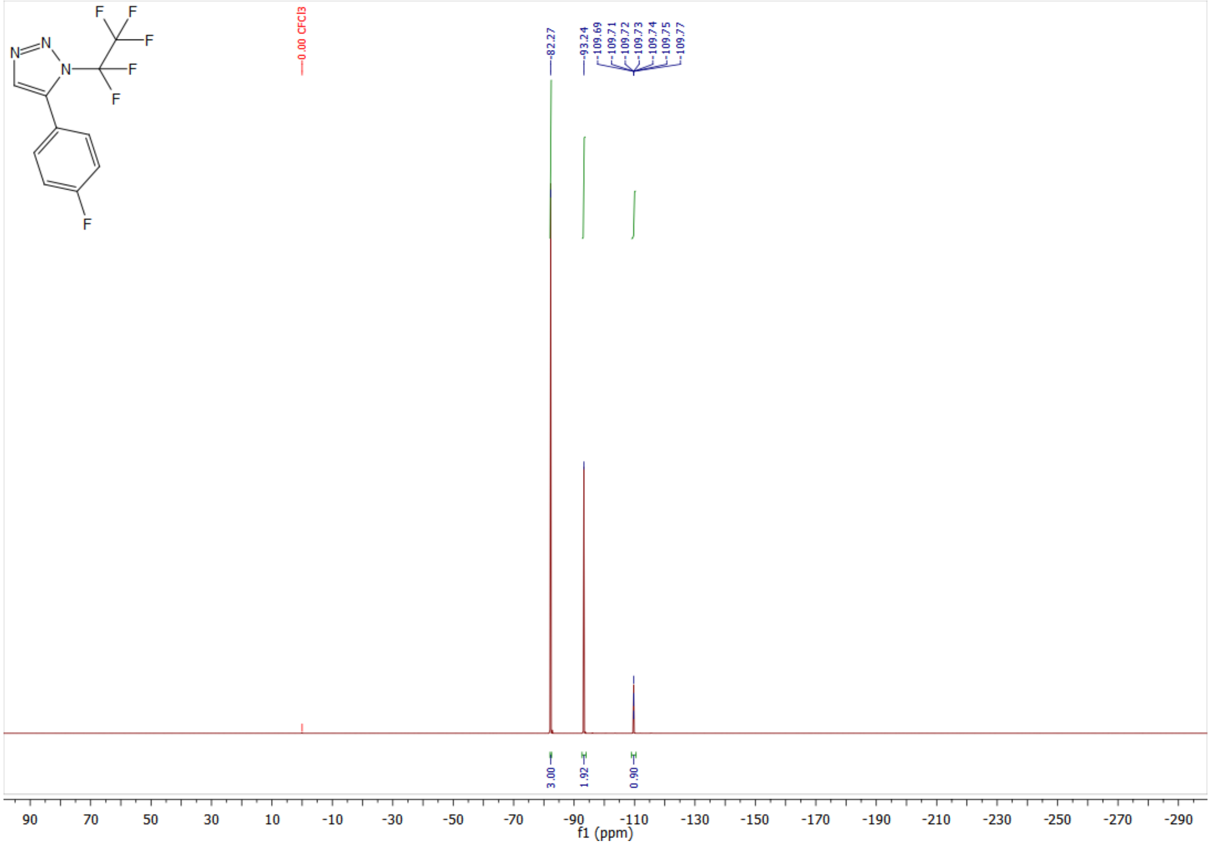

^{1}H NMR (400 MHz, $CDCl_3$) of 1n

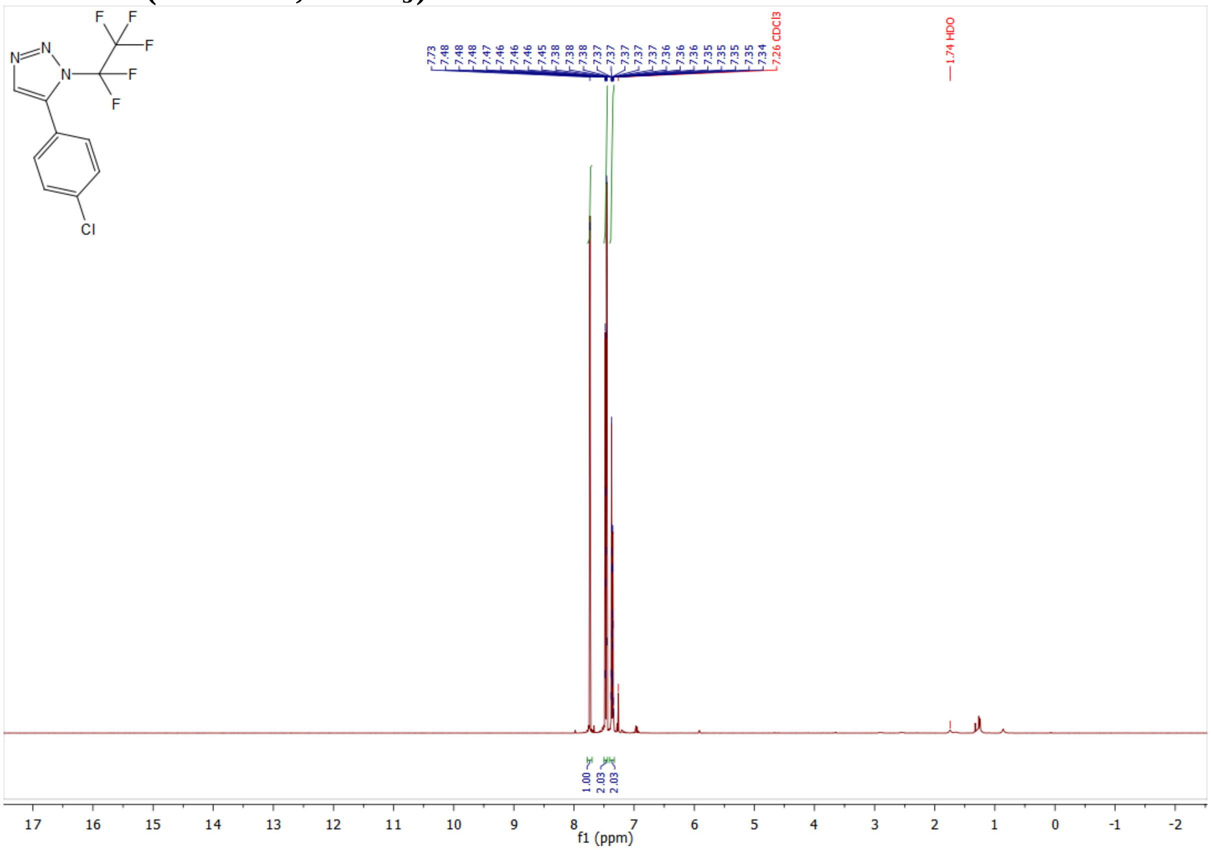

^{13}C {^{1}H} NMR (101 MHz, $CDCl_3$) of 1n

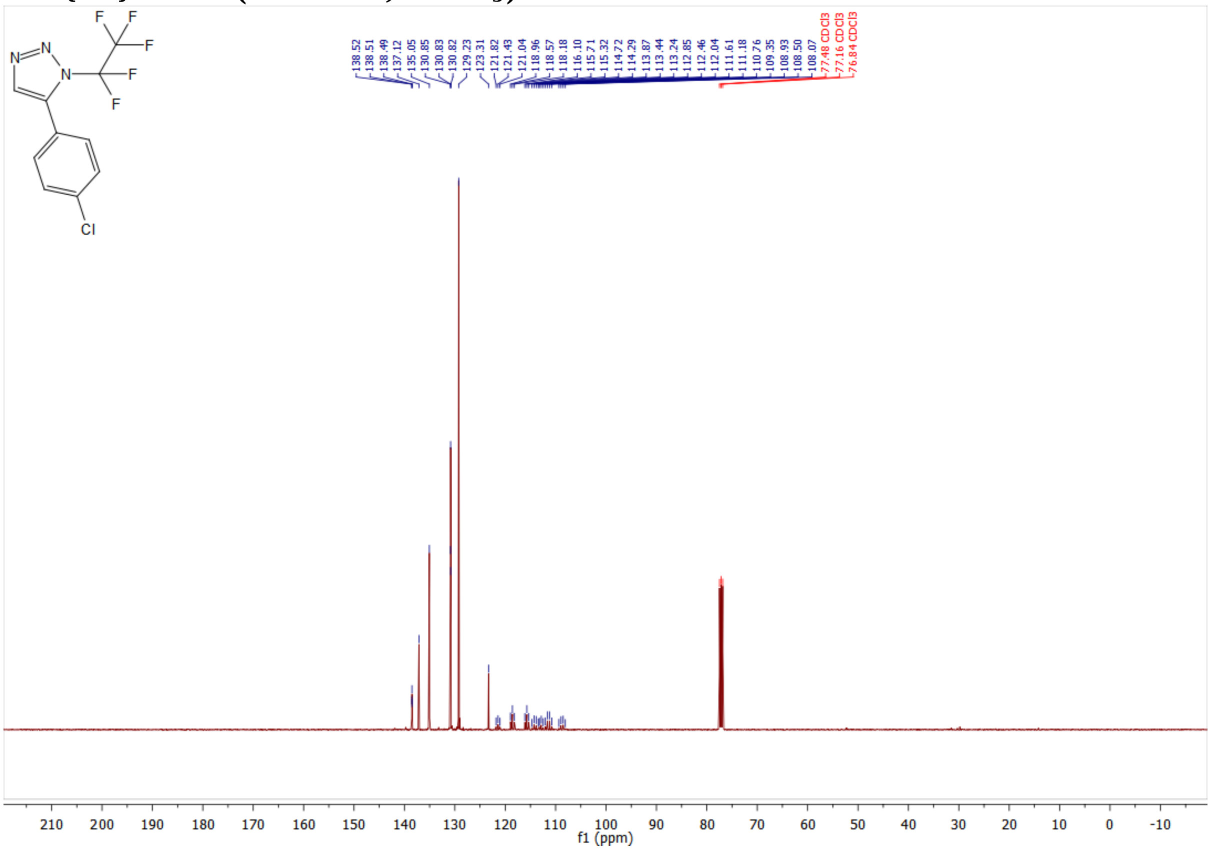

^{19}F NMR (377 MHz, $CDCl_3$) of 1n

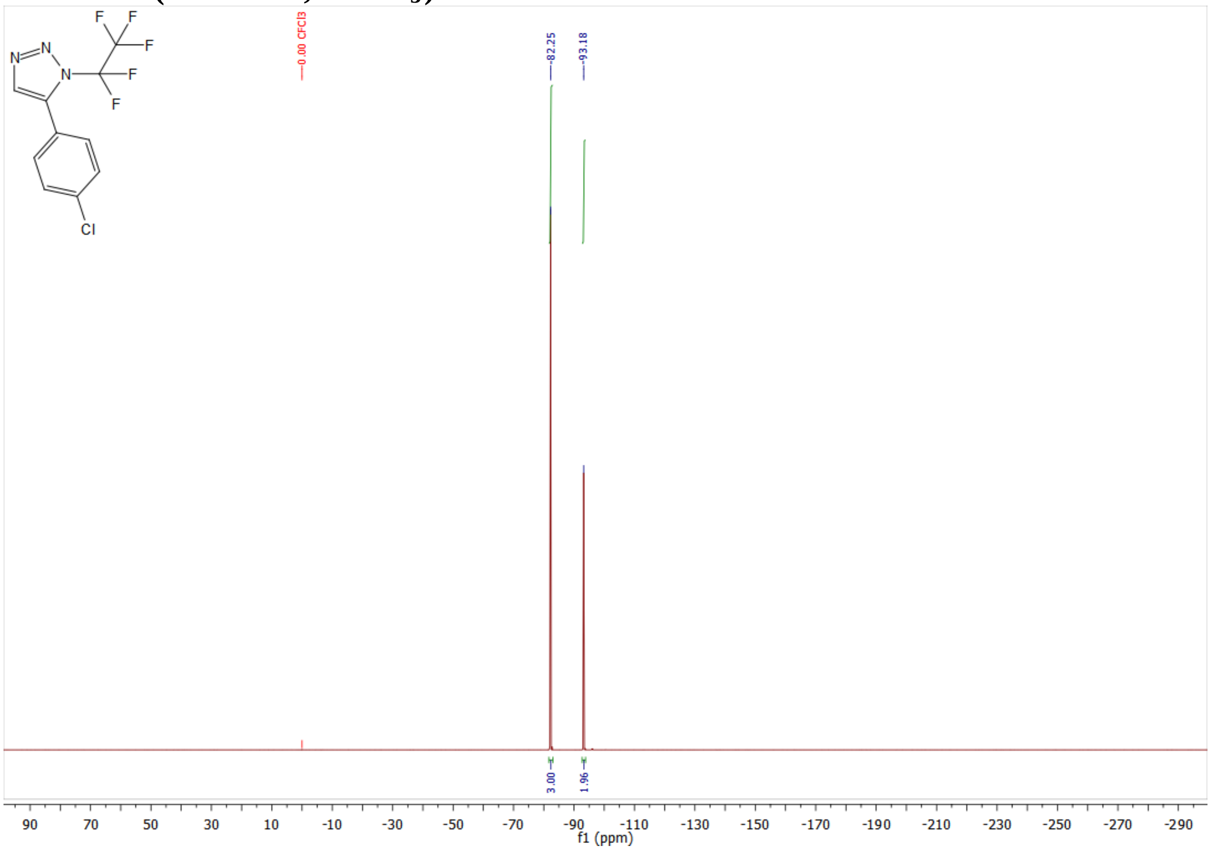

^{1}H NMR (400 MHz, $CDCl_3$) of 1o

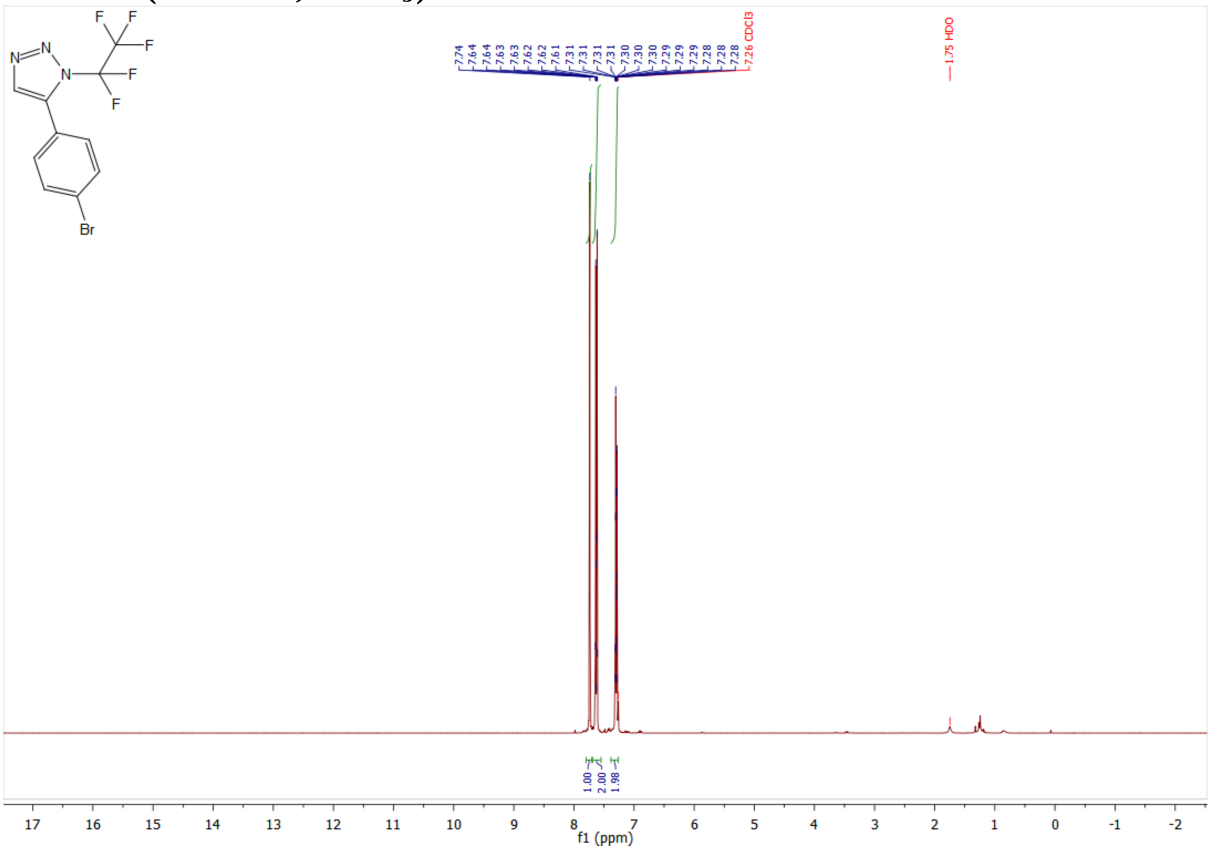

^{13}C {^{1}H} NMR (101 MHz, $CDCl_3$) of 1o

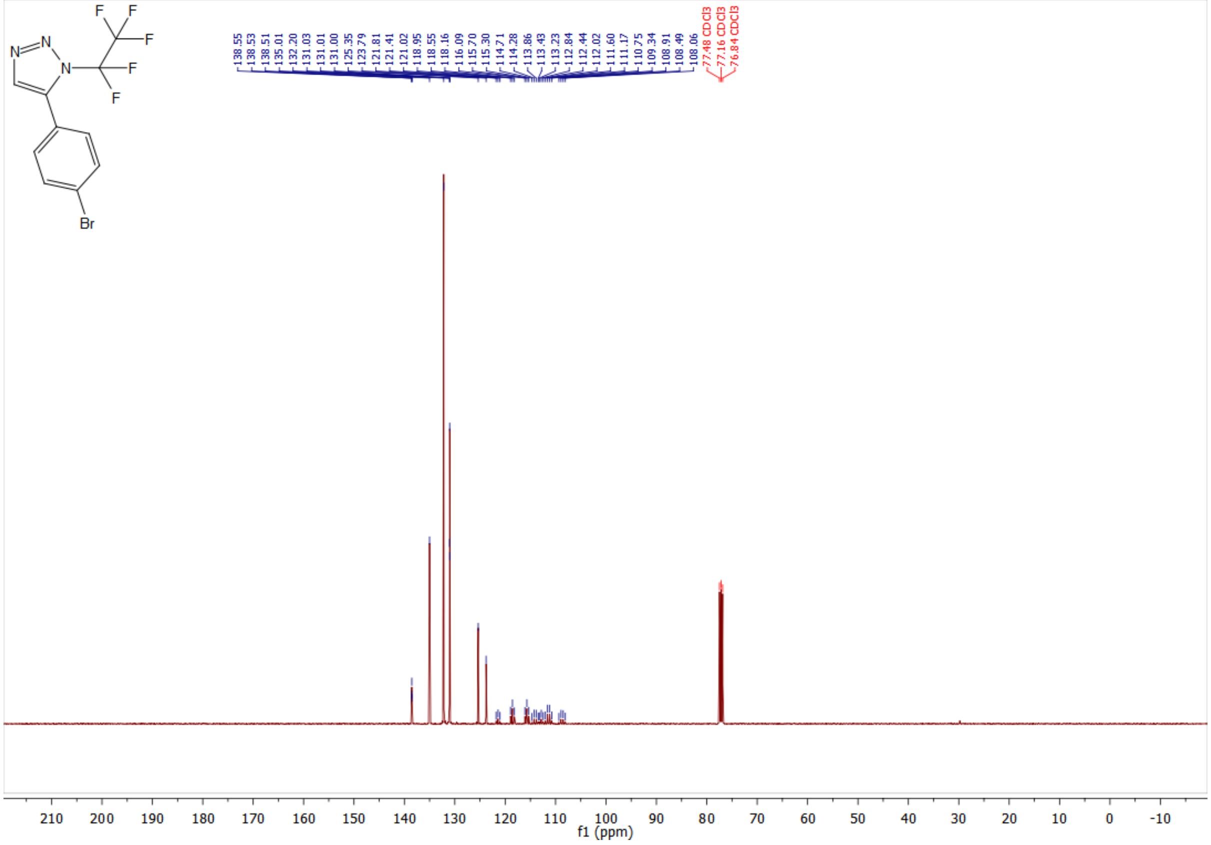

^{19}F NMR (377 MHz, $CDCl_3$) of 1o

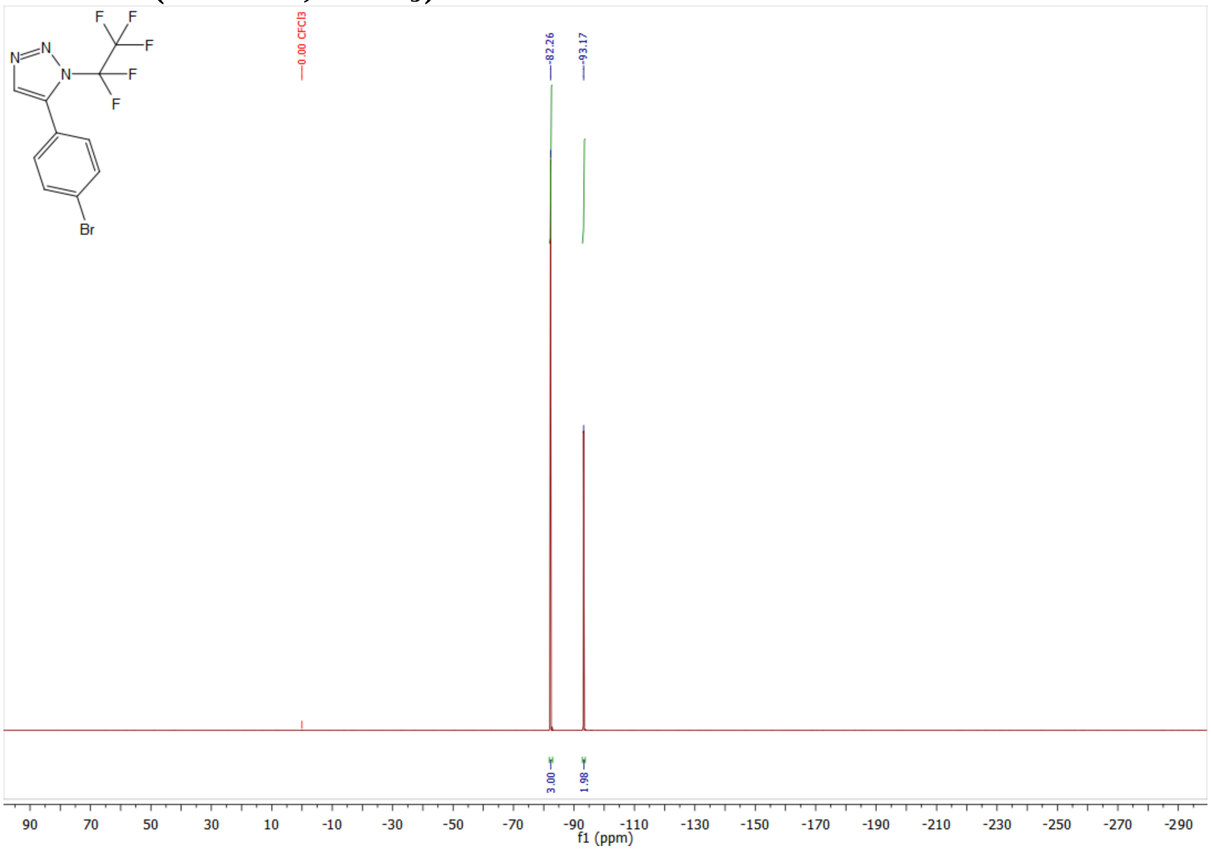

^{1}H NMR (400 MHz, $CDCl_3$) of 1p

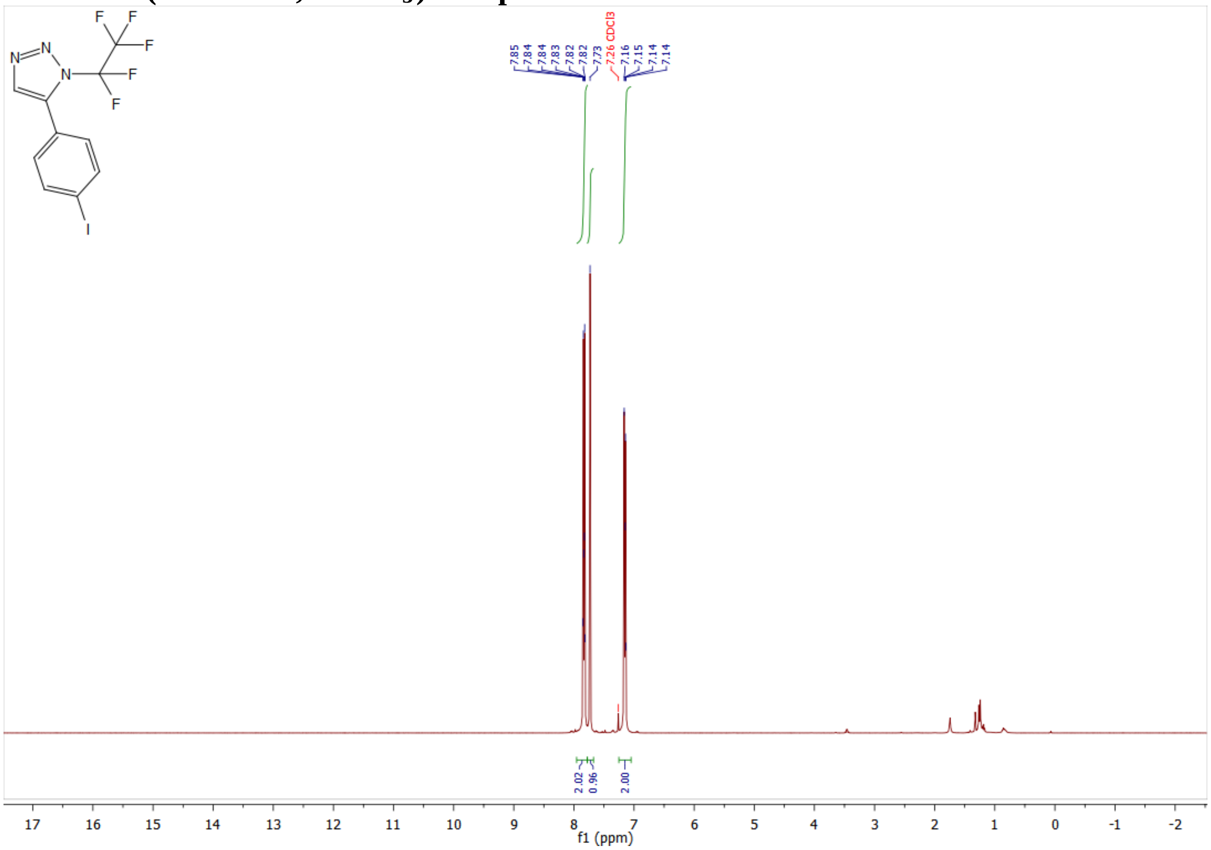

$^{13}C\{^1H\}$ NMR (101 MHz, $CDCl_3$) of 1p

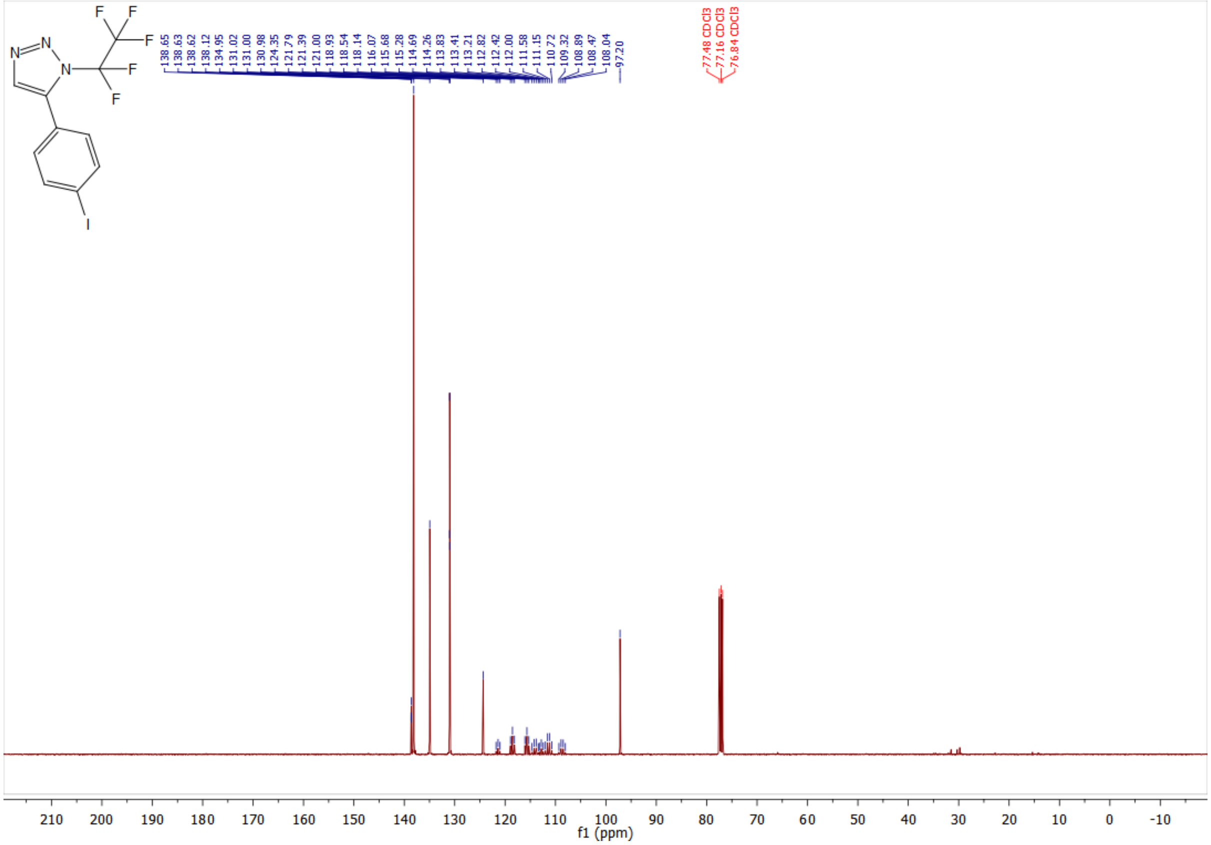

^{19}F NMR (377 MHz, $CDCl_3$) of 1p

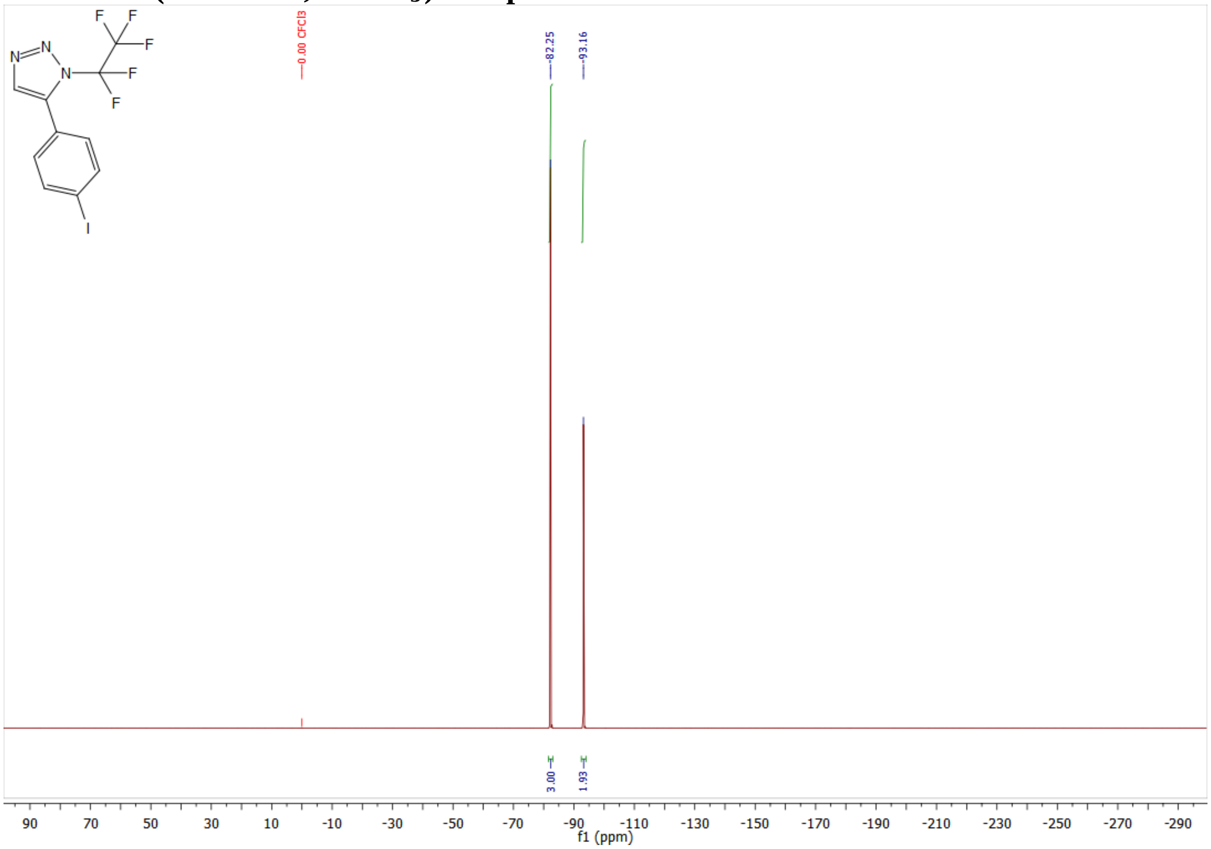

^{1}H NMR (400 MHz, $CDCl_3$) of 1q

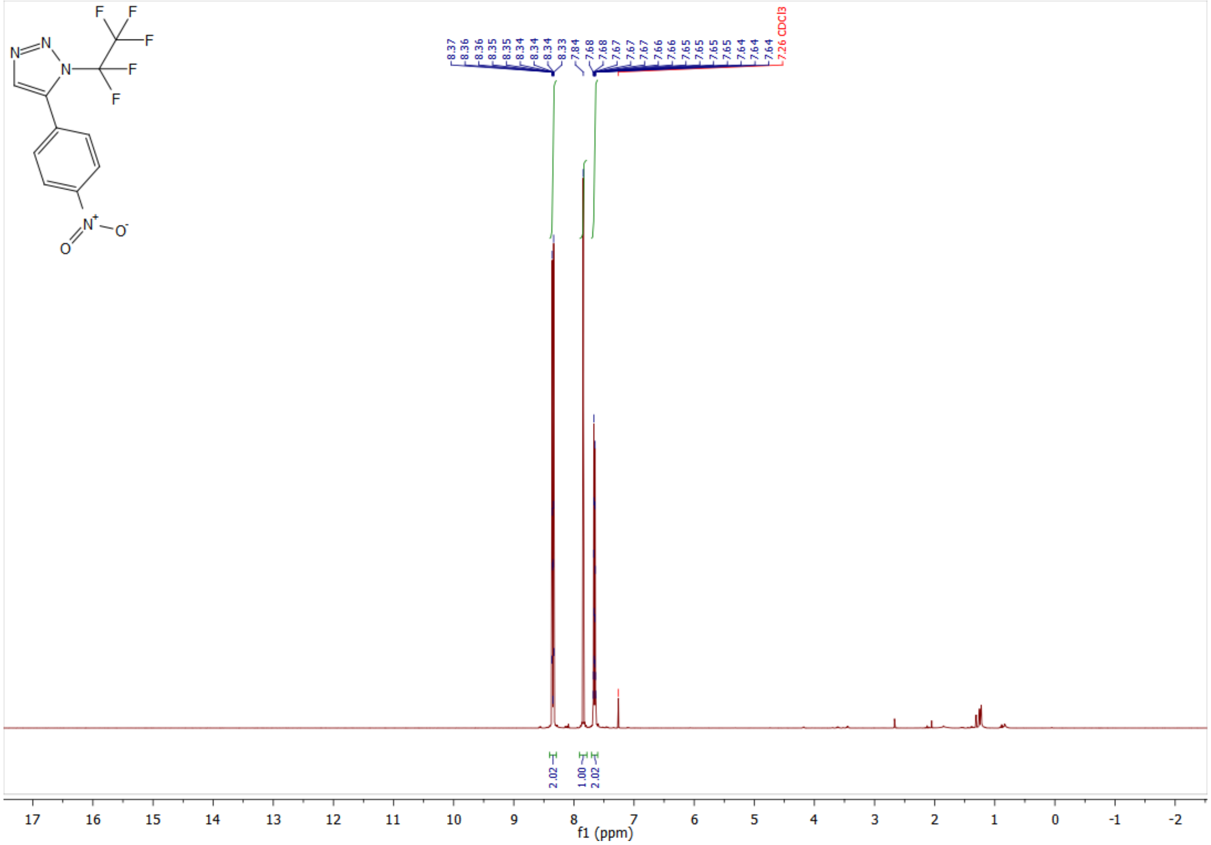

^{13}C {^{1}H} NMR (101 MHz, $CDCl_3$) of 1q

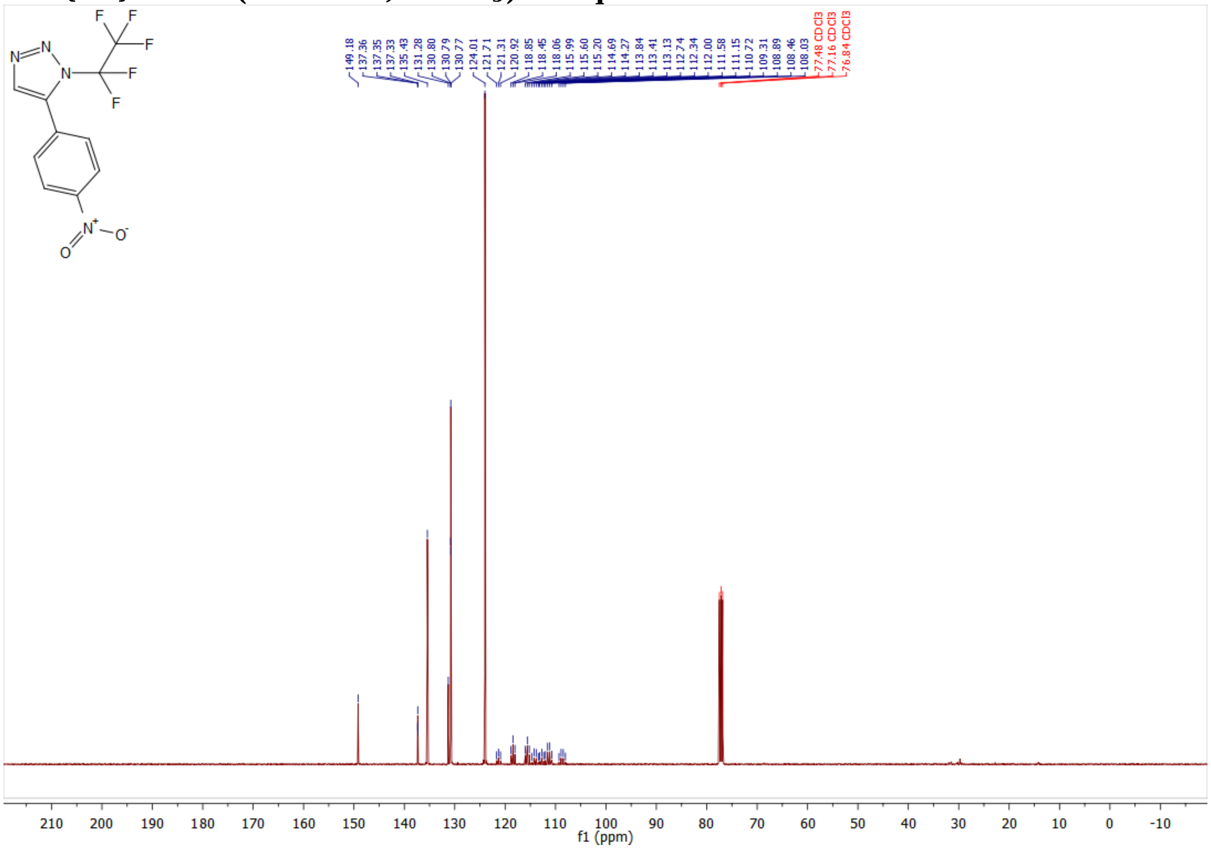

^{19}F NMR (377 MHz, $CDCl_3$) of 1q

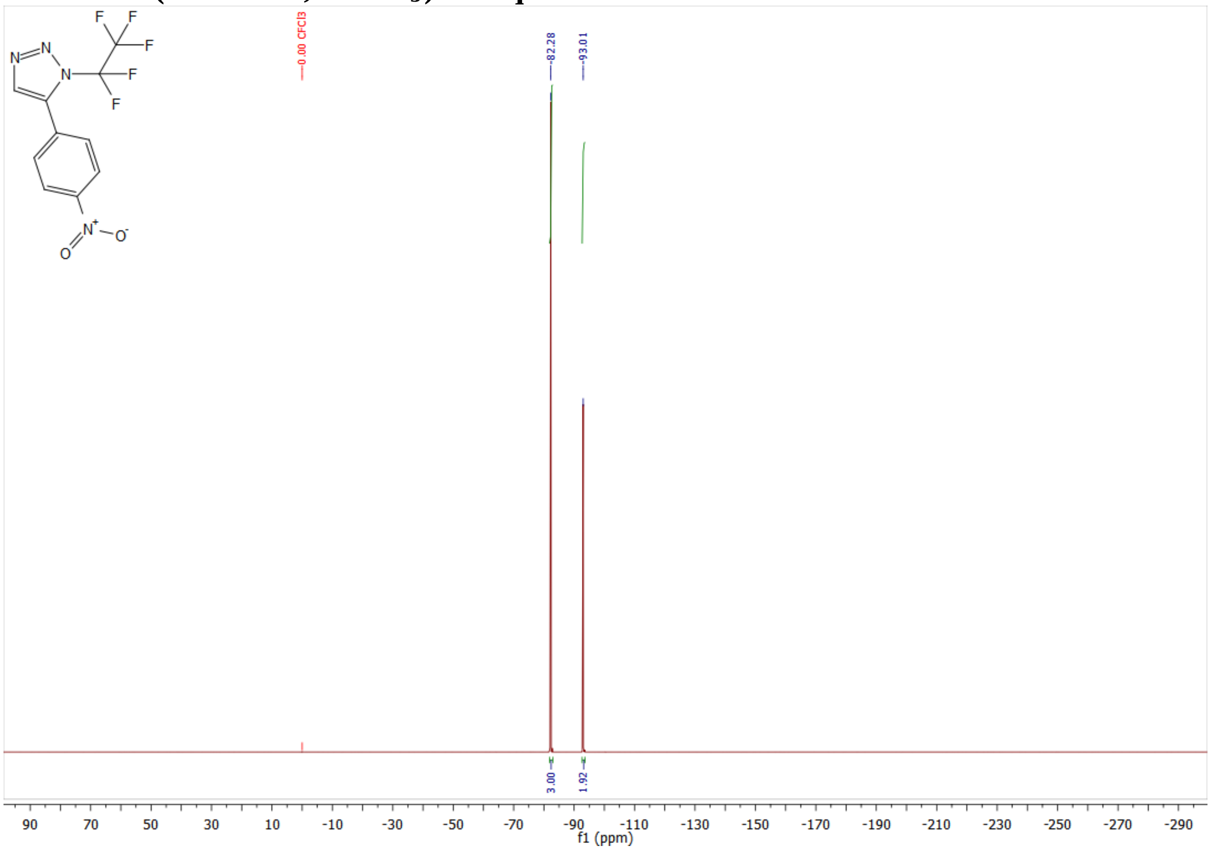

^{1}H NMR (400 MHz, $CDCl_3$) of 1r

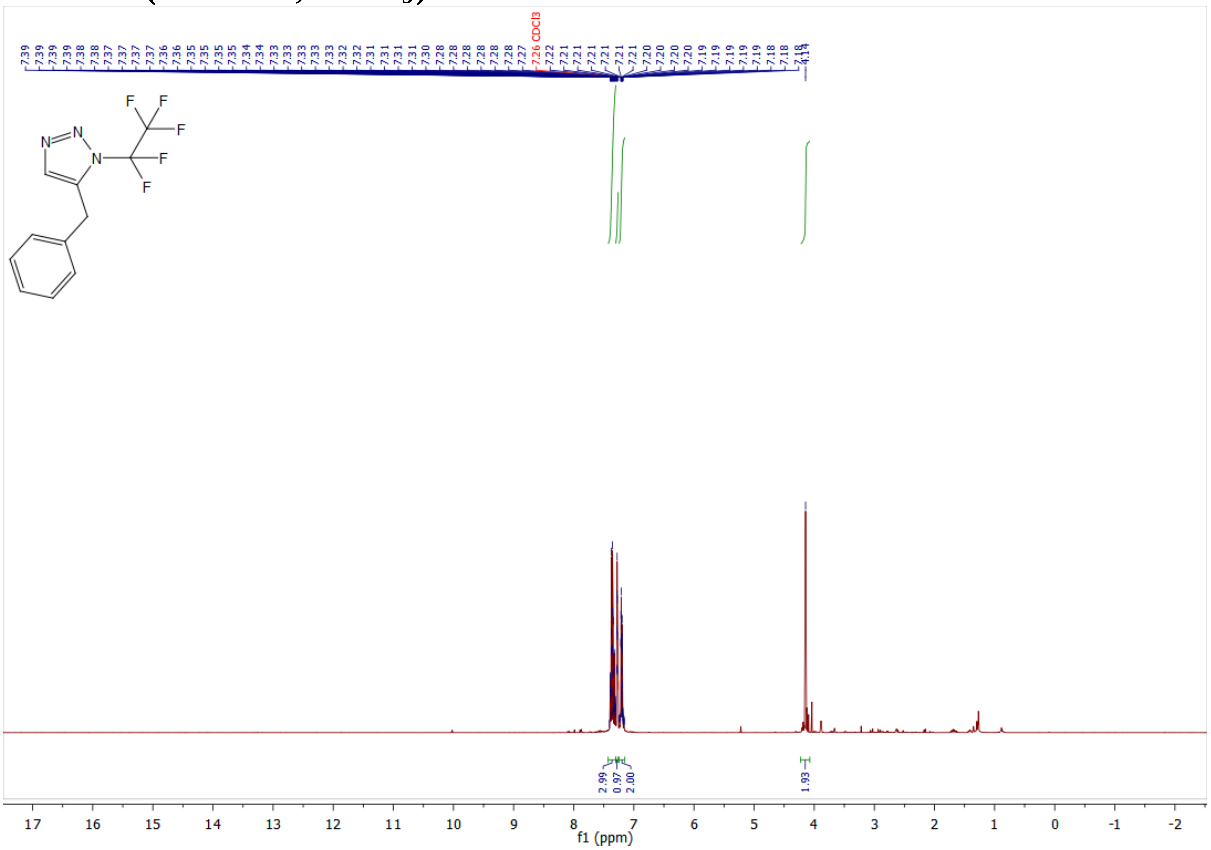

$^{13}C\ \{^1H\}$ NMR (101 MHz, $CDCl_3$) of 1r

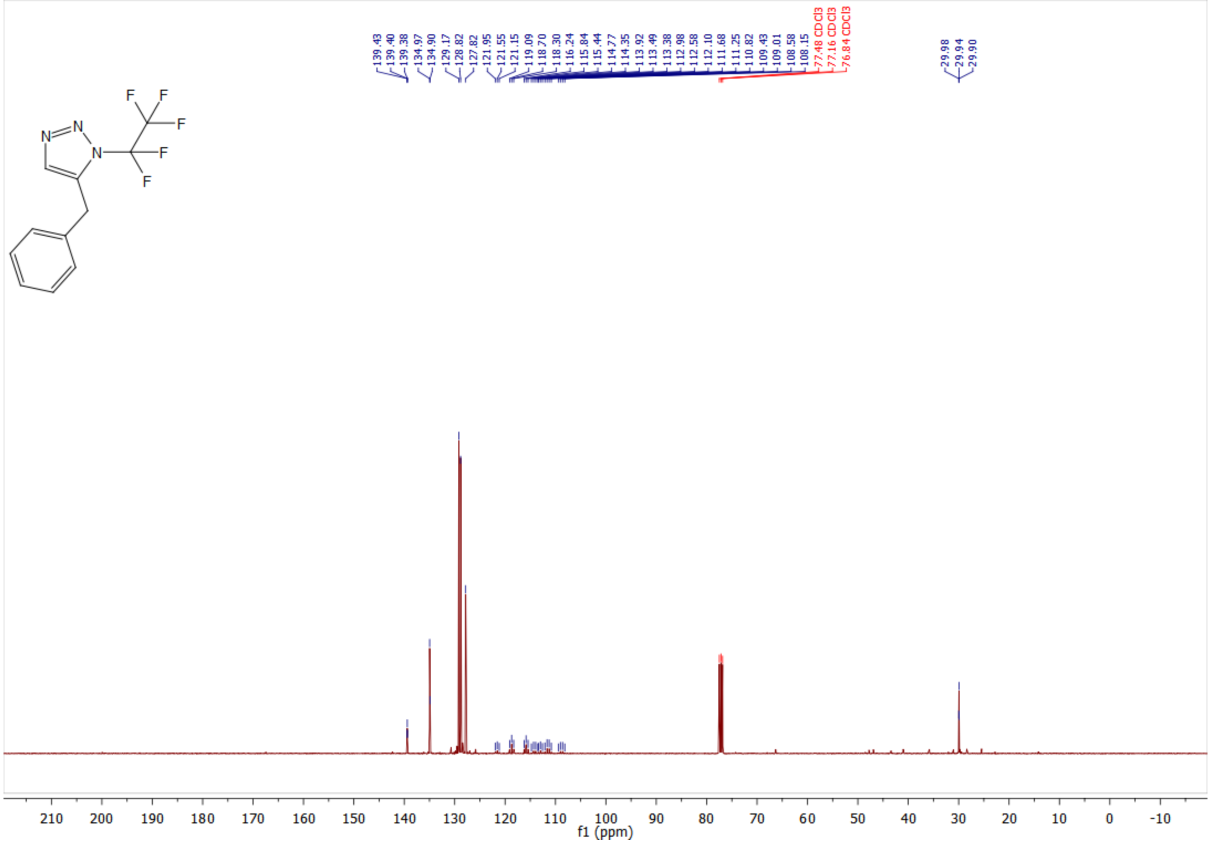

^{19}F NMR (377 MHz, $CDCl_3$) of 1r

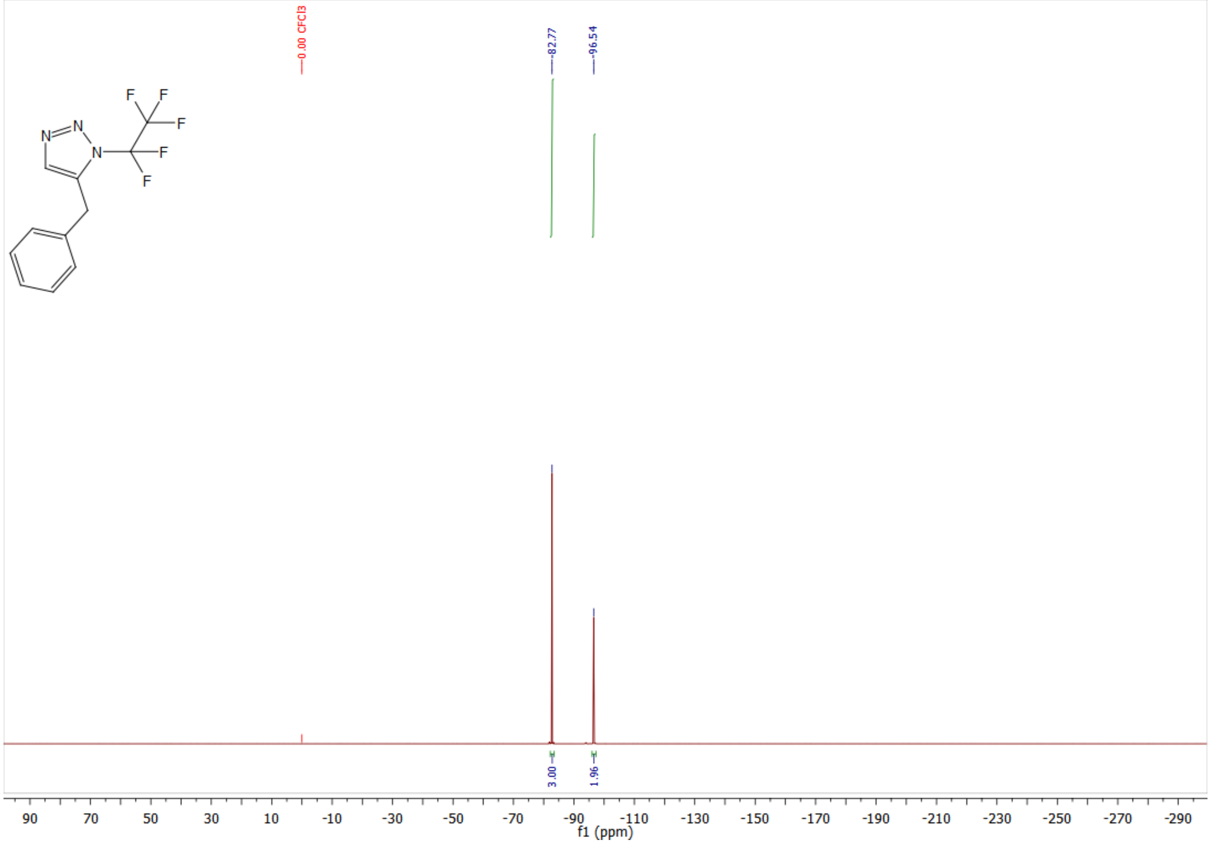

^{1}H NMR (400 MHz, $CDCl_3$) of 1s

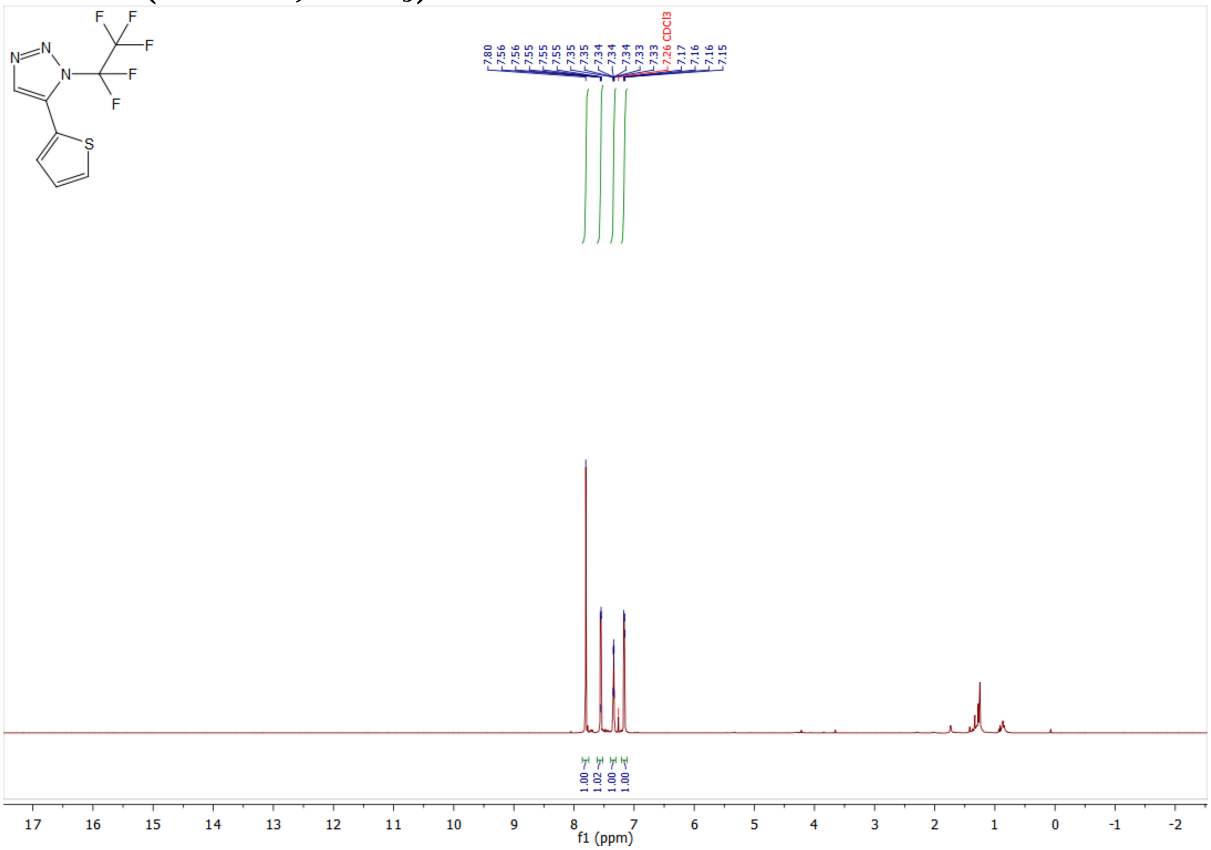

^{13}C {^{1}H} NMR (101 MHz, $CDCl_3$) of 1s

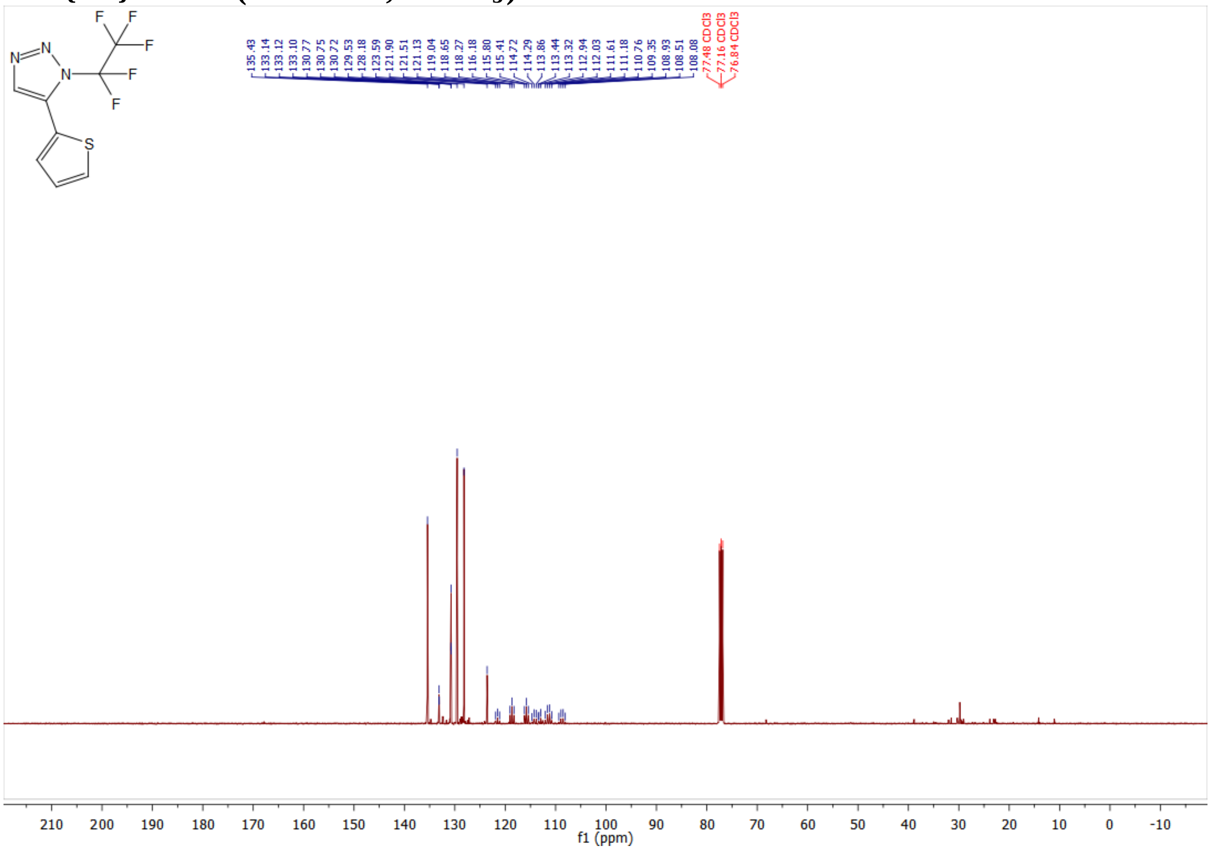

^{19}F NMR (377 MHz, $CDCl_3$) of 1s

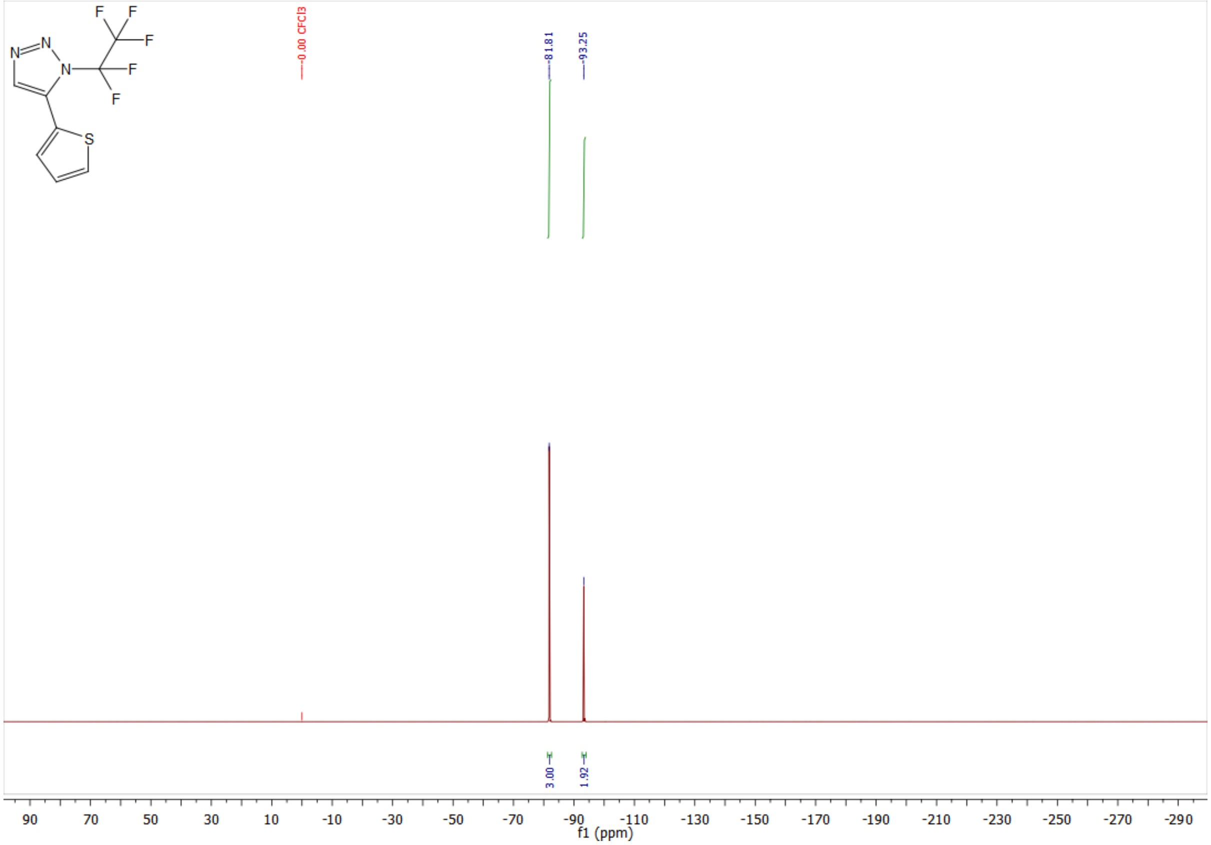

^{1}H NMR (400 MHz, $CDCl_3$) of 1t

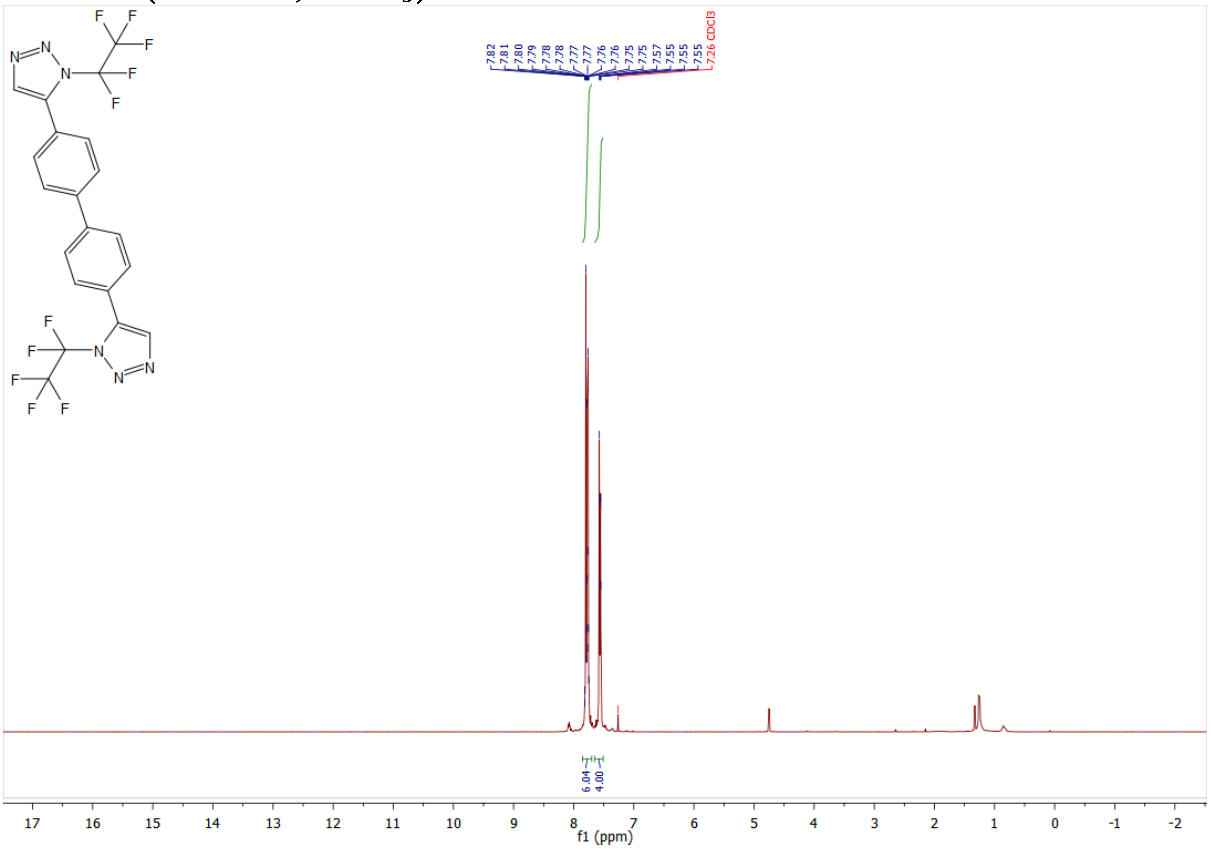

$^{13}C\ \{^{1}H\}$ NMR (101 MHz, $CDCl_3$) of 1t

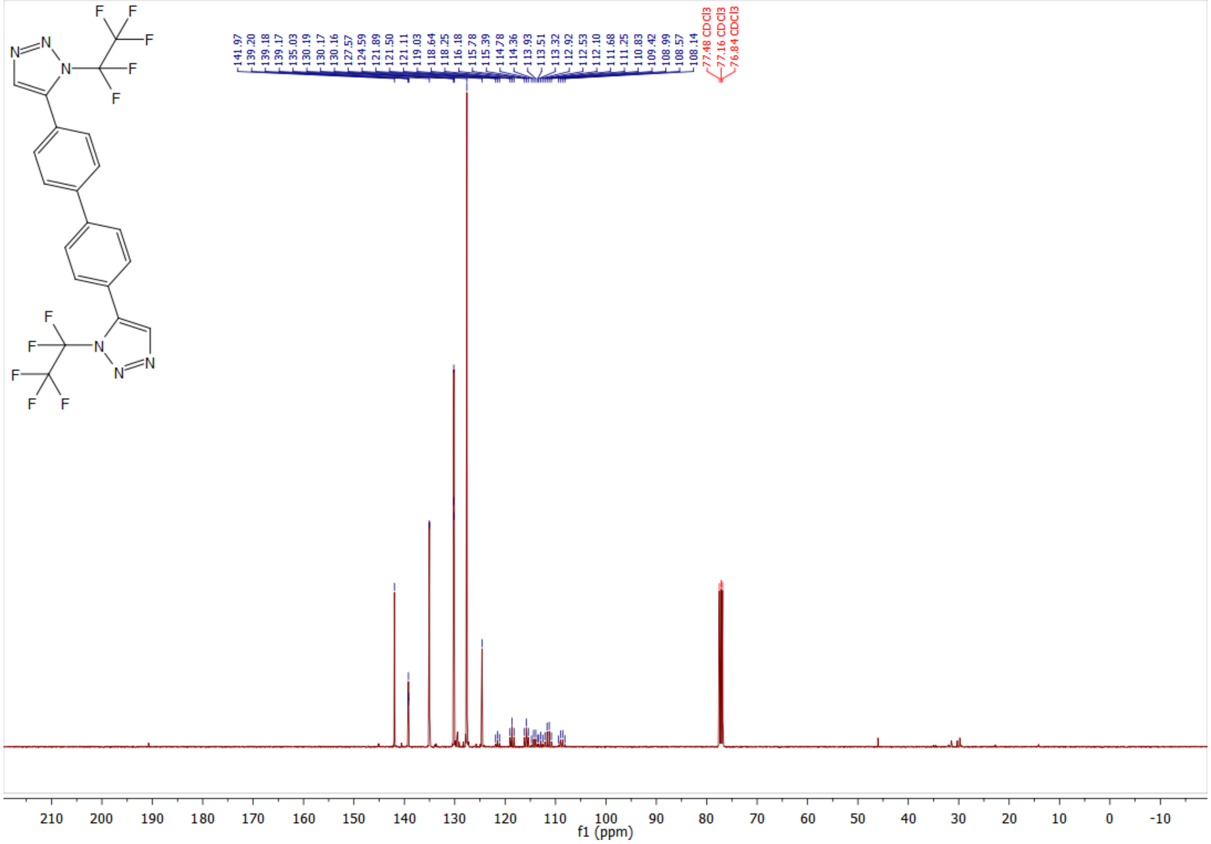

^{19}F NMR (377 MHz, $CDCl_3$) of 1t

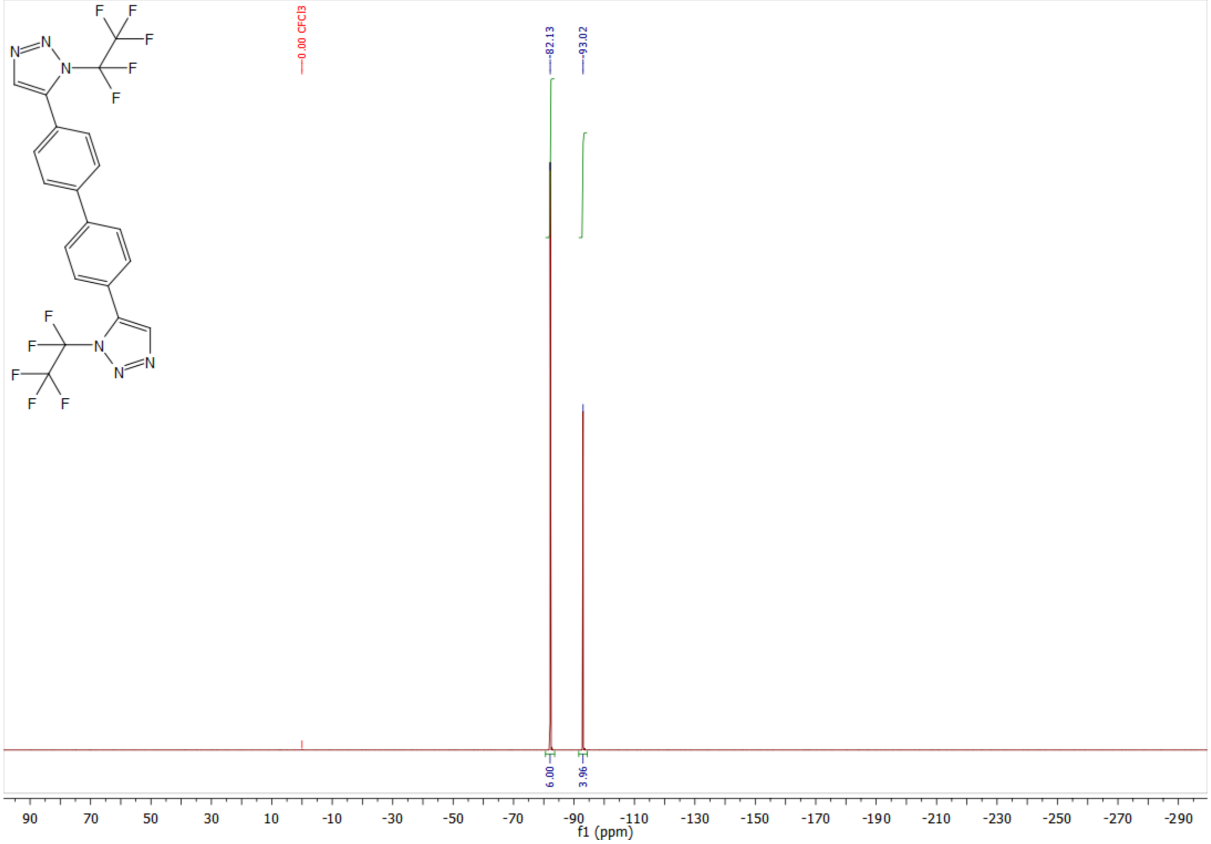

^{1}H NMR (400 MHz, $CDCl_3$) of 1v

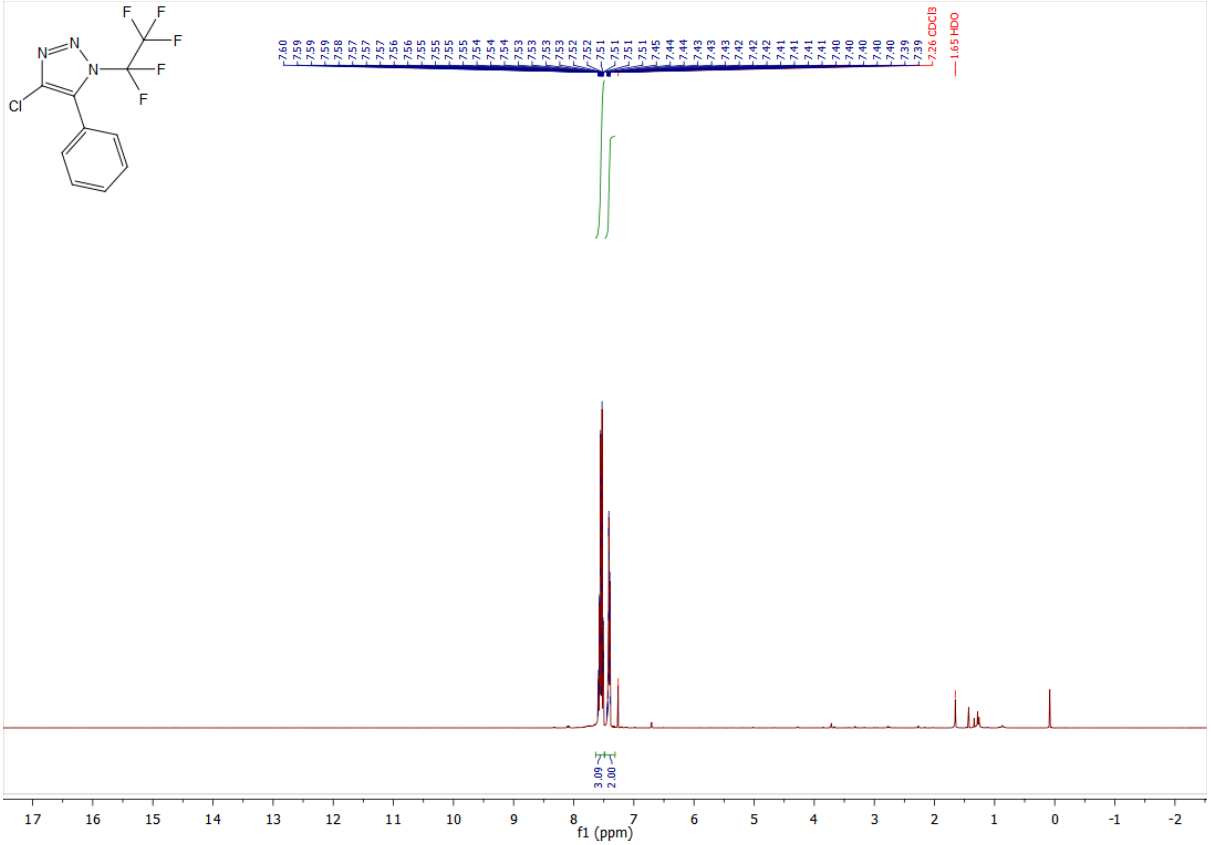

$^{13}C\{^{1}H\}$ NMR (101 MHz, $CDCl_3$) of 1v

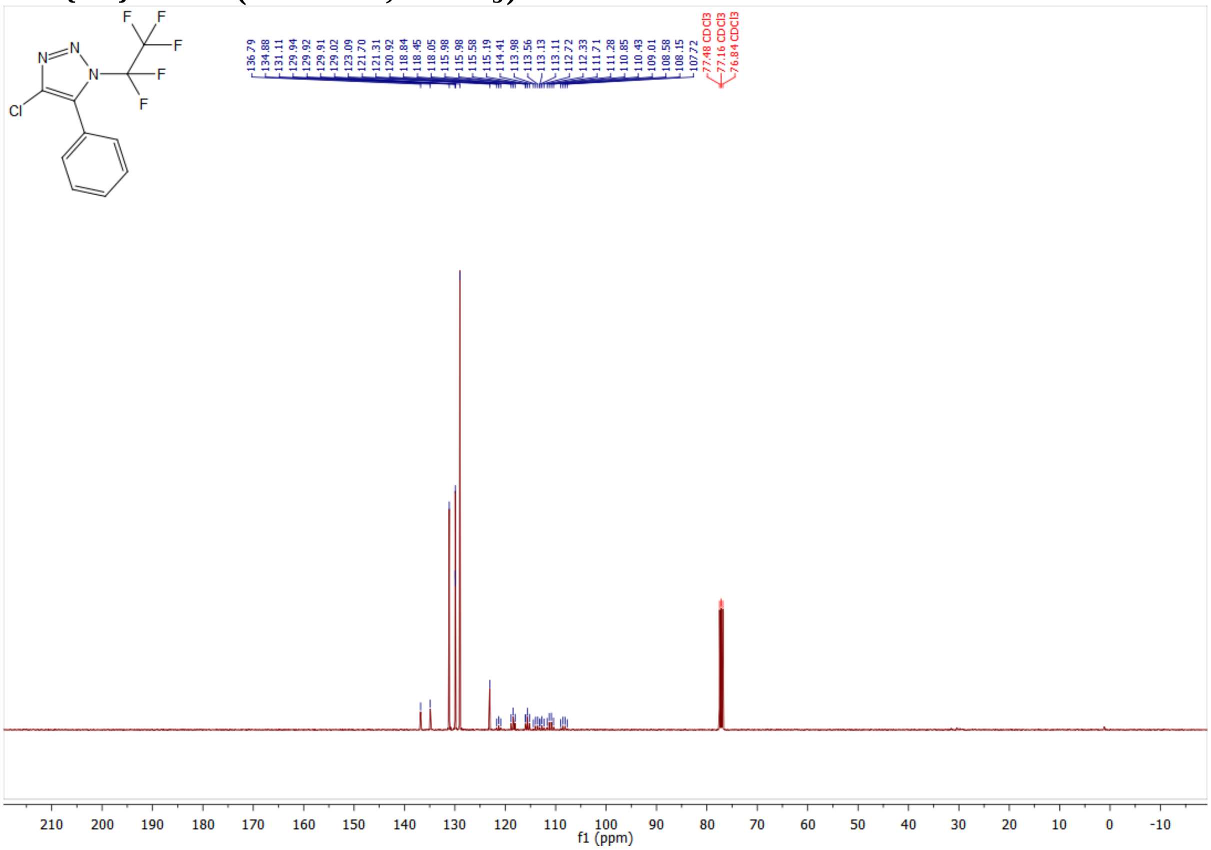

^{19}F NMR (377 MHz, $CDCl_3$) of 1v

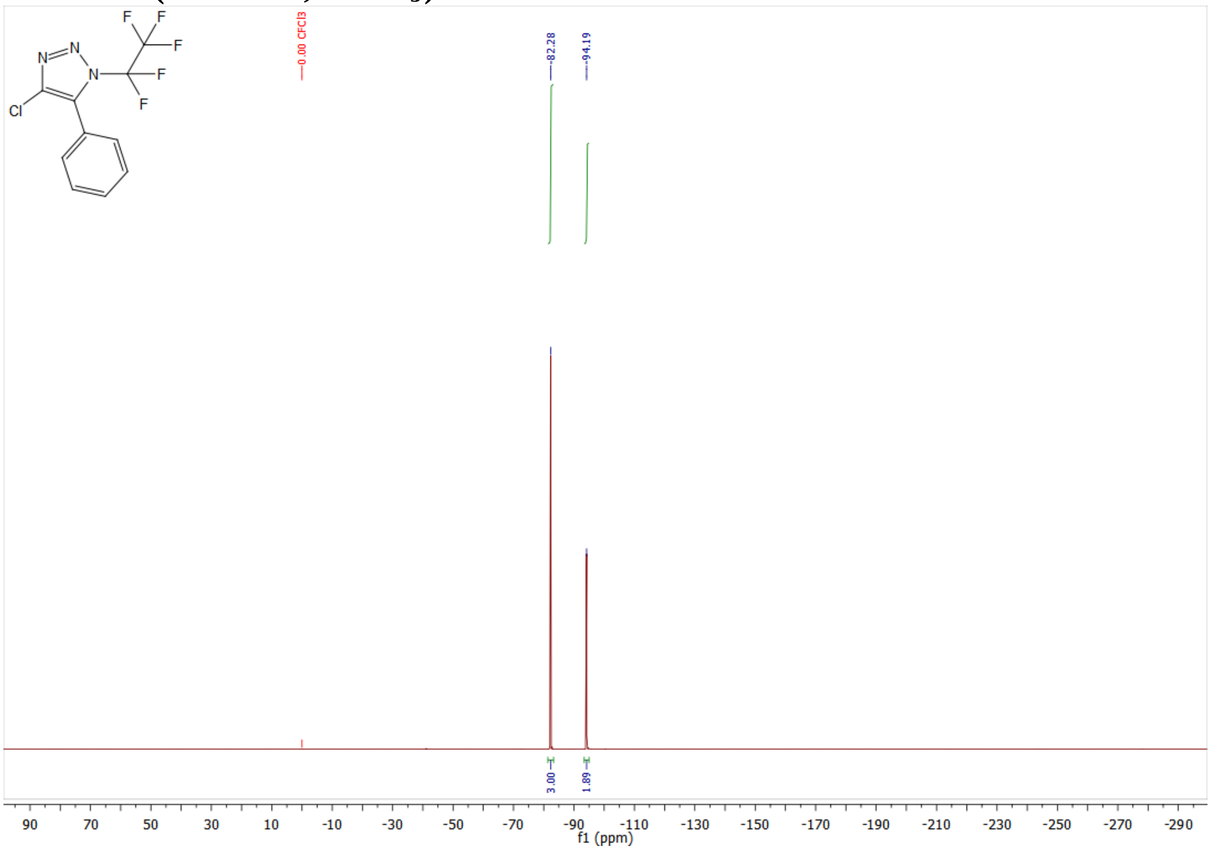

^{1}H NMR (400 MHz, $CDCl_3$) of 1w

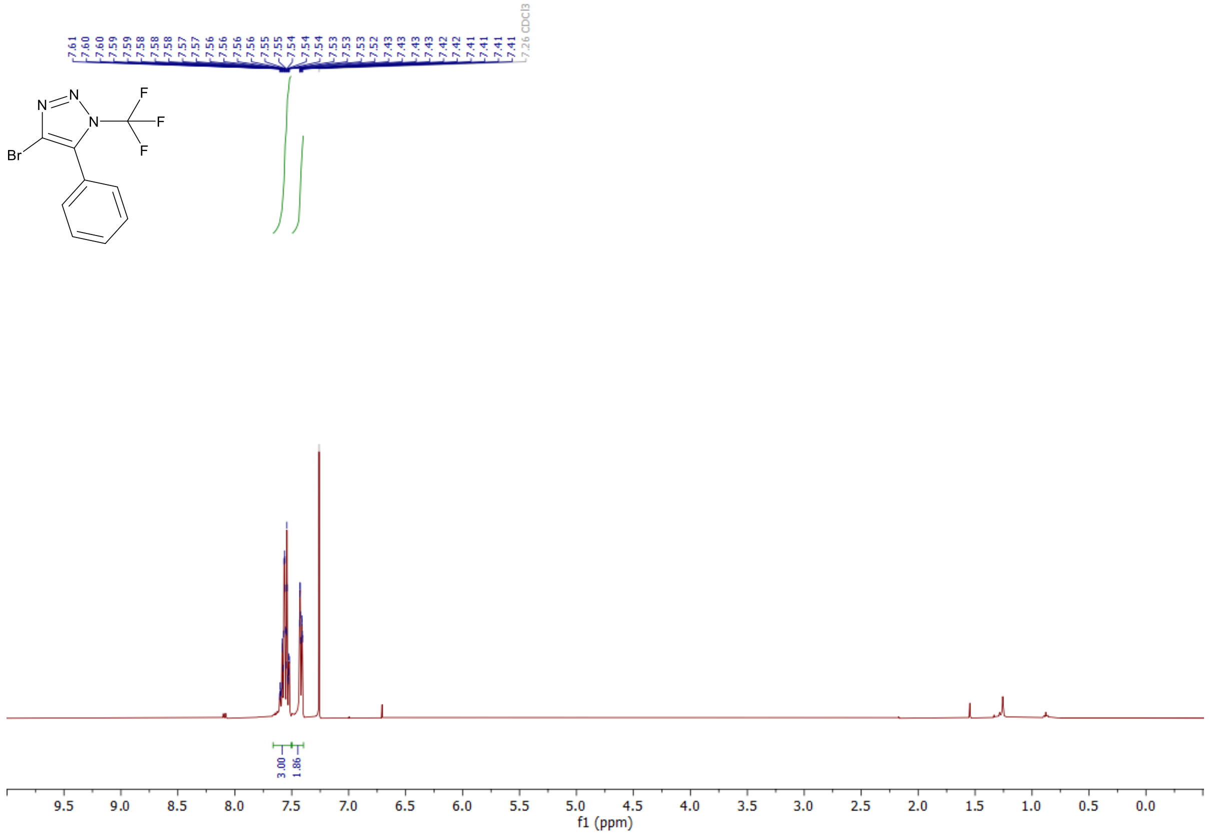

^{13}C {^{1}H} NMR (101 MHz, $CDCl_3$) of 1w

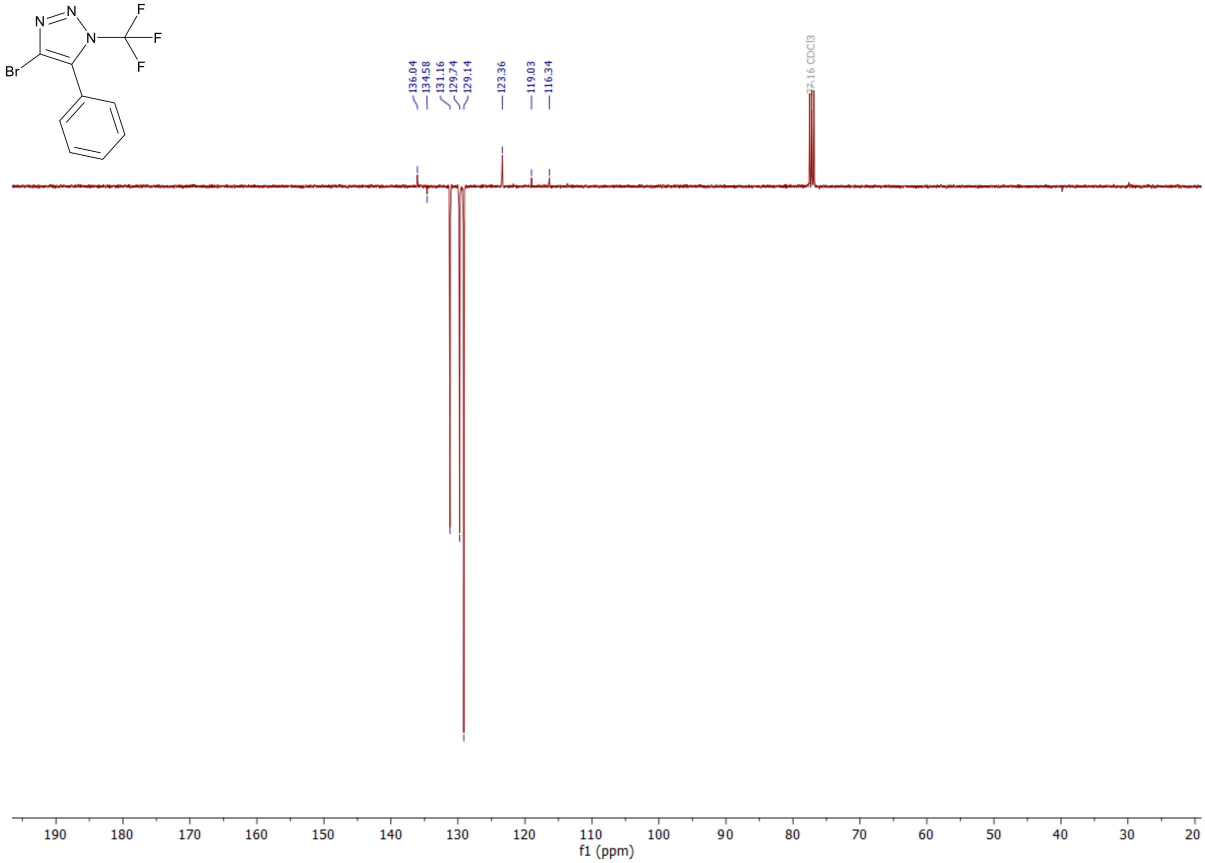

^{19}F NMR (377 MHz, $CDCl_3$) of 1w

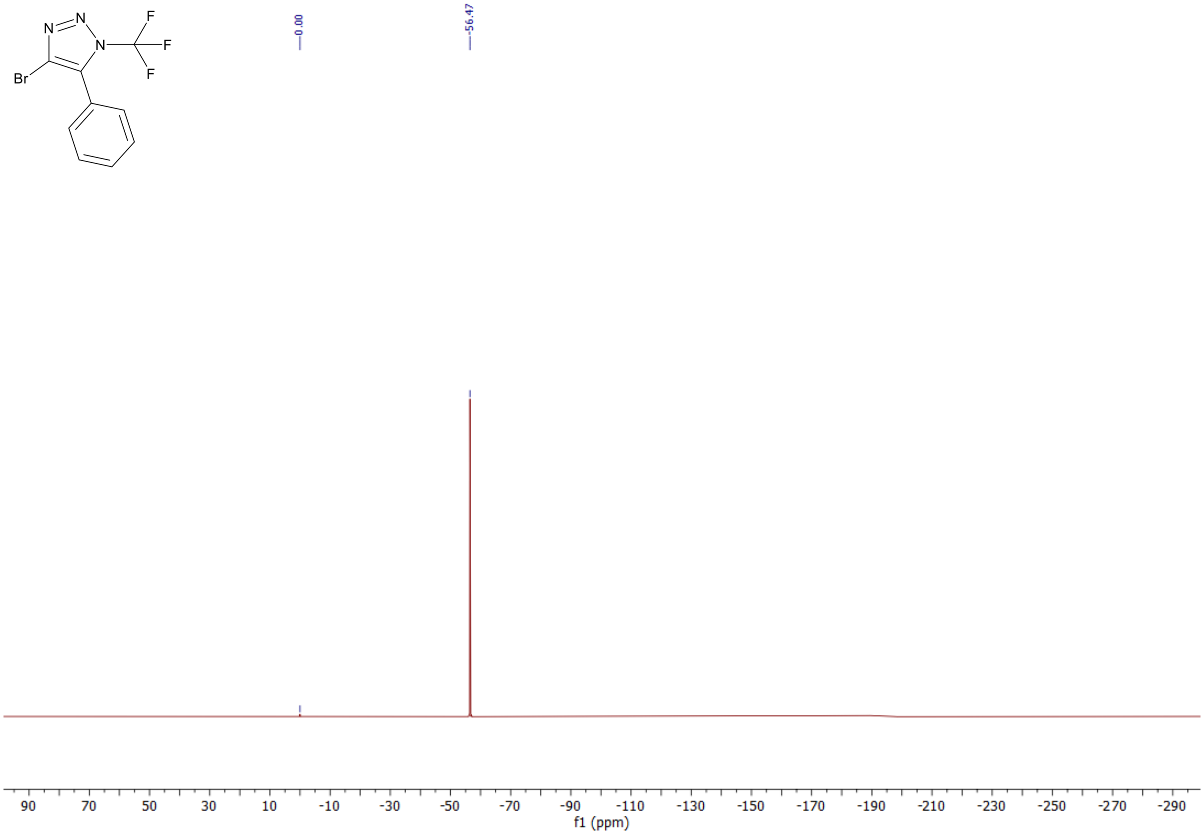

^{1}H NMR (400 MHz, $CDCl_3$) of 1x

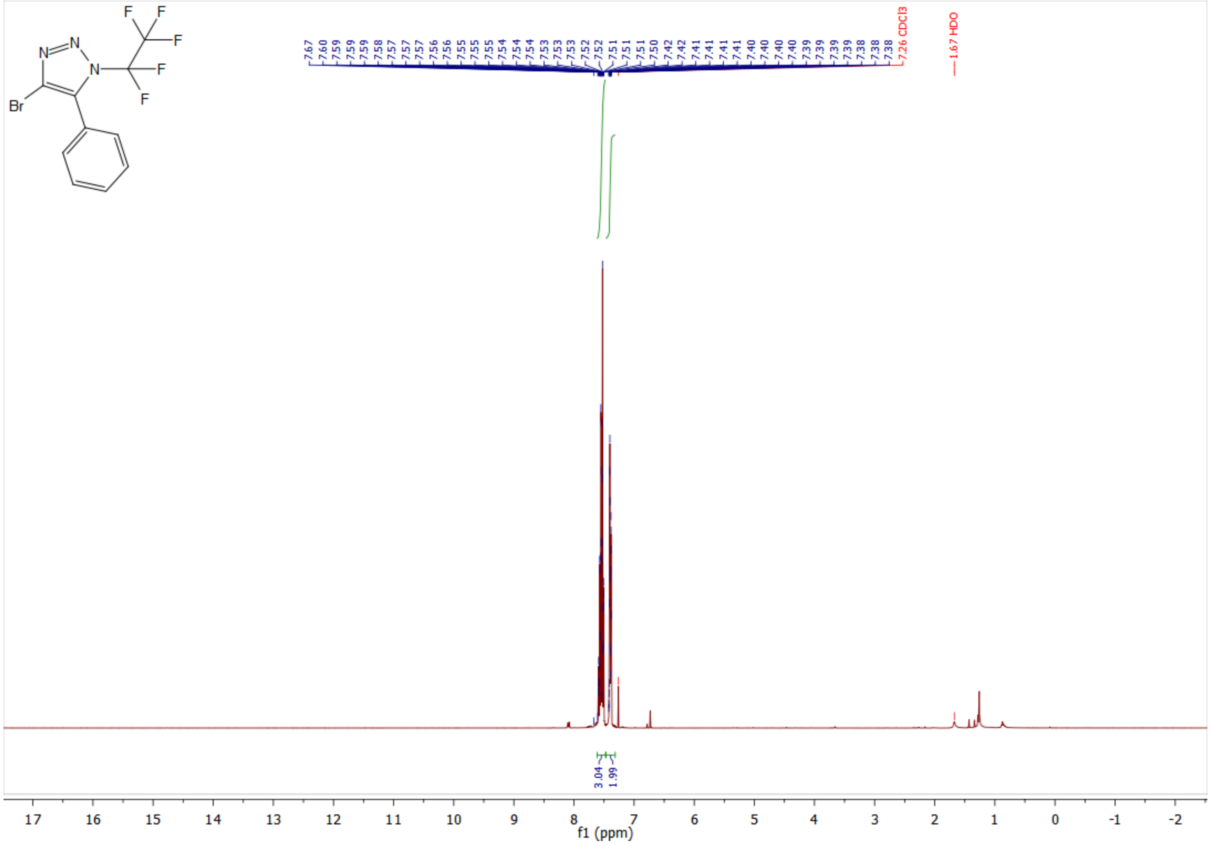

^{13}C {^{1}H} NMR (101 MHz, $CDCl_3$) of 1x

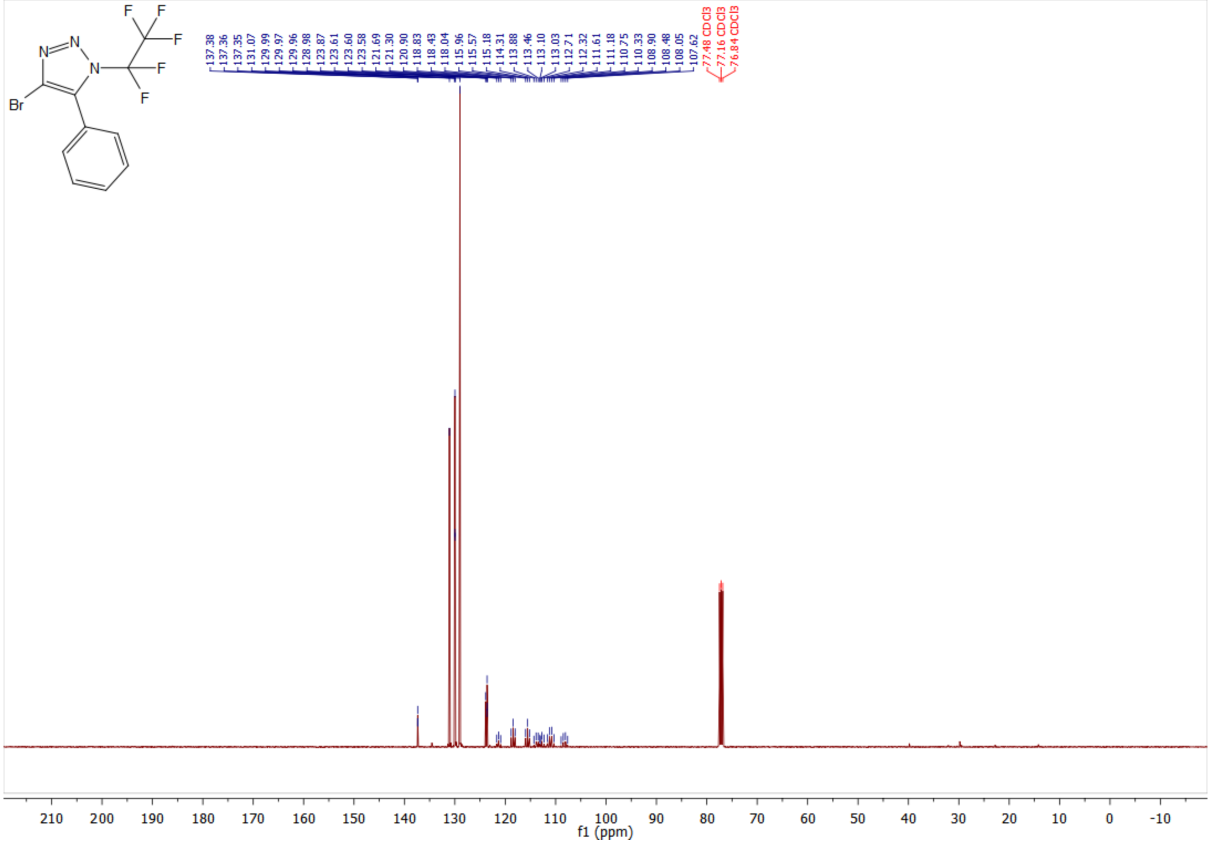

^{19}F NMR (377 MHz, $CDCl_3$) of 1x

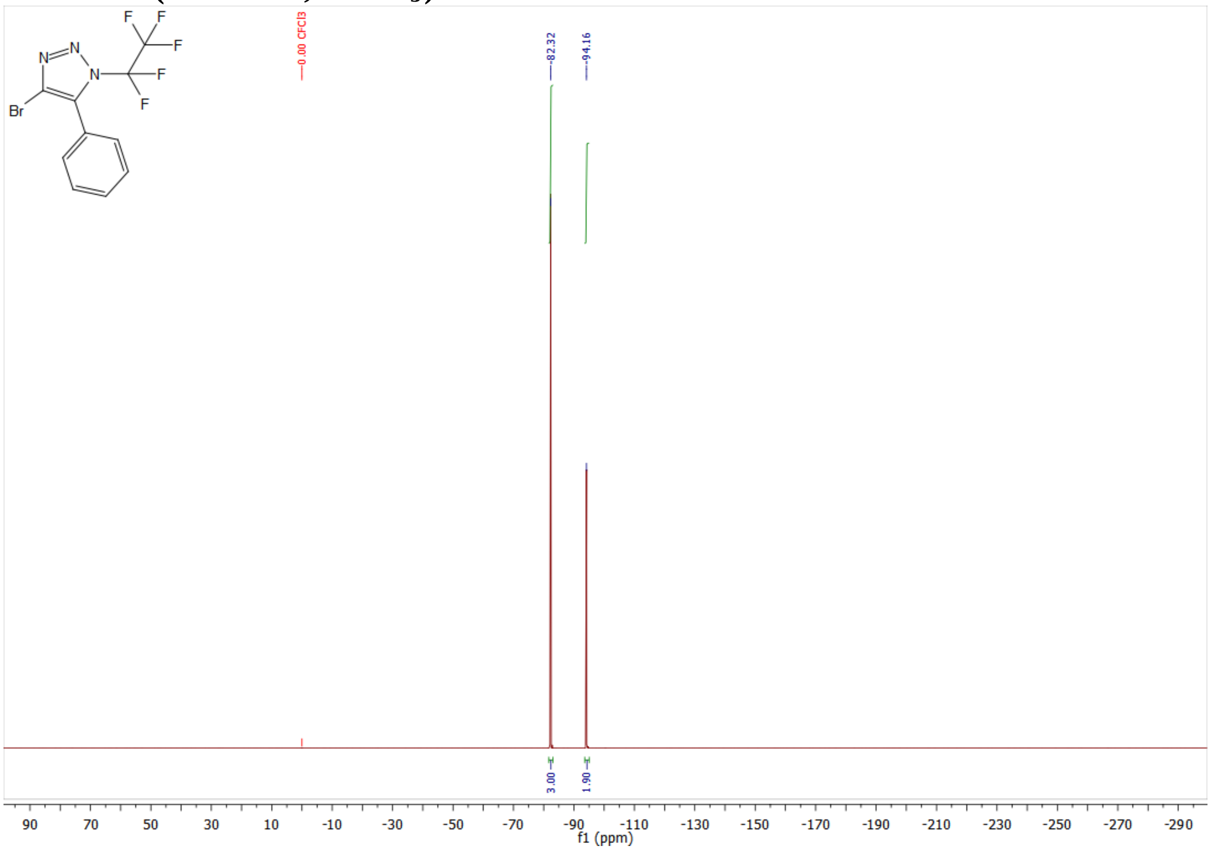

^{1}H NMR (400 MHz, $CDCl_3$) of 1y

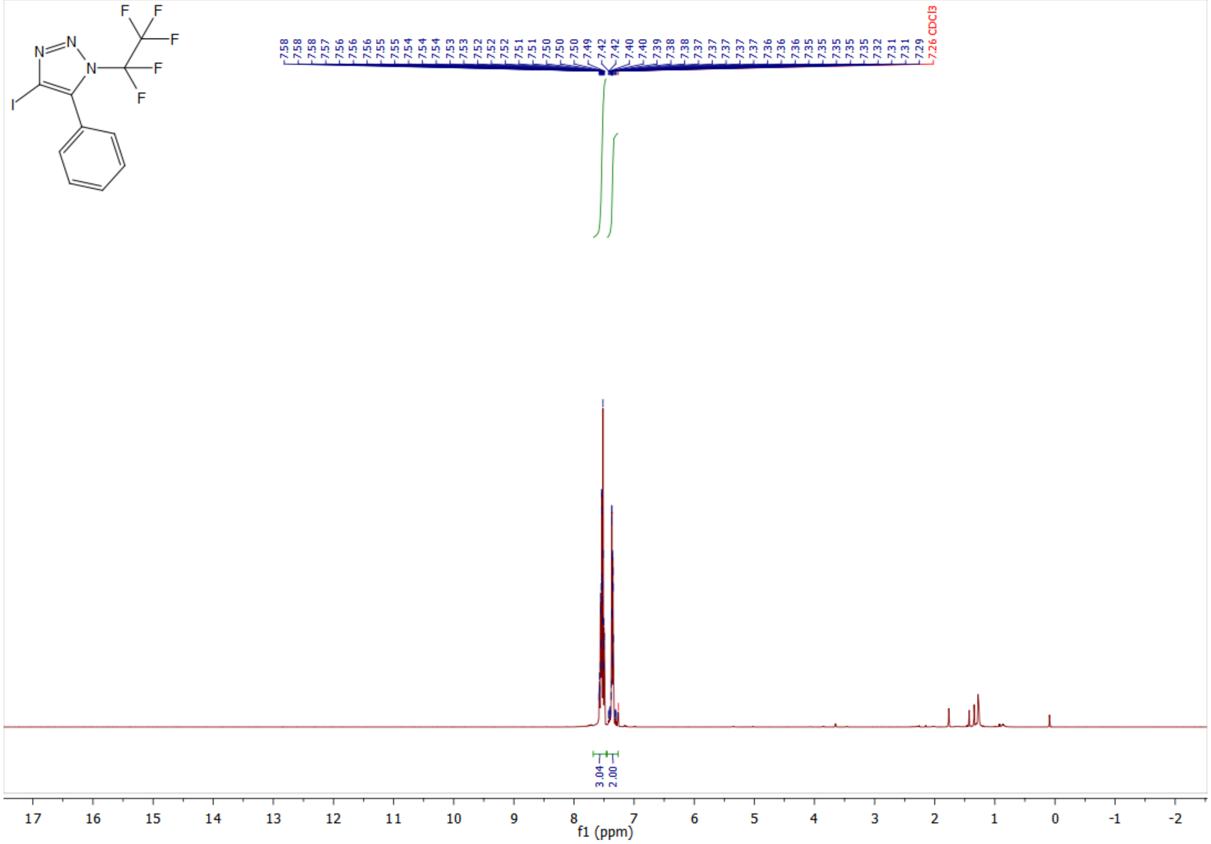

^{13}C {^{1}H} NMR (101 MHz, $CDCl_3$) of 1y

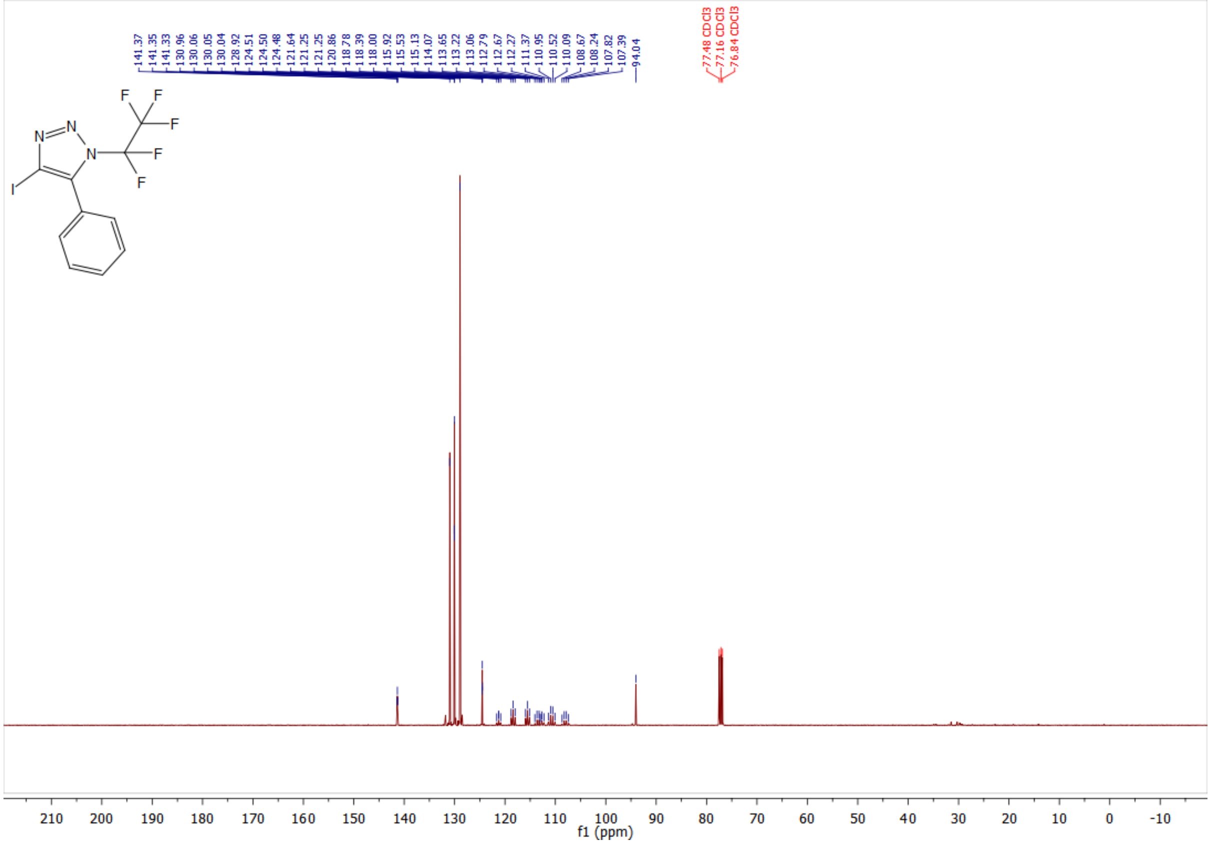

^{19}F NMR (377 MHz, $CDCl_3$) of 1y

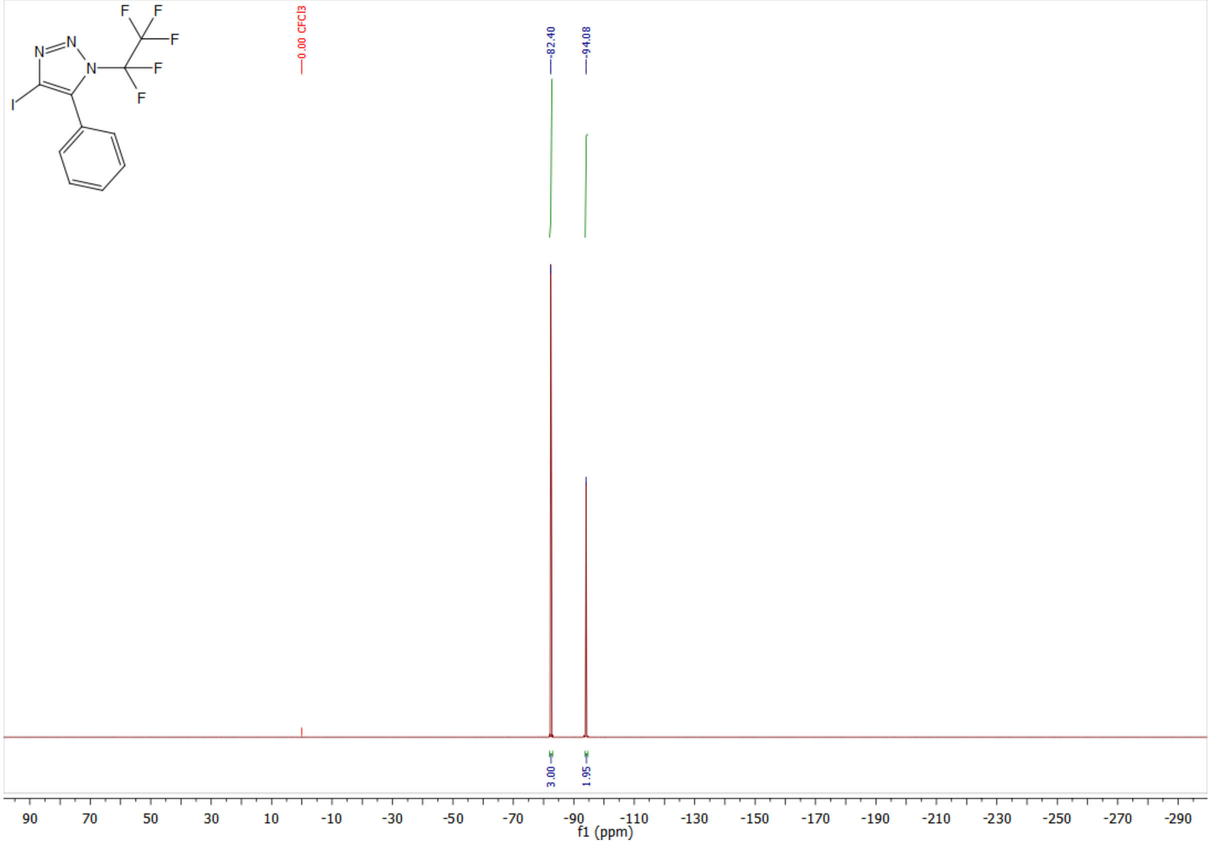

^{1}H NMR (400 MHz, $CDCl_3$) of 1z

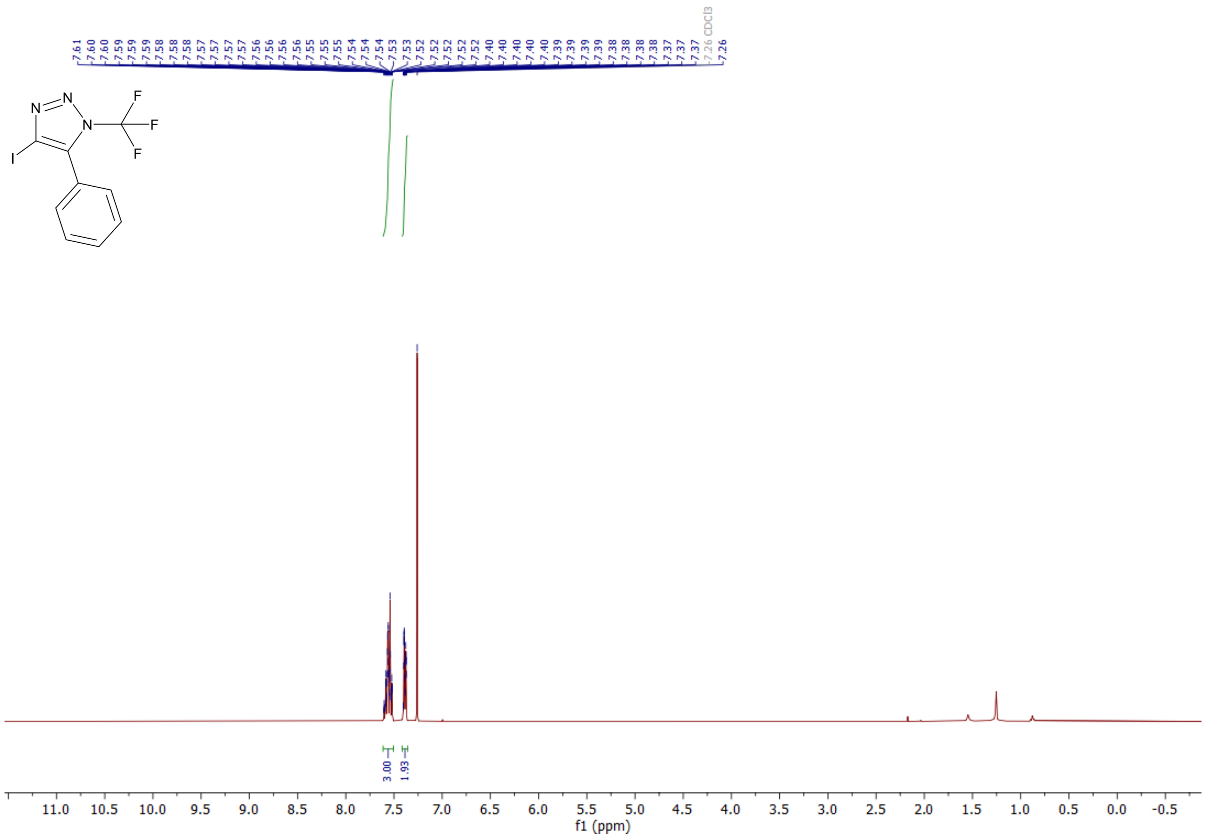

^{13}C {^{1}H} NMR (101 MHz, $CDCl_3$) of 1z

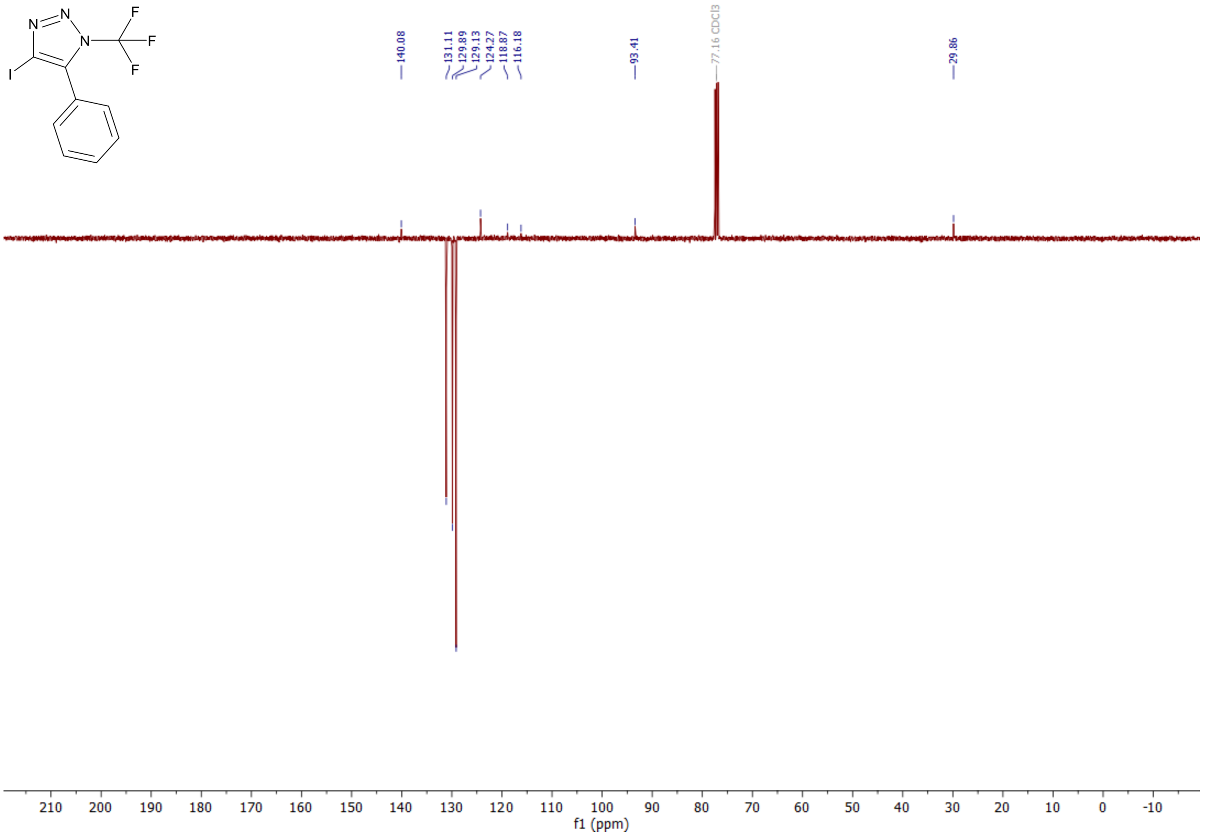

^{19}F NMR (377 MHz, $CDCl_3$) of 1z

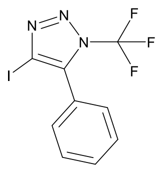

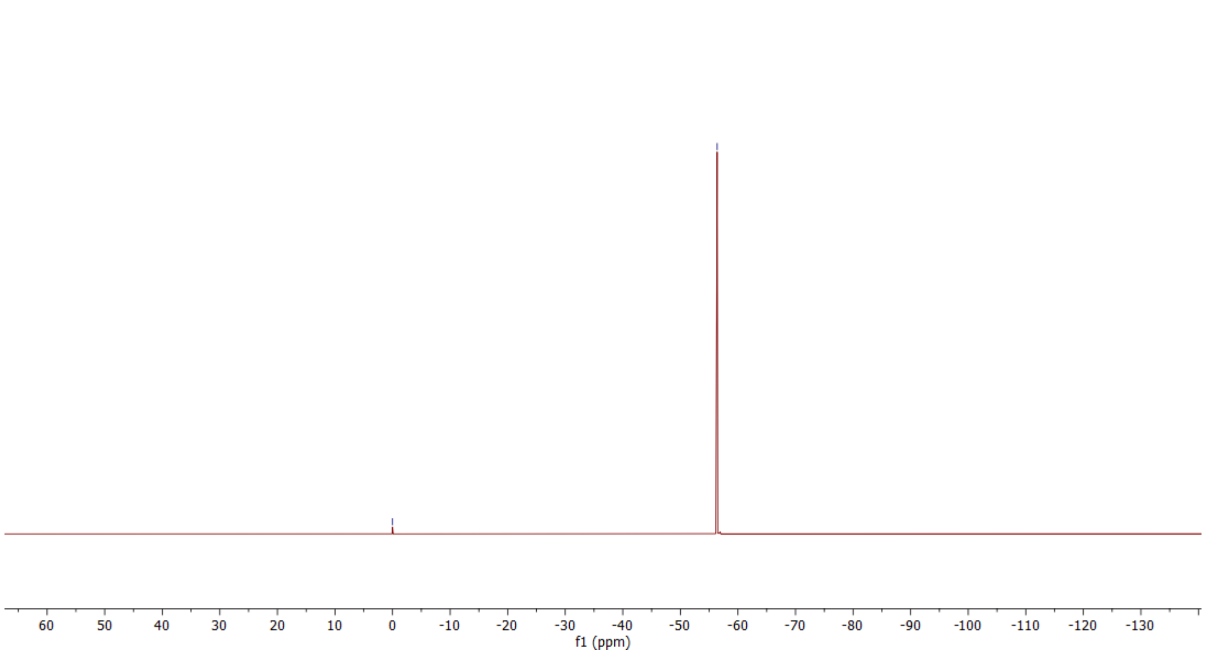

^{1}H NMR (400 MHz, $CDCl_3$) of 4v

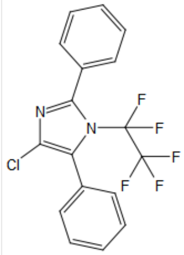

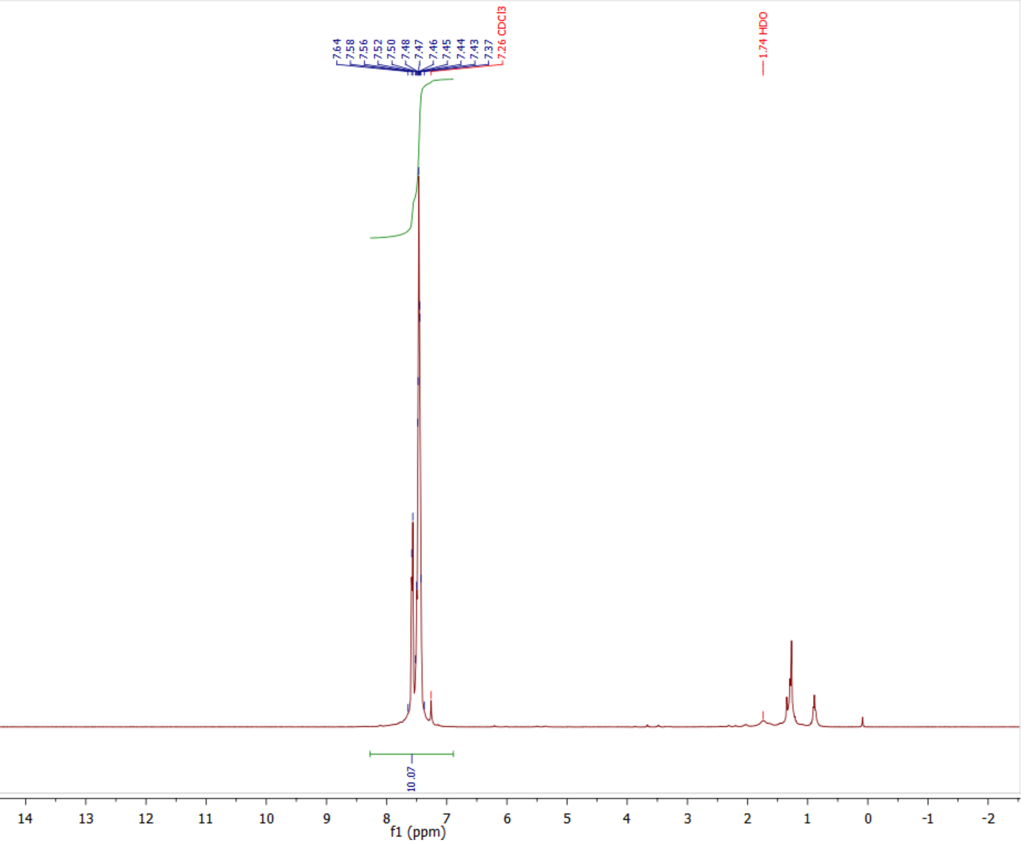

$^{13}C\ \{^{1}H\}$ NMR (101 MHz, $CDCl_3$) of 4v

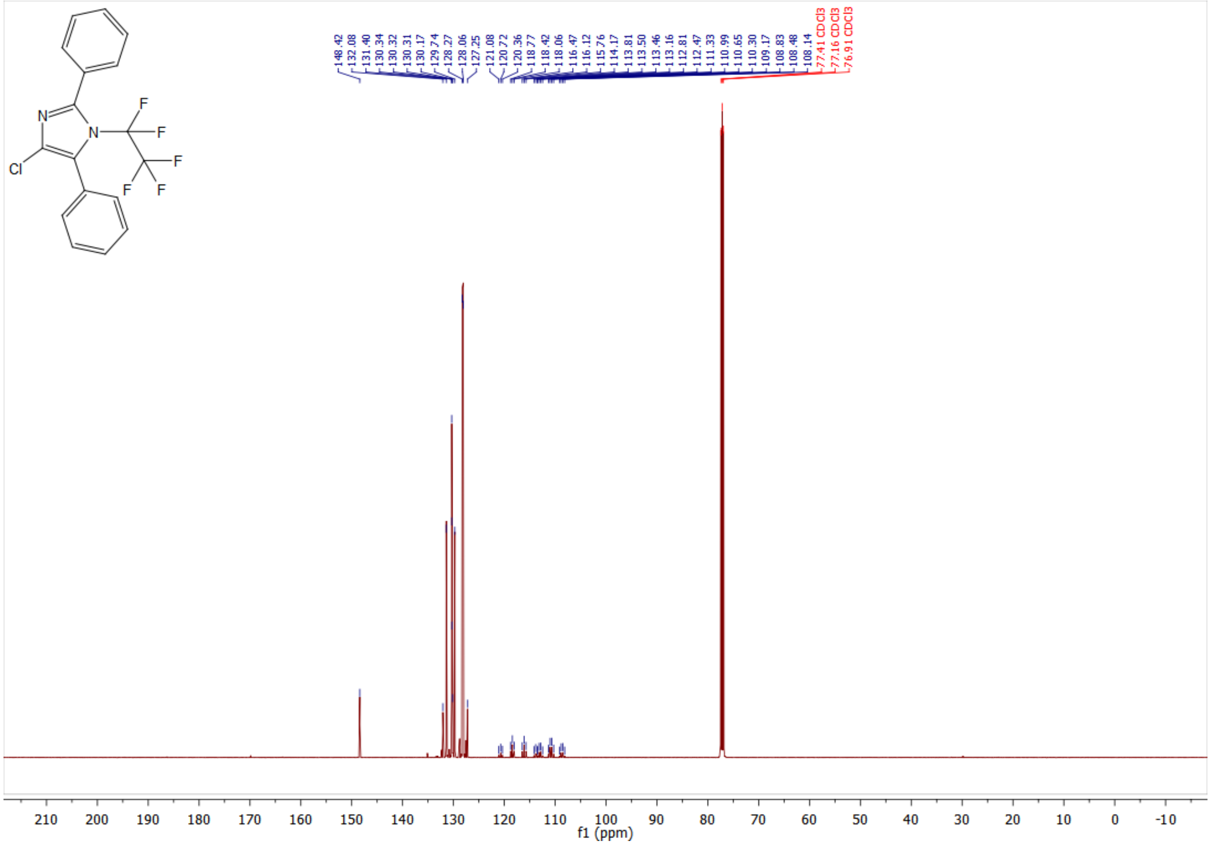

^{19}F NMR (377 MHz, $CDCl_3$) of 4v

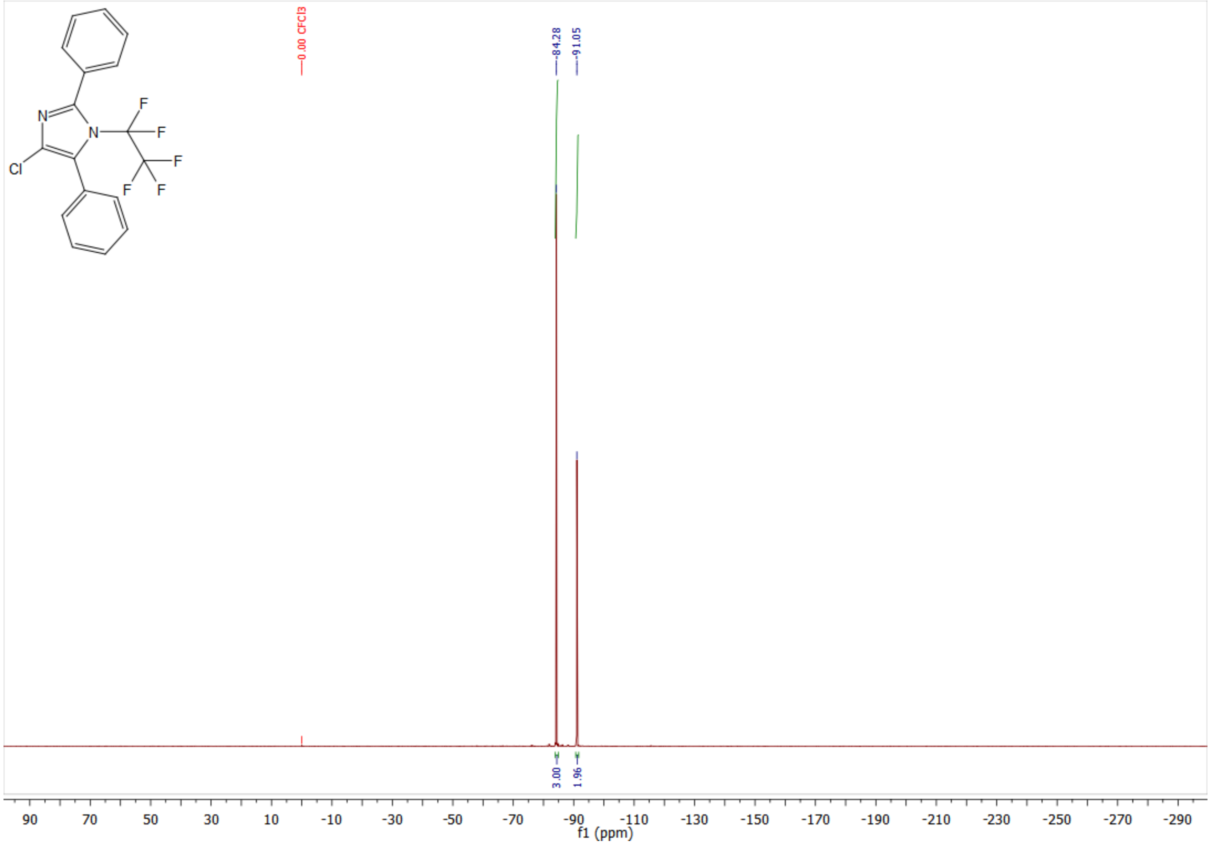

^{1}H NMR (400 MHz, $CDCl_3$) of 4x

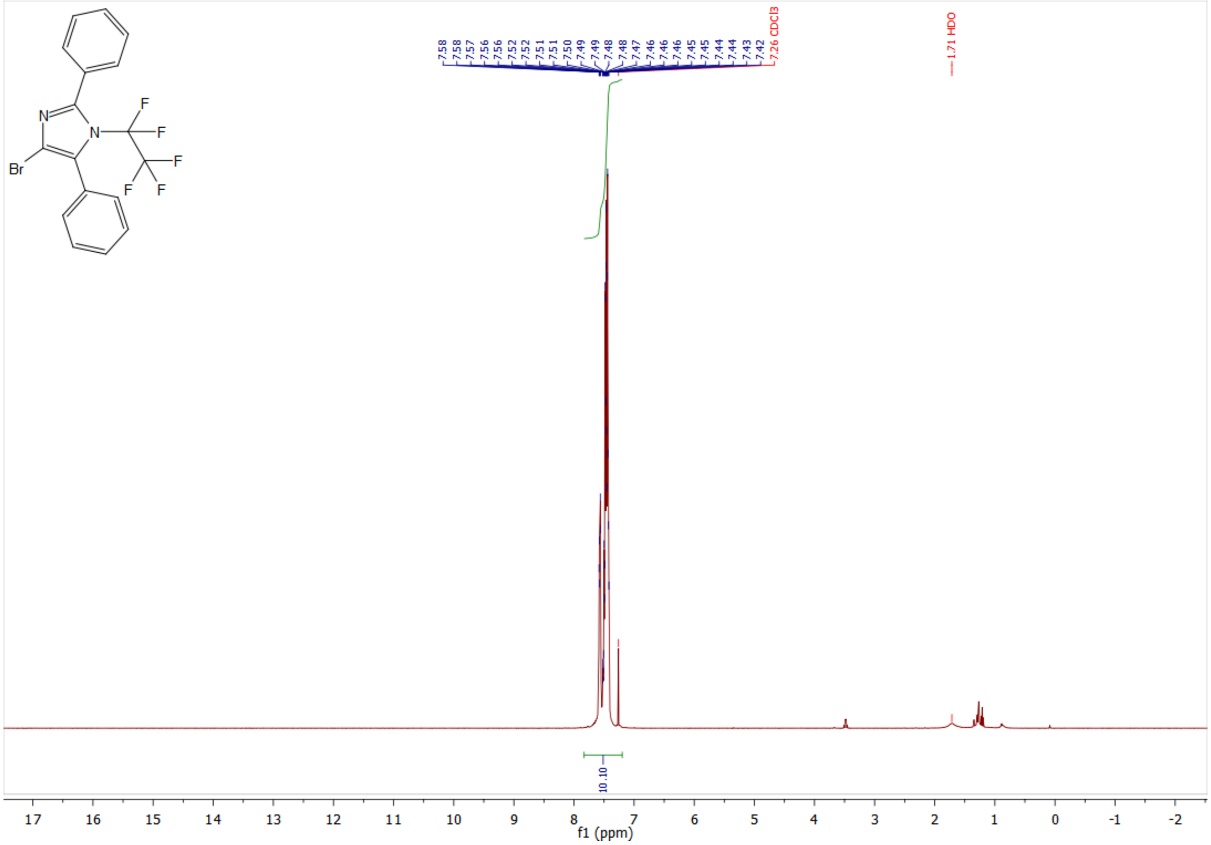

^{13}C {^{1}H} NMR (101 MHz, $CDCl_3$) of 4x

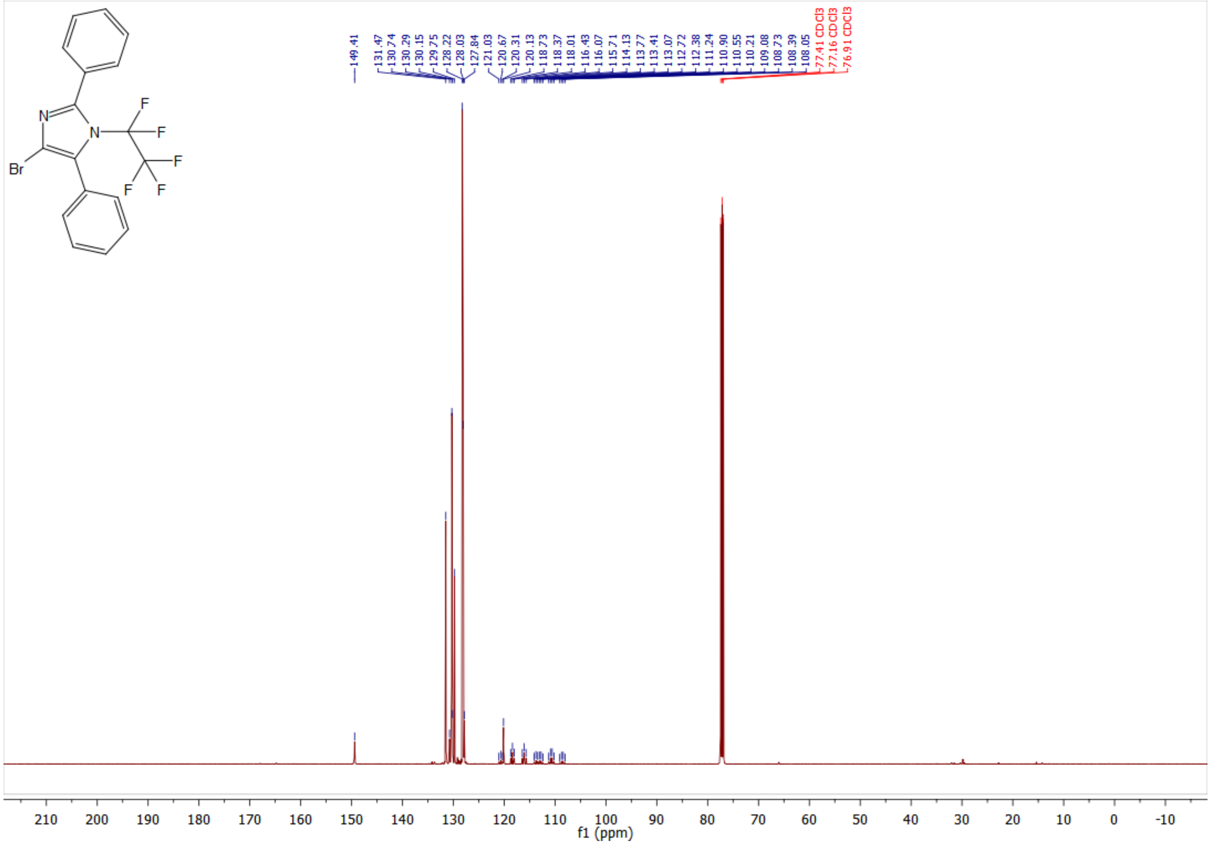

^{19}F NMR (377 MHz, $CDCl_3$) of 4x

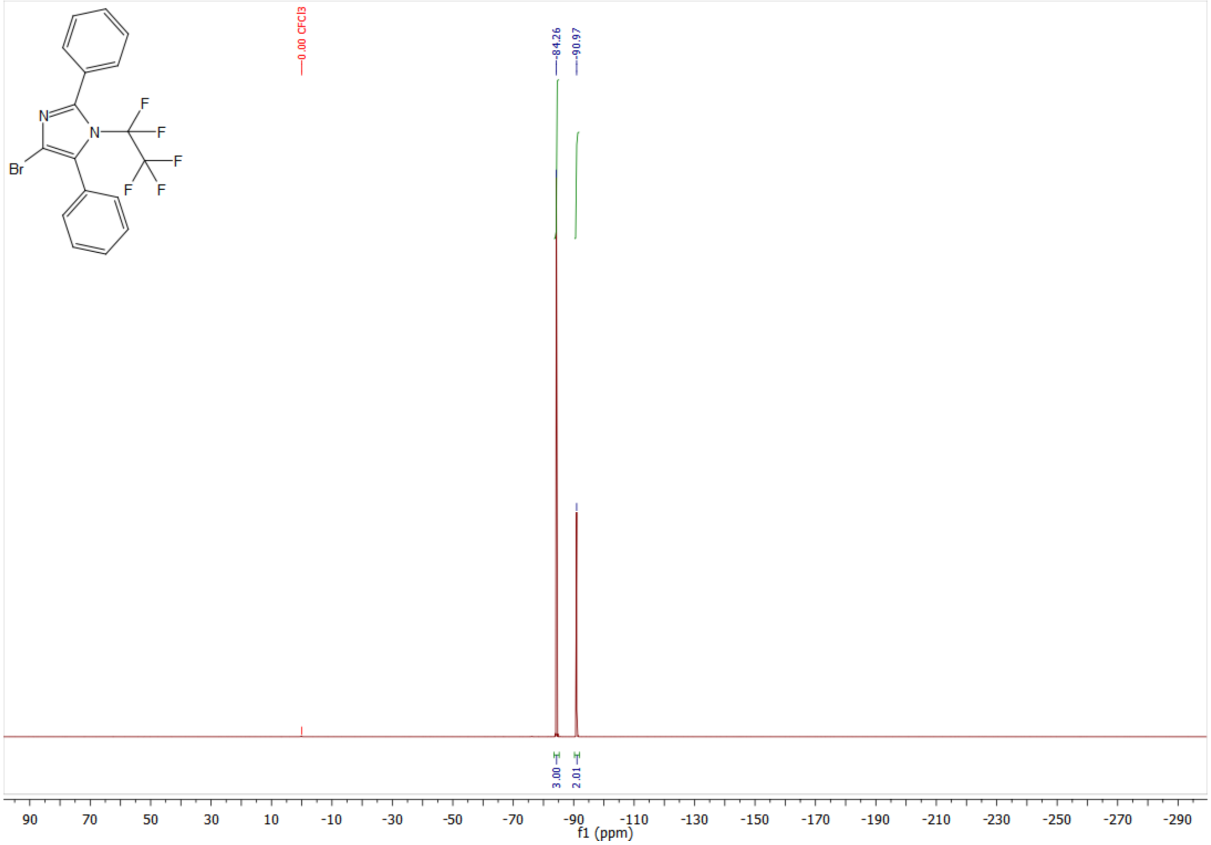

^{1}H NMR (400 MHz, $CDCl_3$) of 4y

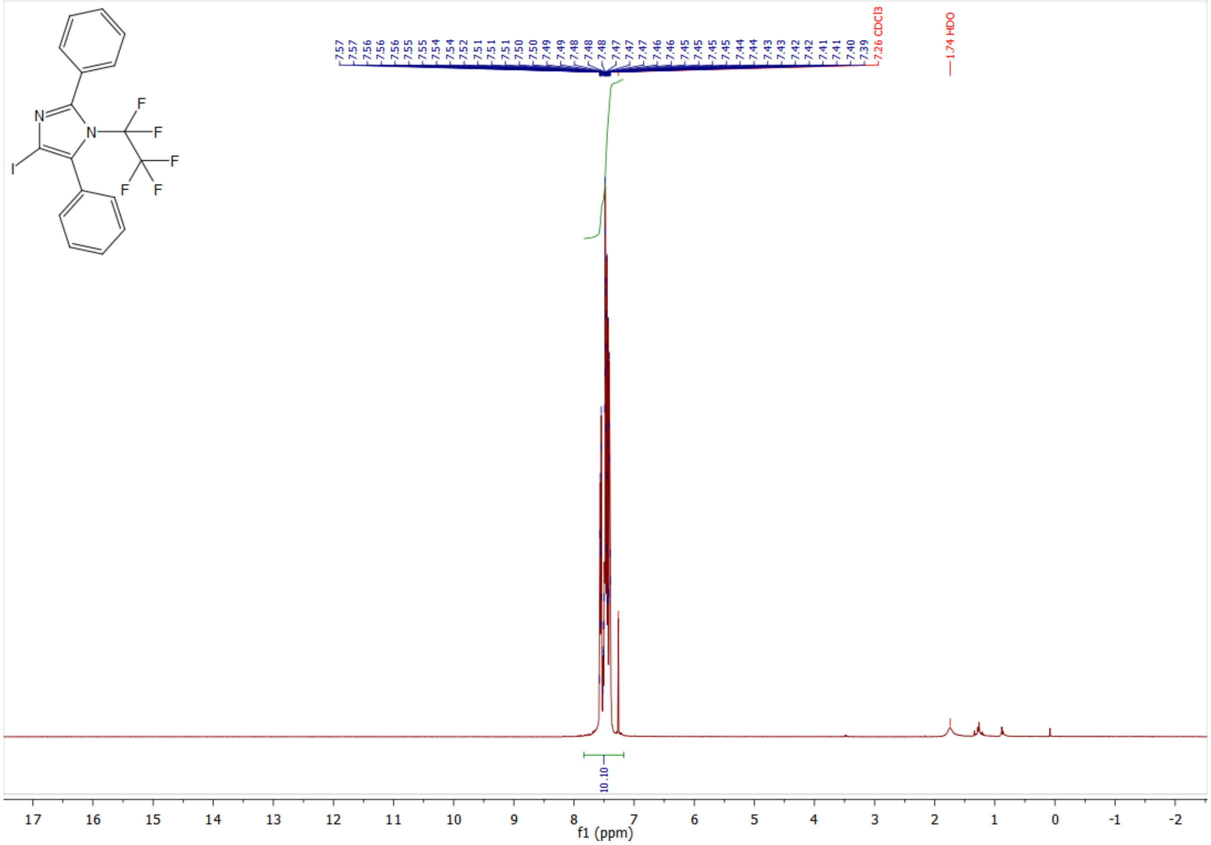

$^{13}C\ \{^1H\}$ NMR (101 MHz, $CDCl_3$) of 4y

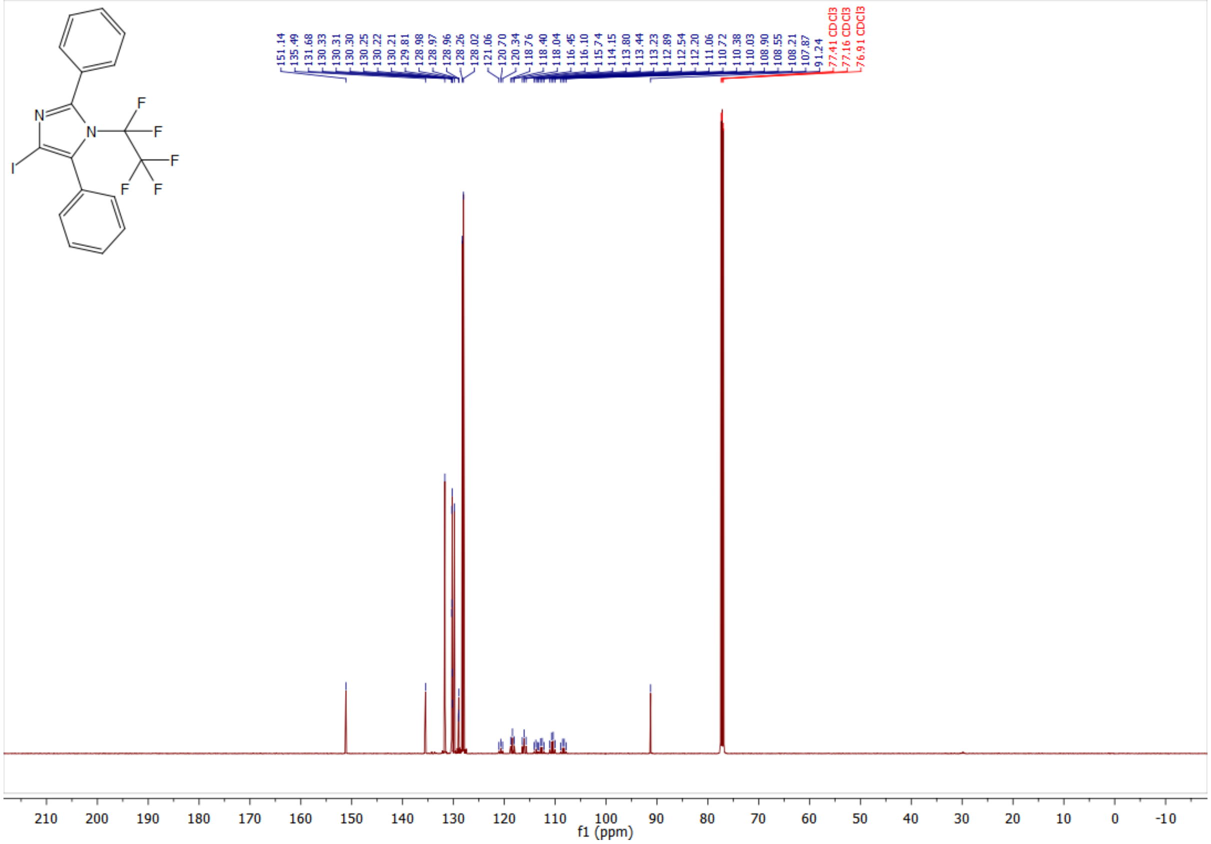

^{19}F NMR (377 MHz, $CDCl_3$) of 4y

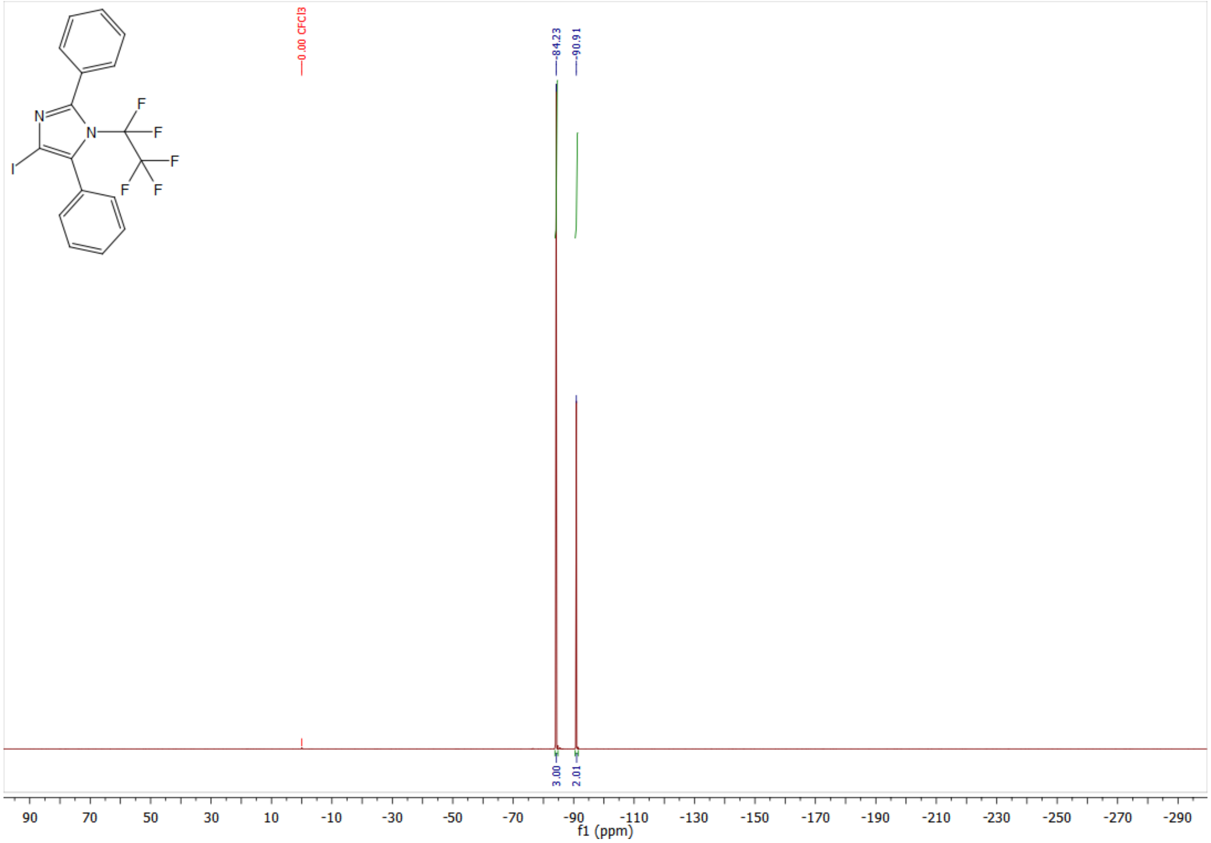

^{1}H NMR (400 MHz, $CDCl_3$) of 5w

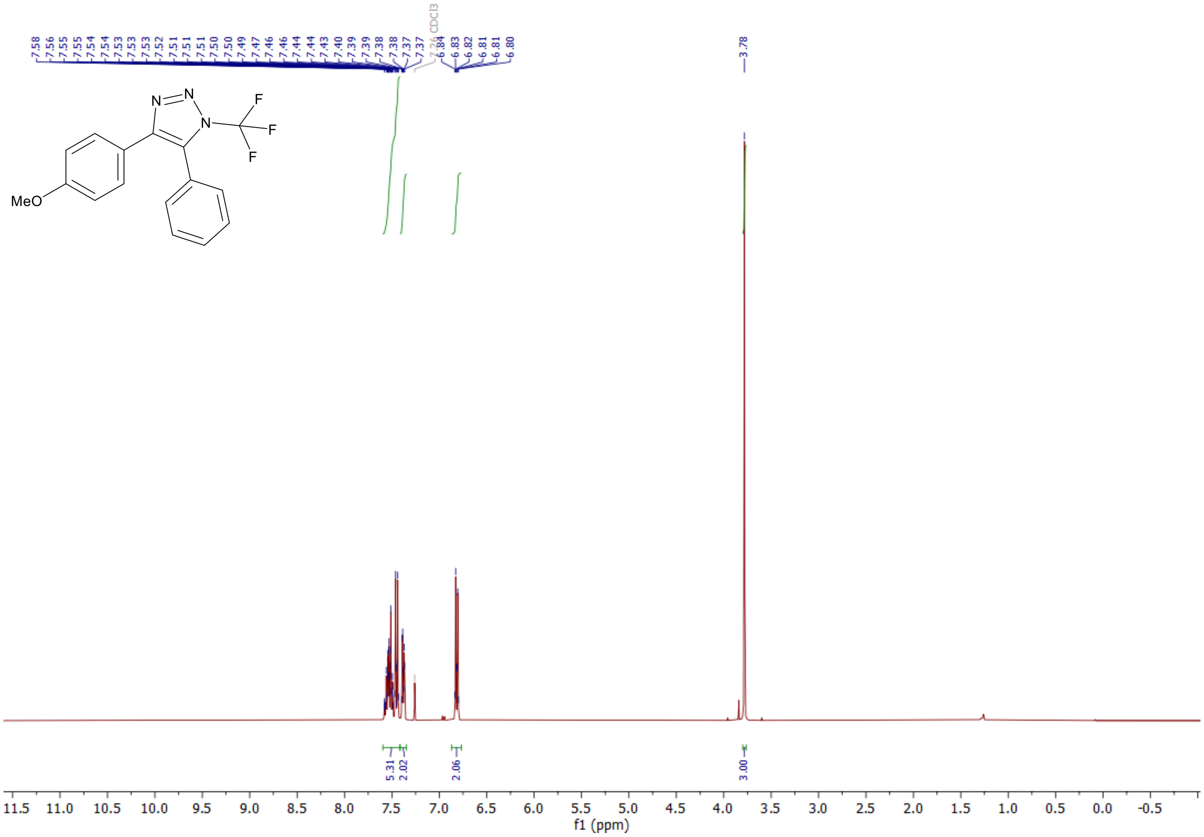

^{13}C {^{1}H} NMR (101 MHz, $CDCl_3$) of 5w

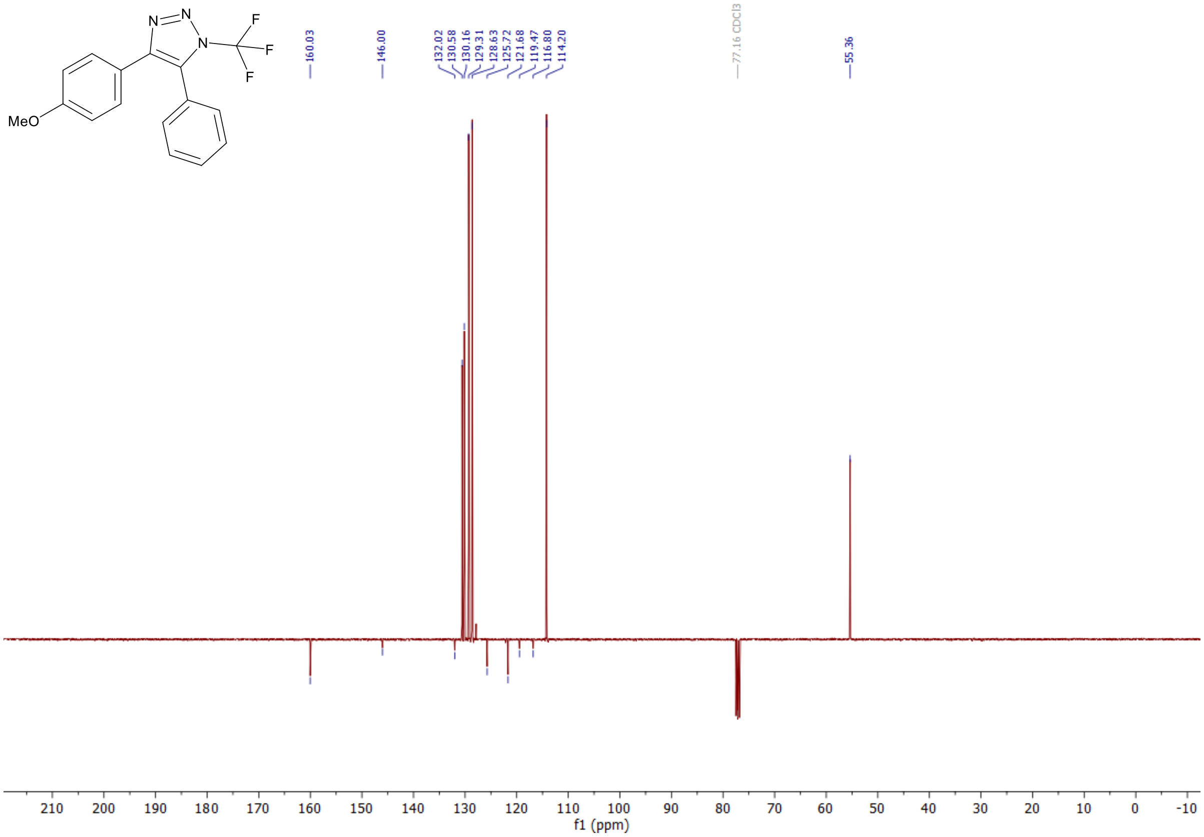

^{19}F NMR (377 MHz, CDCl$_3$) of 5w

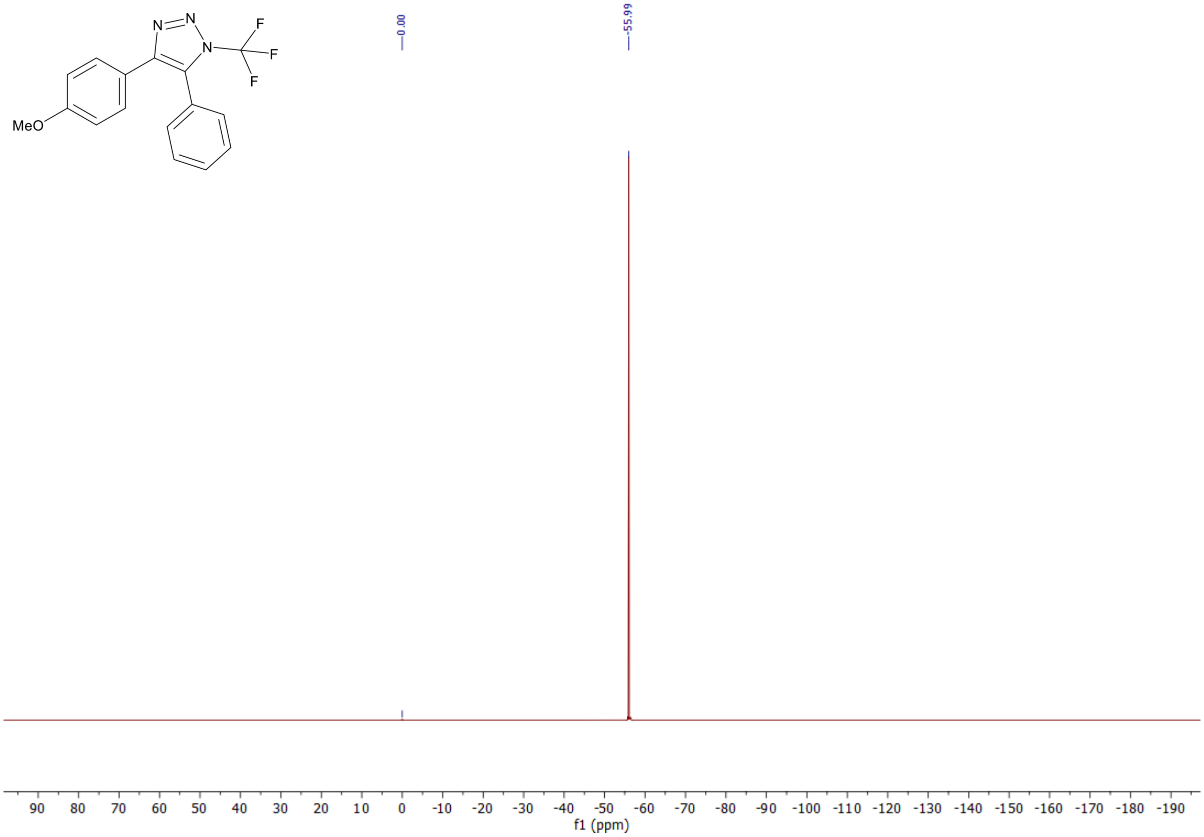

^{1}H NMR (400 MHz, CDCl$_3$) of 5x

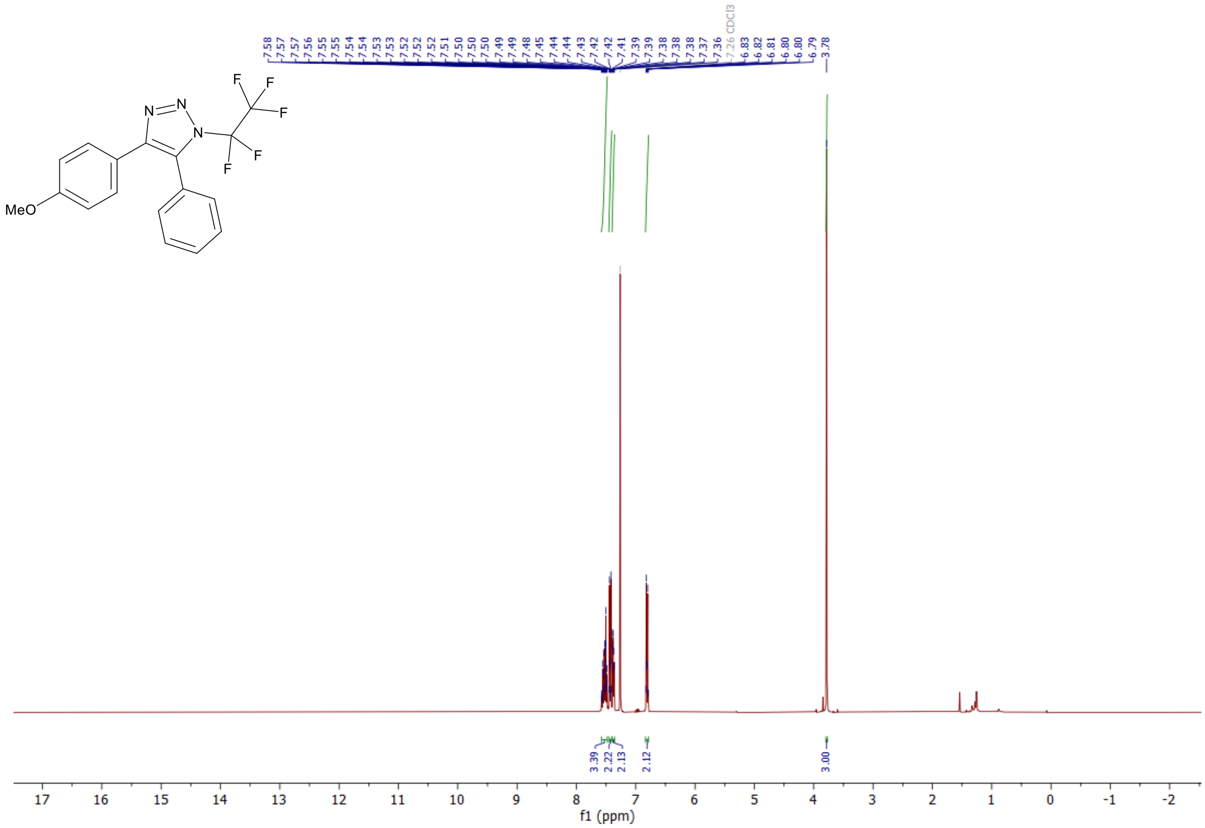

$^{13}C\{^1H\}$ NMR (126 MHz, $CDCl_3$) of 5x

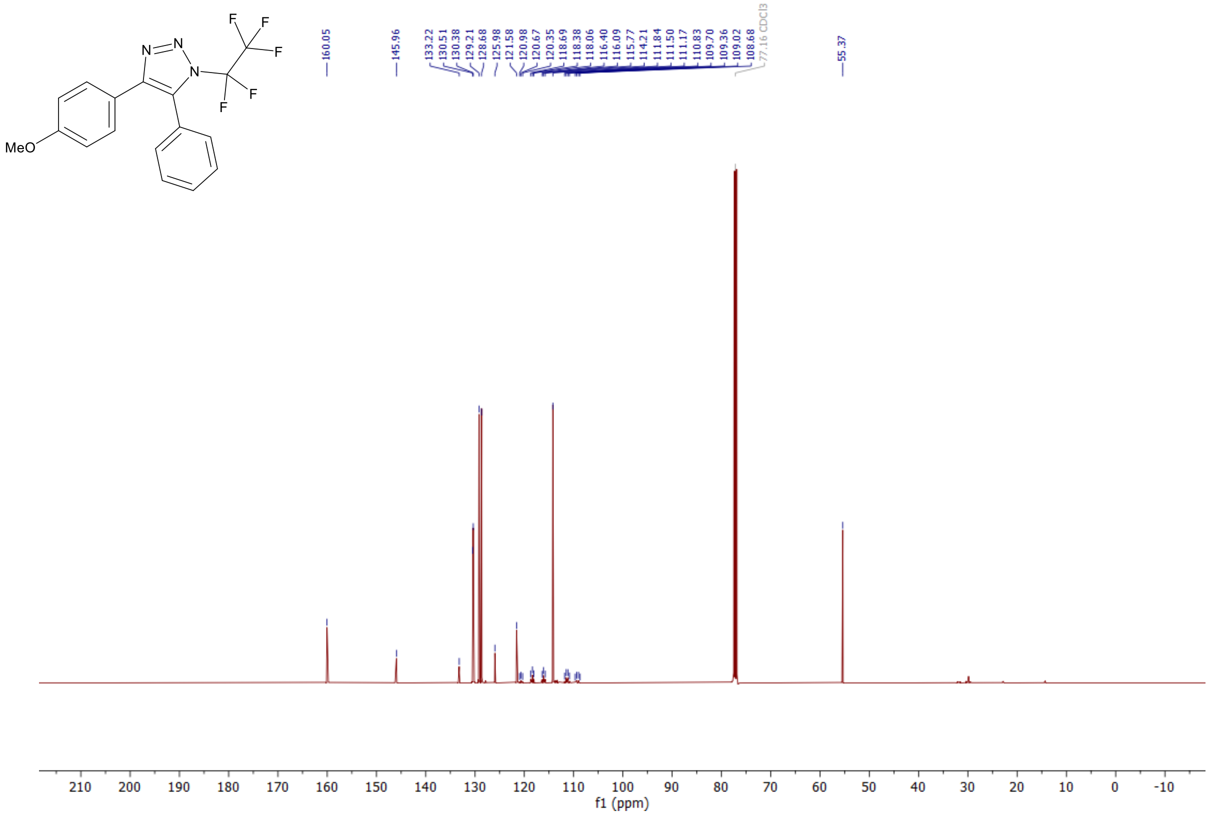

^{19}F NMR (377 MHz, $CDCl_3$) of 5x

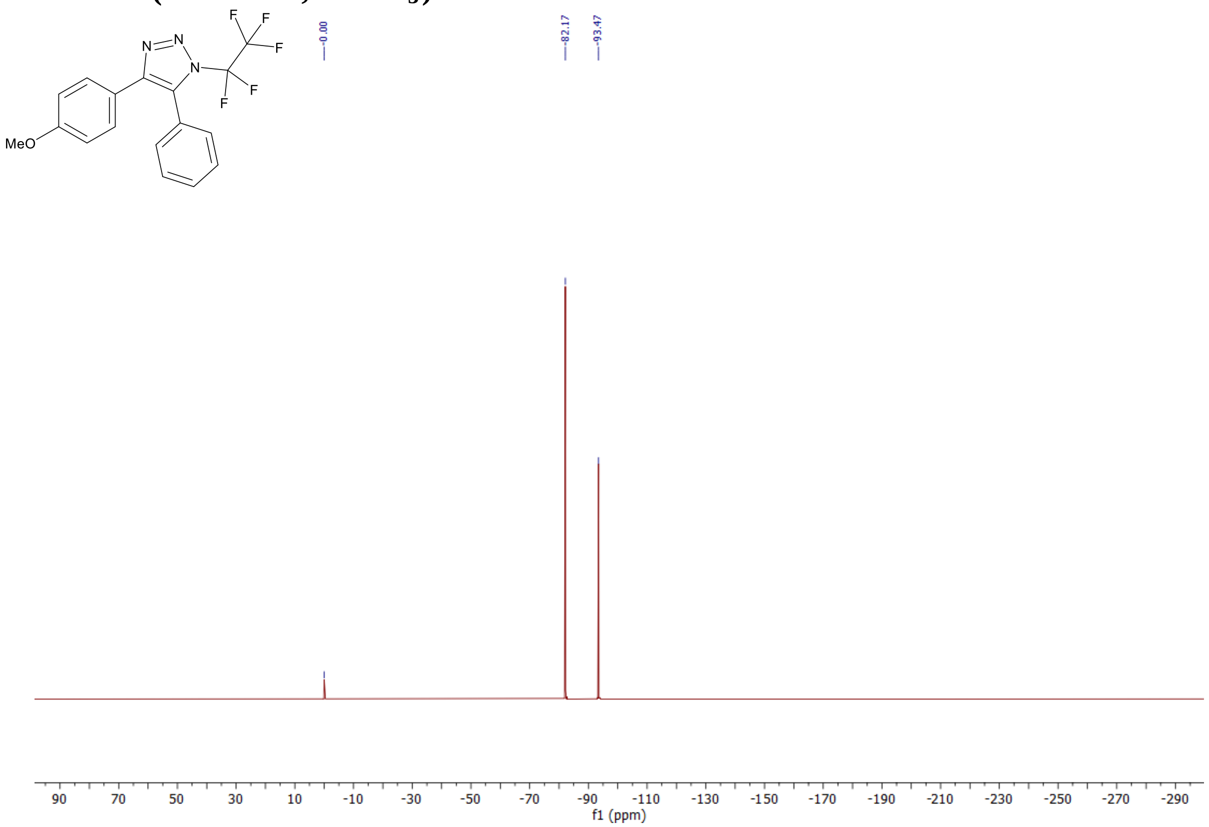

^{1}H NMR (400 MHz, $CDCl_3$) of 6z

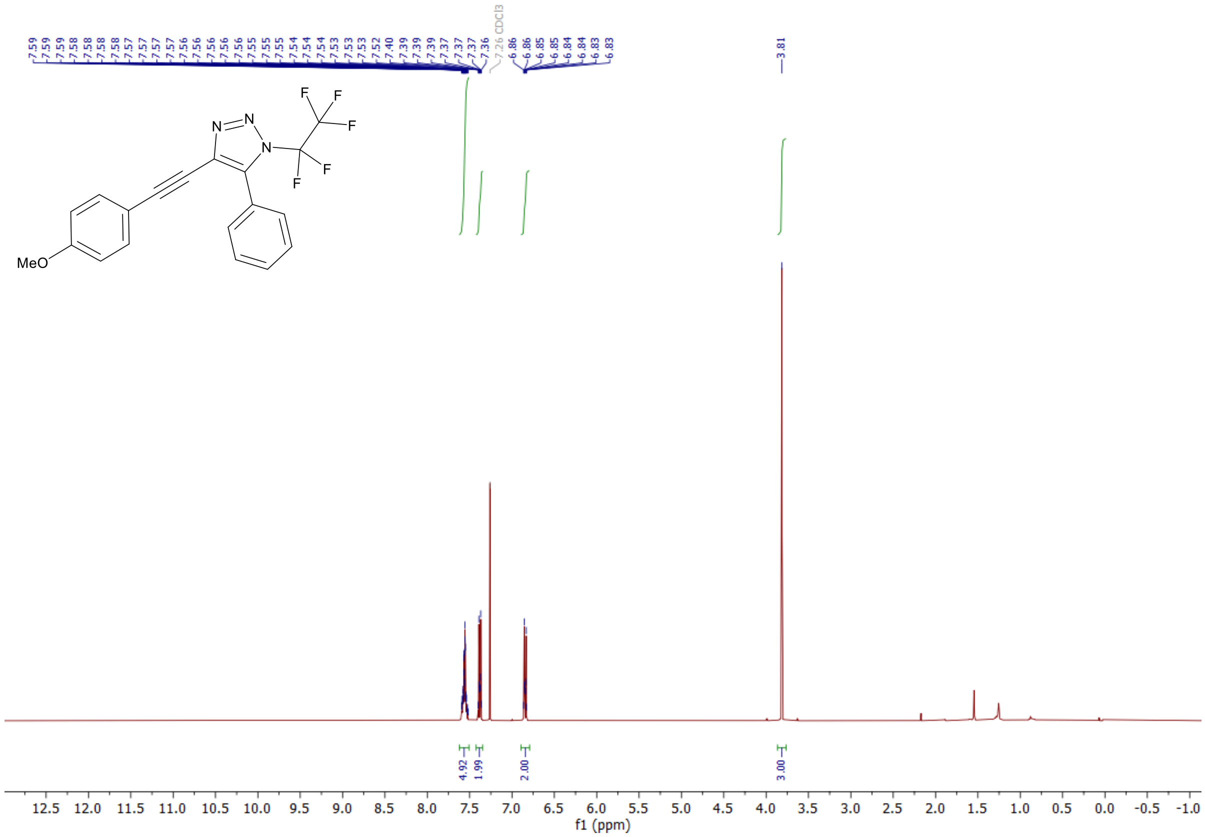

^{13}C {^{1}H} NMR (126 MHz, $CDCl_3$) of 6z

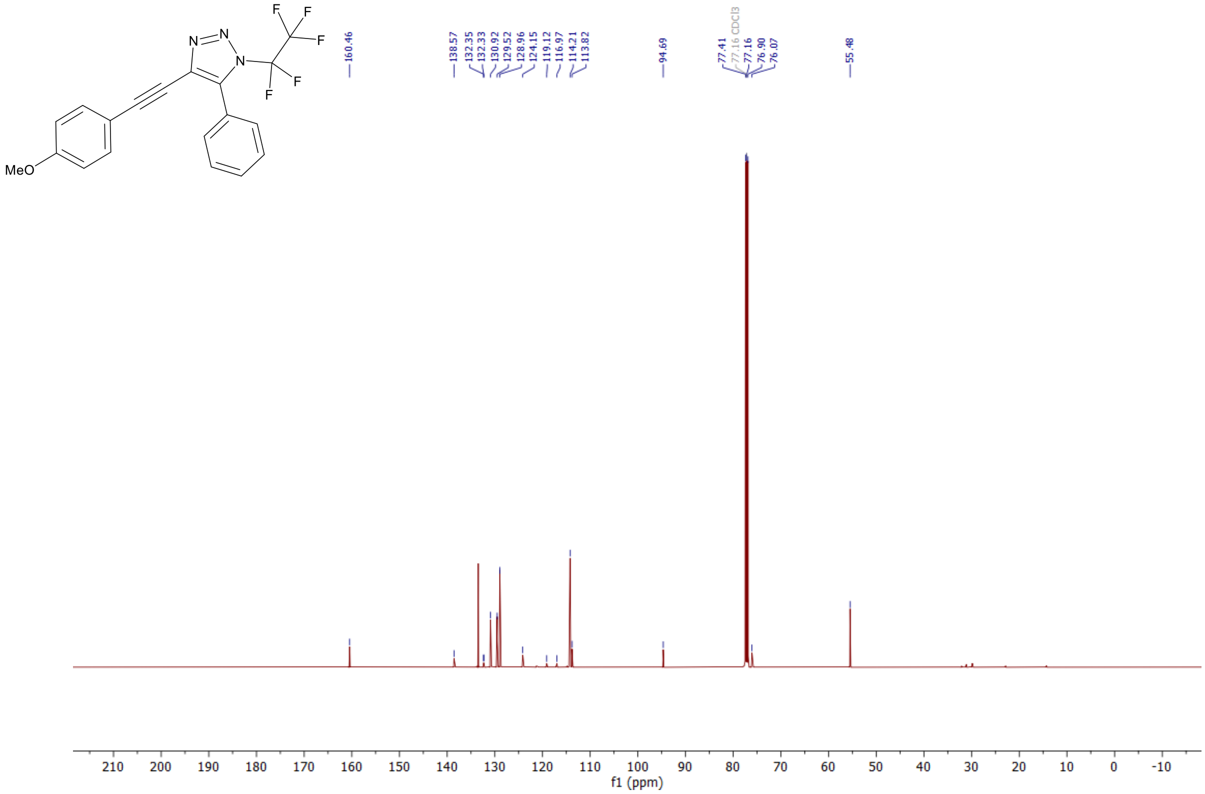

^{19}F NMR (377 MHz, $CDCl_3$) of 6z

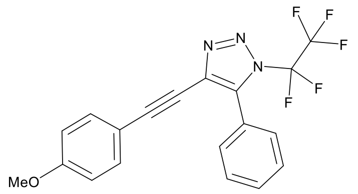

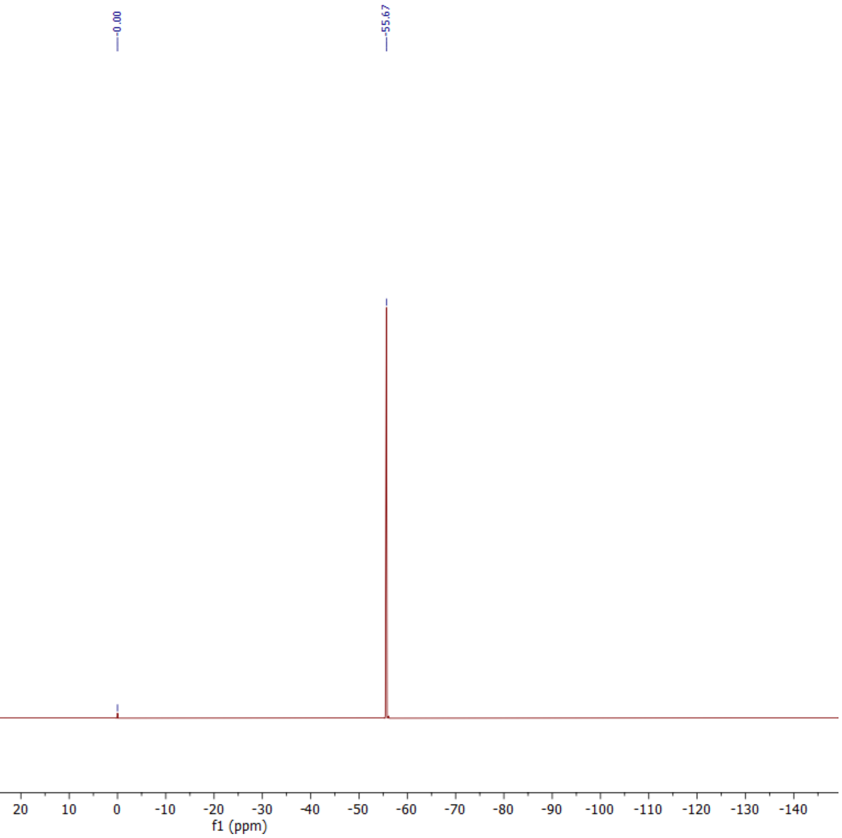

Supplement: Supplementary file 1 [file jo5c01055_si_001.pdf]
